# Dietary Palmitic Acid Drives a Palmitoyltransferase ZDHHC15‐YAP Feedback Loop Promoting Tumor Metastasis

**DOI:** 10.1002/advs.202409883

**Published:** 2024-12-16

**Authors:** Jianxin Wang, Dachuan Shen, Jian Jiang, Lulu Hu, Kun Fang, Chunrui Xie, Ning Shen, Yuzhao Zhou, Yifei Wang, Sha Du, Songshu Meng

**Affiliations:** ^1^ Institute of Cancer Stem Cell Dalian Medical University Cancer Center Dalian 116044 China; ^2^ Department of Oncology Affiliated Zhongshan Hospital of Dalian University Dalian 116001 China; ^3^ Central Hospital of Dalian University of Technology Department of Spine Surgery Dalian 116033 China; ^4^ Department of Laboratory Medicine Qingdao Central Hospital University of Health and Rehabilitation Sciences NO.369 Dengyun Road, Qingdao National High‐tech Industrial Development Zone Qingdao China; ^5^ Central Laboratory Cancer Hospital of China Medical University Cancer Hospital of Dalian University of Technology Liaoning Cancer Hospital & Institute Shenyang 110042 China; ^6^ Department of Obstetrics and Gynecology Affiliated Zhongshan Hospital of Dalian University Dalian 116001 China

**Keywords:** palmitic acids, S‐palmitoylations, tumor metastasis, YAP, ZDHHC15

## Abstract

Elevated uptake of saturated fatty acid palmitic acid (PA) is associated with tumor metastasis; however, the precise mechanisms remain partially understood, hindering the development of therapy for PA‐driven tumor metastasis. The Hippo–Yes‐associated protein (Hippo/YAP) pathway is implicated in cancer progression. Here it is shown that a high‐palm oil diet potentiates tumor metastasis in murine xenografts in part through YAP. It is found that the palmitoyltransferase ZDHHC15 is a YAP‐regulated gene that forms a feedback loop with YAP. Notably, PA drives the ZDHHC15‐YAP feedback loop, thus enforces YAP signaling, and hence promotes tumor metastasis in murine xenografts. In addition, it is shown that ZDHHC15 associates with Kidney and brain protein (KIBRA, also known as WW‐ and C2 domain‐containing protein 1, WWC1), an upstream component of Hippo signaling, and mediates its palmitoylation. KIBRA palmitoylation leads to its degradation and regulates its subcellular localization and activity toward the Hippo/YAP pathway. Moreover, PA enhances KIBRA palmitoylation and degradation. It is further shown that combinatorial targeting of YAP and fatty acid synthesis exhibits augmented effects against metastasis formation in mice fed with a Palm diet. Collectively, these findings uncover a ZDHHC15‐YAP feedback loop as a previously unrecognized mechanism underlying PA‐promoted tumor metastasis and support targeting YAP and fatty acid synthesis as potential therapeutic targets in PA‐driven tumor metastasis.

## Graphical abstract

This study uncovers that dietary palmitic acid (PA) acts as a potent inducer of YAP signaling in metastasis in breast and ovarian cancers, which is achieved by increasing the expression of palmitoyltransferase ZDHHC15, which in turn palmitoylates KIBRA and forms a feedback loop with YAP. Targeting the ZDHHC15‐YAP loop may be a therapeutic strategy for mitigating PA‐mediated tumor metastasis.

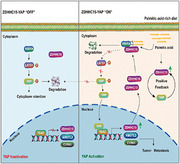

## 1 Introduction

High dietary fat intake is implicated in the incidence and development of many cancers. Palmitic acid (PA) is the most common saturated fatty acid in the human diet and serves as an energy source and building block for lipid metabolism. Mounting evidence demonstrates that PA is associated with cancer progression.^[^ ^1^, ^2^, ^3^, ^4^, ^5^ ^]^ Notably, there is growing evidence that long‐term health risks are associated with a diet rich in PA regarding metastatic progression in patients with some types of cancer.^[^ ^6^, ^7^, ^8^ ^]^ Dietary PA, but not oleic acid (OA) or linoleic acid, promotes metastasis in oral carcinoma and melanoma in mice by increasing FA transporter CD36.^[^ ^6^, ^9^ ^]^ Furthermore, dietary PA reprograms cancer cells through histone methylation to promote tumor innervation and metastasis formation,^[^ ^8^ ^]^ unraveling a novel mechanism involving PA‐enhanced metastasis in OSCC and melanoma. In breast cancer, a recent study demonstrated that PA is a key driver of tumor initiation in hormone receptor‐negative breast cancer in obesity.^[^ ^10^ ^]^ Additionally, PA has been identified as a molecular link between a high‐fat diet, prometastatic niche formation, and regulation of gene expression in breast cancer metastasis.^[^ ^11^ ^]^ In addition, the PA‐rich diet could induce increased accumulation of malignant ascites, the main vehicle of ovarian cancer metastasis,^[^ ^12^ ^]^ although the underlying mechanism remains to be investigated. Collectively, these studies highlight a prometastatic effect of PA in diverse cancer contexts. However, the effects of PA on cancer progression and metastasis vary between cancer types. Notably, a conflicting effect of PA, i.e., inhibiting or promoting tumor progression, has been observed in several types of cancer. In gastric cancer, it was reported that PA induces gastric cancer metastasis through CD36‐dependent activation of the AKT pathway.^[^ ^13^ ^]^ However, a recent study reported that PA inhibited the growth and metastasis of gastric cancer by blocking the STAT3 signaling pathway.^[^ ^14^ ^]^ Additionally, in prostate cancer, while early investigations suggested that higher PA intake is a risk factor for the incidence and progression of prostate cancer,^[^ ^15^, ^16^ ^]^ a recent report showed that PA inhibited prostate cancer cell proliferation and metastasis by suppressing the PI3K/Akt pathway.^[^ ^17^ ^]^ The conflicting effect of PA was also documented in hepatocellular carcinoma (HCC). On one hand, it was reported that PA impaired HCC development by modulating membrane fluidity and glucose metabolism;^[^ ^18^ ^]^ On the other hand, PA promoted HCC cell migration by inducing IRE1–XBP1–ZEB signaling.^[^ ^19^ ^]^ Another report showed that PA rewired lipid metabolism, regulating HCC progression in a PHF2/SREBP1c axis‐dependent manner.^[^ ^20^ ^]^ Despite multiple mechanisms underlying these conflicting results, there is currently no convincing and accepted explanation for the differential roles of PA in cancer progression. On one hand, PA not only serves as an energy source and building block for lipid metabolism for cancer growth but also acts as a signaling molecule in cancer cells to influence various signaling pathways. On the other hand, different cancers or specific cancer cells have diversely activated signaling pathways and/or different metabolic reprogramming. Collectively, considering that excess PA treatment or a diet rich in PA influences multiple signaling pathways in diverse contexts, the extensive elucidation of the molecular mechanisms of PA‐affected tumor progression in distinct tumors poses a formidable challenge.

The evolutionarily conserved Hippo signaling pathway plays a pivotal role in development and tumorigenesis.^[^ ^1^, ^2^, ^21^, ^22^, ^23^ ^]^ In mammals, the core components of the Hippo pathway consist of a kinase cassette comprising the mammalian sterile 20‐like 1/2 (MST1/2, also called STK4/3), large tumor suppressor homolog 1/2 (LATS1/2), and of their respective cofactors SAV1 and MOB1A/MOB1B, and downstream effectors Yes1‐associated transcriptional regulator (YAP1, also referred to as YAP) and WW domain–containing transcription regulator1 (WWTR1, also known as TAZ).^[^ ^3^, ^4^, ^5^, ^9^, ^21^, ^24^ ^]^ Phosphorylation by LATS1/2 inhibits the transcriptional coeffect or activity of YAP/WWTR1 through cytoplasmic sequestration and proteasomal degradation.^[^ ^25^ ^]^ On the contrary, inactivation of the upstream kinases MST1/2 and/or LATS1/2 leads to YAP and WWTR1 nuclear retention and binding to TEA/TEF‐domain transcription factors (TEAD1–4) to mediate target gene expression.^[^ ^6^, ^26^ ^]^ The activity of the MST‐LATS‐YAP core cassette is tightly controlled by an array of upstream regulators, these include Neurofibromin 2 (NF2, Merlin in Drosophila),^[^ ^7^, ^27^, ^28^ ^]^ Kidney and brain protein (KIBRA, also known as WW‐ and C2 domain–containing protein 1, WWC1),^[^ ^29^, ^30^, ^31^ ^]^ angiomotins (AMOT, AMOTL1, and AMOTL2),^[^ ^32^, ^33^ ^]^ PTPN14,^[^ ^34^ ^]^ MAP4Ks,^[^ ^35^ ^]^ STRIPAK phosphatase complex,^[^ ^36^ ^]^ and G‐protein‐coupled receptors (GPCRs).^[^ ^37^ ^]^ Accumulating evidence demonstrates that Hippo/YAP pathway is implicated in tumor metastasis.^[^ ^4^, ^38^, ^39^, ^40^ ^]^ However, most of the studies utilize the gain‐ or lost‐of function approach to explore the role of the components in the Hippo/YAP signaling in cancer development. In tumor metastasis, how the Hippo/YAP pathway is regulated by upstream signaling molecules, and most importantly which upstream signal is responsible for the activation of the Hippo/YAP pathway, remain not completely understood. A recent work revealed that YAP is selectively activated by bile acids in lymph node‐metastatic tumors, and functions as a crucial driver for tumor lymph node metastasis through selective stimulation of fatty acid oxidation (FAO).^[^ ^39^ ^]^ As PA is implicated in regulating the Hippo/YAP pathway,^[^ ^41^ ^]^ whether and how the Hippo/YAP pathway plays a role in PA‐promoted metastasis remains unknown.

Protein S‐palmitoylation (here after simply palmitoylation) attaches palmitate, a 16‐carbon saturated fatty acid, to the cysteine residue of a protein with a thioester linkage.^[^ ^42^, ^43^ ^]^ In mammalian cells, the majority of protein palmitoylation is catalyzed by the zinc finger DHHC‐type containing (ZDHHC) family of palmitoyltransferases (PATs).^[^ ^44^ ^]^ The human proteome encodes 23 ZDHHC proteins, characterized by the presence of an invariant Asp–His–His–Cys (DHHC) cysteine‐rich domain harboring the catalytic center of the enzyme. Palmitoylation has been found to modulate protein localization, stability, and function, and is implicated in cancer development.^[^ ^44^ ^]^ Recent studies have shown that palmitoylation is implicated in the regulation of Hippo signaling pathway.^[^ ^45^ ^]^ Remarkably, TEAD transcription factors possess intrinsic palmitoylating activities and undergo auto‐palmitoylation by direct binding to palmitoyl‐CoA,^[^ ^45^ ^]^ which is critical for TEADs’ stability and transcriptional activity.^[^ ^45^, ^46^ ^]^ Further study in immortalized breast epithelial cells revealed that palmitoylation of TEAD is regulated by cell density and/or NF2/merlin in a LATS‐independent manner.^[^ ^47^ ^]^ Importantly, a selective inhibitor targeting TEAD auto‐palmitoylation has shown antitumor effects in in vivo models.^[^ ^48^, ^49^ ^]^ However, the role and underlying mechanism of such palmitoylation in regulating Hippo pathway as well as cancer progression remain to be investigated.

Here, we report that dietary PA promotes metastasis in breast and ovarian cancers at least in part through YAP. We identified ZDHHC15 as an upstream regulator of the Hippo/YAP pathway in a palmitoyltransferase activity‐dependent manner, acting as a feedback regulator of YAP activity. Moreover, PA drives the ZDHHC15‐YAP loop, potentiating YAP activity and hence enhancing cancer metastasis. In addition, we show that ZDHHC15 interacts with KIBRA and mediates the palmitoylation of KIBRA. Notably, PA enhances KIBRA palmitoylation, which leads to its degradation, regulates its subcellular localization, and affects its activity toward the Hippo/YAP pathway. We also explore that pharmacological targeting of the ZDHHC15‐YAP loop as well as fatty acid synthesis inhibits metastasis formation in mice fed with a Palm diet. Collectively, our data provide novel insights into the underlying mechanisms by which PA promotes tumor metastasis, and suggest targeting the ZDHHC15‐YAP loop as a potential therapeutic strategy for mitigating PA‐mediated tumor metastasis.

## 2 Results

### 2.1 Palmitic Acid Promotes Cancer Metastasis Partially Through YAP Signaling

PA has been reported to promote tumor metastasis in certain cancer types.^[^ ^8^, ^12^, ^50^, ^51^ ^]^ To address whether dietary PA could exert a premetastatic effect in breast or ovarian cancer in vivo, firstly we performed tail‐vein injections with TOV‐112D ovarian cancer cell lines expressing luciferase in immunodeficient nude mice, which were fed with a high‐fat diet plus Palm oil (Palm) or Olive oil (Olive) (Figure S1A, Supporting Information, 10 mice were randomly divided into 2 groups, *n =* 5 mice in each group). ELISA detection revealed higher triglyceride in the serum of mice fed with a Palm‐diet than in mice fed with an Olive‐diet (Figure S1B, Supporting Information). The in vivo bioluminescence imaging revealed that mice in the Palm‐diet group exhibited more metastatic lesions in the lung (Figure S1C, Supporting Information), and shorter survival (Figure S1D, Supporting Information) than those in the Olive‐diet group. Histologic analyses of the pulmonary nodes with hematoxylin‐eosin (H&E) staining confirmed the metastasis to the lung (Figure S1E, Supporting Information). In addition, the Palm diet significantly increased the number of metastatic lung nodules (Figure S1F, Supporting Information), as well as the mice's body weights at 21–28 days after injection compared to the Olive‐diet group (Figure S1G, Supporting Information). Similar results were obtained in mice injected with MDA‐MB‐231 breast cancer cell lines expressing luciferase (Figure S1H–M, Supporting Information, 10 mice were randomly divided into 2 groups, *n =* 5 mice in each group). Moreover, consistent with the findings in a tail vein injection model, PA treatment significantly promoted the metastasis to lung in mice orthotopically implanted with MDA‐MB‐231 cells compared with treatment with olive oil (Figure S2A,B, Supporting Information, 10 mice were randomly divided into 2 groups, *n =* 5 mice in each group). H&E staining confirmed the metastasis to lung (Figure S2C, Supporting Information). Moreover, the number of metastatic lung nodules were significantly increased in the mice with a Palm‐diet compared to the Olive‐diet (Figure S2D, Supporting Information). Additionally, we observed an increase in the body weight of mice with a Palm‐diet at 21–28 days after injection (Figure S2E, Supporting Information). In addition, in an in vitro Matrigel invasion chamber assay, PA treatment significantly increased the invasion capacity of MDA‐MB‐231, HCC1954 breast cancer and TOV‐112D, POE‐4 ovarian cancer cell lines compared to BSA treatment (Figure S3A, Supporting Information). Fatty acid synthase (FASN) catalyzes the biosynthesis of palmitate.^[^ ^52^ ^]^ We also tested the effects of TVB‐3166, a selective FASN inhibitor.^[^ ^53^ ^]^ TVB‐3166 decreased the invasion capacity of HCC1954 and TOV‐112D cells (Figure S3B, Supporting Information). Supporting the relevance of PA to metastasis in our in vivo models, Gene Ontology (GO) and Kyoto Encyclopedia of Genes and Genomes (KEGG) analyses of RNA‐seq data performed in TOV‐112D cells, which revealed the differentially expressed genes in the absence or presence of PA (SRA accession number: PRJNA986045 and GSE279101; Table S1, Supporting Information), indicated that PA treatment significantly regulated extracellular matrix and substrate‐dependent cell migration (Figure S3C, Supporting Information, for Go analyses; Figure S3D, Supporting Information, for KEGG analyses). Altogether, these data support that a Palm‐rich diet promotes metastasis in the tested breast and ovarian cancer mouse models.

**Figure 1:**
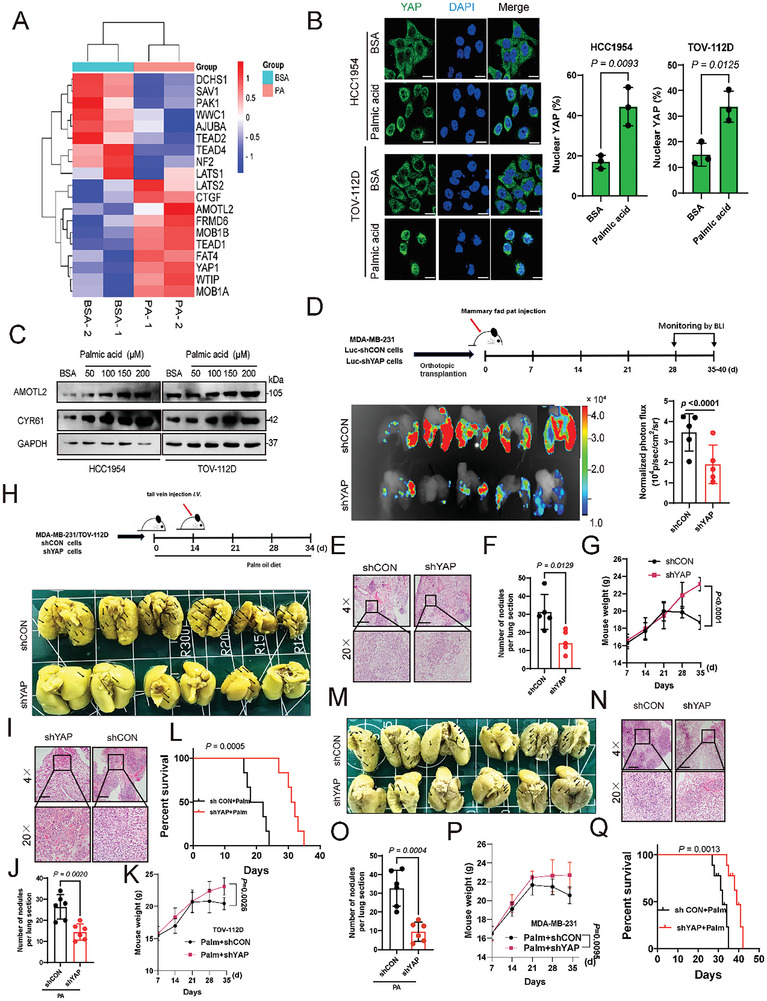
Palmitic acid promotes cancer metastasis in part through YAP signaling. A) GO analysis conducted on differentially expressed genes in TOV‐112D cells treated with palmitic acid (100 µm, 24 h) or BSA control. B) Immunofluorescence staining of YAP (green) on HCC1954 and TOV‐112D cells treated with palmitic acid of 100 µm for 24 h. Nuclear staining was achieved using DAPI (blue). Scale bars represent 25 µm. C) HCC1954 and TOV‐112D cells were treated with various concentrations of palmitic acid. Immunoblotting analysis was performed to examine the expression levels of AMOTL2 and CYR61. Representative results were obtained from at least three independent experiments with similar results. D) Experimental protocol for YAP‐depleted MDA‐MB‐231 cells to promote tumor metastasis in an orthotopic transplantation model. Shown are images and BLI quantification of lung metastasis (*n =* 5 mice in each group). E) Representative images of H&E staining (*n =* 5 mice in each group). F, G) The number of lung metastatic nodules (F) and G) weight was counted and statistically analyzed, (*n =* 5 mice in each group). H) Effect of YAP on PA‐promoted tumor metastasis potential in YAP‐knockdown TOV‐112D cells was examined by the tail vein injection metastasis model. After 5 weeks mice were euthanized, and the brightfield lung images of each group are shown (*n =* 6 for each group). I) Representative images of H&E staining (*n =* 6 for each group). J–L) The number of J) lung metastatic nodules, K) weight, and L) survival rate was counted and statistically analyzed (*n =* 6 for each group). M) Effect of YAP on PA‐promoted tumor metastasis potential in YAP‐knockdown MDA‐MB‐231 cells were examined by the tail vein injection metastasis model. After 5 weeks mice were euthanized, and the brightfield lung images of each group are shown (*n =* 6 for each group). N) Representative images of H&E staining (*n =* 6 for each group). O–Q) The number of O) lung metastatic nodules, P) weight, and Q) survival rate were counted and statistically analyzed. (*n =* 6 for each group). *P* values were determined by the two‐tailed Student's *t*‐test (B, D, F, J, O) two‐way analysis of variance analysis (G, K, P), and Log‐rank Mantel–Cox test (L, Q). Data are representative of three independent experiments.

We next sought to explore the underlying mechanism through which PA promotes metastasis in the tested cancers. Heatmap plot based on our RNA‐seq data performed in TOV‐112D cells showed that genes encoding the Hippo pathway components and YAP targets were significantly deregulated upon PA treatment (**Figure** **1**A), suggesting that PA could regulate Hippo/YAP signaling. PA has been shown as an upstream regulator of Hippo pathway in several studies.^[^ ^46^, ^54^, ^55^ ^]^ We observed that YAP nuclear retention in HCC1954 and TOV‐112D cells was enhanced upon PA treatment as monitored by both immunostaining (Figure 1B) and subcellular fractionation assay (Figure S4A, Supporting Information). In addition, PA treatment markedly increased the expression of *Amotl2* and *Cyr61*, two YAP target genes, in these cells (Figure 1C). Moreover, addition of TVB‐3166 led to a dose‐dependent decrease in both YAP levels and YAP target gene expression (Figure S4B, Supporting Information). Of note, short hairpin RNA (shRNA)‐mediated stable knockdown of YAP in these cells markedly abrogated the effect of PA on YAP target gene expression (Figure S4C, Supporting Information). Collectively, these data indicate that PA potentiates YAP signaling in the tested cancer cells. YAP signaling has been implicated in cancer metastasis.^[^ ^56^, ^57^ ^]^ We observed that YAP depletion led to a significant reduction in the invasion potential of HCC1954, MDA‐MB‐231, PEO‐4, and TOV‐112D cells in the transwell invasion assay (Figure S4D, Supporting Information). Furthermore, mice bearing orthotopic xenografts initiated from YAP‐depleted MDA‐MB‐231 cells had less pulmonary micrometastases than mice bearing the matched control cells (Figure 1D, 10 mice were randomly divided into 4 groups, *n =* 5 mice in each group). H&E staining confirmed the metastasis to lung (Figure 1E). Moreover, YAP depletion significantly decreased the number of metastatic lung nodules compared to control group (Figure 1F). Additionally, we observed a significant decrease in the body weight of mice in the control group at 21–35 days compared to YAP‐depletion (Figure 1G).

We next investigated whether YAP signaling plays a role in PA‐promoted metastasis. In the transwell invasion assay, we showed that YAP‐depleted HCC1954, MDA‐MB‐231, PEO‐4, and TOV‐112D cells displayed significantly less invasion potential upon PA treatment compared to their matched control cells (Figure S4E, Supporting Information).

To explore the signaling molecules downstream of the YAP signaling involving PA‐potentiated metastasis, we analyzed our RNA‐seq data of PA‐ and BSA‐treated TOV‐112D cells, and the Heatmap showed the differential expression of signaling molecules involved in cancer metastasis such as LIMD1, CTGF, TGFB2, CYR61, ITGAV, FAT4, ODC1 (Figure S4F, Supporting Information). Of note, these genes have been reported as YAP‐targeted genes and functioned in YAP‐mediated cancer metastasis.^[^ ^43^, ^58^, ^59^, ^60^, ^61^, ^62^ ^]^ Supporting the above analysis, both YAP knockdown by siRNA or treatment with YAP inhibitor TED‐347^[^ ^63^ ^]^ impaired PA‐enhanced expression of CYR61, ITGAV, LIMD1, and ODC1 in either HCC1954 or TOV‐112D cells (Figure S4G, Supporting Information). Furthermore, the in vivo effects of YAP in PA‐mediated metastasis were evaluated using our breast and ovarian cancer xenograft model, in which mice were injected with YAP‐depleted TOV‐112D (Figure 1H–K) and MDA‐MB‐231 (Figure 1M–Q) cells through the tail veins and fed with a high‐fat diet plus Palm. In each independent animal experiment, 6 mice were used in each group. YAP depletion significantly decreased lung metastasis (Figure 1H–J for TOV‐112D group; Figure 1M–O for MDA‐MB‐231 group), extended life span (Figure 1L for TOV‐112D group; and Figure 1Q for MDA‐MB‐231 group), and increased the mice's body weights (Figure 1K forTOV‐112D group; Figure 1P for MDA‐MB‐231D group) compared with respective controls. Collectively, these data suggest an essential role of YAP in PA‐mediated tumor metastasis in vivo.

### 2.2 The Palmitoyltransferase ZDHHC15 Acts as A Positive Regulator of YAP Activity

We next sought to define how PA affects YAP signaling in breast and ovarian cancers. PA (C16:0) is the major lipid incorporated into S‐palmitoylated proteins,^[^ ^43^ ^]^ and promotes protein palmitoylation.^[^ ^64^, ^65^ ^]^ Recent studies suggest that protein palmitoylation is implicated in the regulation of Hippo/YAP signaling pathway,^[^ ^64^, ^65^ ^]^ as well as in tumor development.^[^ ^43^ ^]^ To examine whether protein palmitoylation is involved in PA‐mediated YAP signaling and tumor cell invasion, we tested the effect of 2‐bromopalmitate (2‐BP), a general protein palmitoylation inhibitor.^[^ ^66^ ^]^ We showed that 2‐BP treatment decreased YAP nuclear retention and its target gene expression in both HCC1954 and TOV‐112D cells (Figure S5A,B). Notably, PA‐promoted YAP target gene expression as well as cell invasion in these cells were substantially attenuated by 2‐BP (Figure S5C,D, Supporting Information).

**Figure 2:**
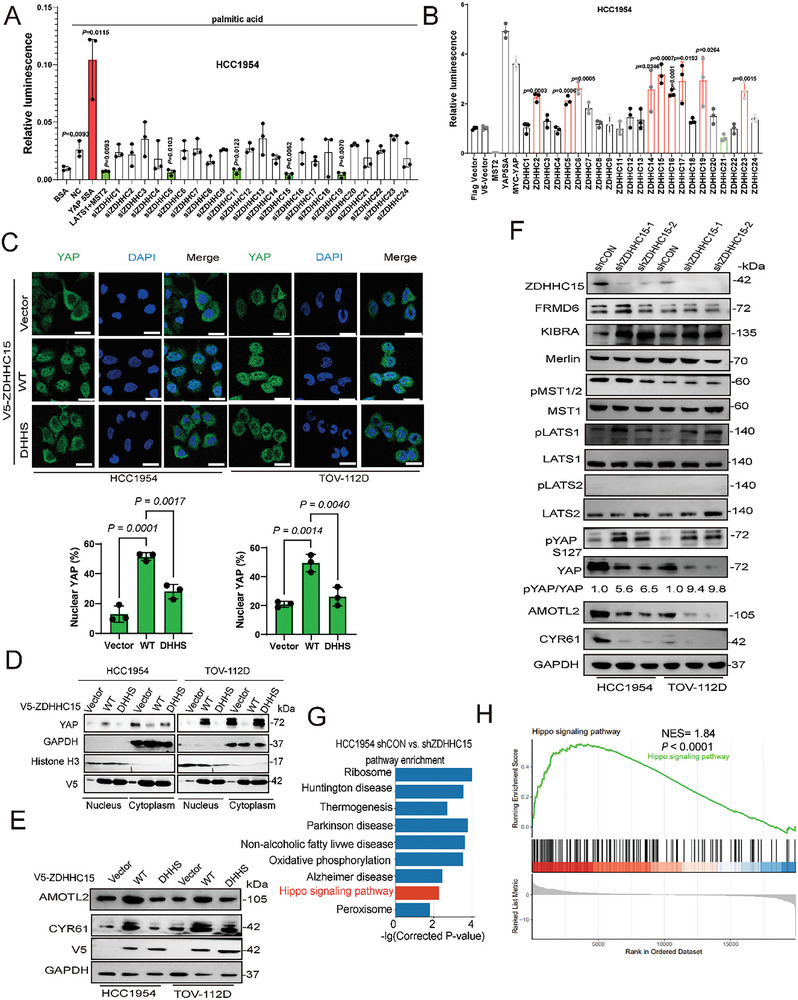
ZDHHC15 positively regulates YAP activity. A) TEAD luciferase reporter activity was assessed in HCC1954 cells following transfection with siRNAs targeting ZDHHC1‐24 respectively or nontargeting control (NC), or MST2/LATS1 or vector control, along with the 8xGTIIC‐luciferase reporter and pRL‐TK Renilla luciferase, with Renilla luciferase serving as an internal control. Cells were then treated with palmitic acid (PA, 100 µm) for 36 h. *P* values were determined using one‐way ANOVA with Dunnett's multiple comparison test (*n =* 3). B) HCC1954 cells following transfection with V5 or Flag–tagged‐ZDHHC1‐24 expression constructs, or YAP5SA or MST2/LATS1 or vector control, along with the 8xGTIIC‐luciferase reporter and pRL‐TK Renilla luciferase. Data were normalized to the vector control. *P* values were determined using one‐way ANOVA with Dunnett's multiple comparison test (*n =* 3) C) Immunofluorescence staining of YAP (green) and DAPI (blue) was performed on HCC1954 and TOV‐112D cells transfected with V5‐ZDHHC15 WT and DHHS variant. Scale bars: 25 µm. *P* values were determined using one‐way ANOVA with Dunnett's multiple comparison test (*n =* 3) D) Immunoblotting (IB) analysis of YAP in the nucleus and cytoplasm of TOV‐112D and HCC1954 cells transfected with V5‐ZDHHC15 WT and DHHS mutant constructs. E) IB analysis was conducted to evaluate the levels of AMOTL2 and CYR61 in HCC1954 and TOV‐112D cells following transfection with V5‐ZDHHC15 WT and DHHS variant. F) IB analysis of Hippo/YAP pathway in ZDHHC15‐depleted HCC1954 and TOV‐112D cells. G) KEGG pathway analysis of differential expressed mRNA transcripts in ZDHHC15‐depleted TOV‐112D cells and their respective control cells. H) Gene set enrichment analysis revealed enrichment in Hippo pathway. GAPDH was used as a loading control. Data are representative of three independent experiments.

In mammalian cells, the majority of protein palmitoylation is catalyzed by the 23 ZDHHC proteins.^[^ ^44^ ^]^ We next sought to identify the palmitoyltransferase(s) that could modulate YAP's transcription activity in cancer cells. To this purpose, we undertook both loss‐ and gain‐ of function screens that examined the effect of the ZDHHC acyltransferases on transcriptional outcome using a TEAD‐binding element driven‐luciferase reporter (8xGTIIC‐luciferase).^[^ ^67^ ^]^ MST2/LATS1, both upstream negative regulators of YAP activity, were utilized as a negative control, and a constitutively active YAP mutant YAP‐5SA (in which the serine residues phosphorylated by LATS1/2 were mutated to alanine residue) was used as a positive control.^[^ ^67^ ^]^ In the loss‐of‐function screen, we knocked down 23 ZDHHCs in HCC1954 and TOV‐112D cells using small interfering RNAs (siRNAs). The knockdown efficiency was examined by real‐time PCR (Figure S6A,B, Supporting Information). Silencing of ZDHHC11, ZDHHC15, or ZDHHC19 significantly abrogated PA‐induced TEAD‐luciferase transcriptional activity (**Figure** **2**A; Figure S6C, Supporting Information). In the gain‐of‐function screen, we overexpressed each of the 23 Flag‐tagged or V5‐tagged human ZDHHC palmitoyltransferases together with the TEAD‐luciferase constructs in HCC1954 and T47D breast cancer and PEO‐4 and TOV‐112D ovarian cancer cell lines and found that ZDHHC15 but not the other ZDHHCs, significantly enhanced TEAD transcriptional activity in the tested 4 cell lines (Figure 2B; Figures S6D and S7A, Supporting Information). In addition, using YAP subcellular localization as another readout for YAP's transcription activity, a significant increase in nuclear YAP localization upon ectopic expression of V5‐tagged ZDHHC15 (subsequently referred to as ZDHHC15‐WT), but not a palmitoyltransferase activity‐deficient ZDHHC15 mutant (in which the cysteine 159 in the DHHC motif was substituted to serine (subsequently referred to as ZDHHC15‐DHHS), thus losing the ability to transfer palmitate to its substrate), was visualized in the 4 tested cell lines by both immunofluorescence microscopy (Figure 2C; Figure S7B, Supporting Information) and subcellular fractionation analysis (Figure 2D; Figure S7C, Supporting Information). Accordingly, ectopic expression of V5‐tagged ZDHHC15‐WT but not the ZDHHC15‐DHHS mutant increased the expression of YAP target genes, *Amotl2* and *Cyr61* in the tested 4 cell lines (Figure 2E; Figure S7D, Supporting Information), suggesting that the palmitoyltransferase activity is required for ZDHHC15 to modulate YAP activity. In addition, YAP signaling and activity were assessed in HCC1954 and TOV‐112D cells transduced with shRNA targeting ZDHHC15 or control shRNA. Stable knockdown of ZDHHC15 in these cells led to an increase in pYAP/YAP ratio (Figure 2F), a decrease in nuclear YAP localization (Figure S7E, Supporting Information), and a reduction in YAP target gene expression (Figure 2F). In addition, ZDHHC15 knockdown led to an increase in pLATS1 levels in HCC1954 and TOV‐112D cells while the expression of several core components of Hippo pathway including MST1/2, and LATS1/2, remained largely unchanged (Figure 2F). In rescue experiments, ectopic expression of ZDHHC15‐WT, but not the ZDHHC15‐DHHS mutant, restored both YAP levels and YAP target gene expression in ZDHHC15‐depleted cells (Figure S7F, Supporting Information).

To further corroborate the role of ZDHHC15 in regulating YAP transcription activity in cancer cells, we performed RNA sequencing analyses in ZDHHC15‐depleted and control cell lines (GEO accession numbers: GSE235254 and GSE235258, Tables S2 and S3, Supporting Information). Both KEGG pathway and Gene set enrichment analysis (GSEA) analysis showed that the Hippo pathway was significantly enriched in both HCC1954 and TOV‐112D cells upon ZDHHC15 depletion (Figure 2G,H for HCC1954; Figure S7G,H, Supporting Information, for TOV‐112D). Furthermore, RT‐qPCR analysis confirmed the down‐regulation of several known YAP target genes such as *Amotl2, Axl, Ctgf*, and *Cyr61*, with a concomitant upregulation of several Hippo pathway upstream regulators *Frmd6, Ptpn14, Wwc1, Lats1*, and *Lats2* in ZDHHC15‐depleted HCC1954 and TOV‐112D cells (Figure S8A,B, Supporting Information). We next examined whether the deregulation of YAP target gene expression mediated by ZDHHC15 is via YAP. Indeed, either siRNA‐mediated knockdown of YAP (Figure S8C, Supporting Information) or treatment with a YAP/TEAD inhibitor TED‐347^[^ ^68^ ^]^ (Figure S8D, Supporting Information) robustly abrogated the increase in YAP target gene expression upon ectopic expression of ZDHHC15 in both HCC1954 and TOV‐112D cells. In addition, ectopic expression of YAP‐5SA, but not the YAPS94A mutant (in which the TEAD binding cite was mutated thus abolishing YAP's ability to activate TEADs),^[^ ^6^ ^]^ substantially reversed ZDHHC15 depletion‐triggered decrease in YAP target gene expression (Figure S8E, Supporting Information). Taken together, these findings strongly suggest that ZDHHC15 positively regulates YAP activity in cancer cells.

### 2.3 ZDHHC15 is a YAP‐Regulated Gene and Forms a Feedback Loop

**Figure 3:**
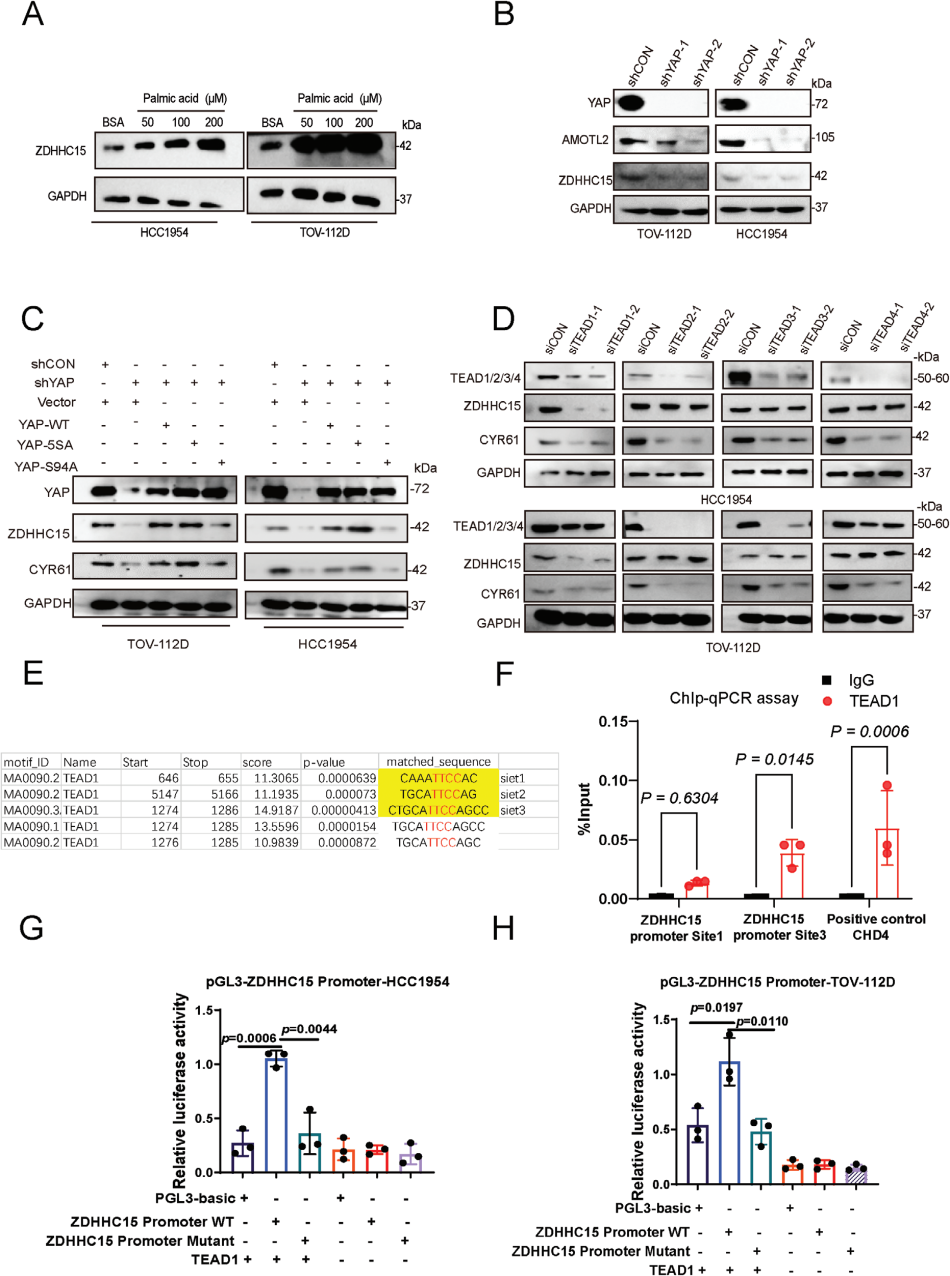
ZDHHC15 is a YAP‐regulated gene and forms a feedback loop. A) HCC1954 and TOV‐112D cells were treated with various concentrations of palmitic acid. Immunoblotting (IB) analysis was performed to examine the expression levels of ZDHHC15. B) IB analysis was performed using specific antibodies against YAP, ZDHHC15, and AMOTL2 in YAP‐depleted HCC1954 and TOV‐112D cells. C) HCC1954 and TOV‐112D cells with stable knockdown of YAP were transfected with either the vector control or plasmids encoding YAP‐WT, YAP‐5SA, and YAP‐S94A. The expression levels of YAP, ZDHHC15, and CYR61 were measured by IB. D) HCC1954 and TOV‐112D cells were transfected with non‐targeting siRNA control or respective siRNA targeting TEAD1, TEAD2, TEAD3, or TEAD4 for 48 h. IB analysis was performed for TEAD1/2/3/4, CYR61, and ZDHHC15 protein levels. GAPDH was used as a loading control. E) A schematic diagram illustrating the binding site sequence between TEAD1 and the ZDHHC15 promoter. F) Chromatin immunoprecipitation (ChIP) analysis demonstrated the binding of TEAD1 to the ZDHHC15 promoter in HCC1954 cells. Protein‐bound chromatin was immunoprecipitated with a TEAD1 antibody, with IgG serving as a control. The immunoprecipitated DNA was quantitatively analyzed using primers specific to the ZDHHC15 binding sequence, with CDH4 as a positive control. *P* values were determined by the two‐tailed Student's *t*‐test (*n =* 3). G,H) Luciferase reporter assays were performed using wild‐type or mutant ZDHHC15 promoter constructs in G) HCC1954 and H) TOV‐112D cells transfected with or without TEAD1. Data are presented as the mean ± SD. *P* values were assessed by one‐way ANOVA followed by Tukey's multiple‐comparison test (*n =* 3). Data are representative of three independent experiments.

Our RNA‐seq data performed in PA‐ or vehicle‐treated TOV‐112D cells indicated that the mRNA levels of ZDHHC15 were significantly upregulated upon PA treatment (Table S1, Supporting Information). Consistently, ZDHHC15 protein levels were markedly increased in PA‐treated HCC1954 and TOV‐112D cells (**Figure** **3**A), suggesting that ZDHHC15 might be a YAP‐regulated gene. To test this notion, we probed the expression of ZDHHC15 in YAP‐depleted cells. We found that YAP knockdown led to a pronounced reduction in ZDHHC15 expression in HCC1954 and TOV‐112D cells (Figure 3B). Notably, the decreased expression of ZDHHC15 induced by YAP depletion could be rescued by either YAP‐WT or YAP‐5SA, but not YAP‐S94A (Figure 3C), suggesting that YAP/TEAD transcription activity controls ZDHHC15 expression in these cells. Since YAP‐modulated gene expression is largely achieved via TEAD family (TEAD1/2/3/4)‐ mediated transcription, we next investigated which TEAD is responsible for ZDHHC15 expression. Knocking down TEAD1 but not other TEAD family members by individual siRNAs substantially downregulated the protein abundance of ZDHHC15 in HCC1954 and TOV‐112D cells (Figure 3D). In addition, the knockdown of TEAD1 in HCC1954 and TOV‐112D cells significantly downregulated the mRNA level of ZDHHC15 (Figure S9A, Supporting Information). Supporting these findings, we found a strong positive correlation between ZDHHC15 and TEAD1 expression in breast, liver, and ovarian cancers by mining the RNA‐sequencing data across different cancer types in The Cancer Genome Atlas (TCGA) (Figure S9B, Supporting Information). Thus, ZDHHC15 appeared to be transcriptionally regulated by TEAD1. To further test this notion, we located three putative TEAD1 binding sites on the promoter region of ZDHHC15 as predicted by the motif scan programmed FIMO (Find Individual Motif Occurrences) Version 5.4.1 in MEME suite using JASPAR (Figure 3E; Figure S9C, Supporting Information). Chromatin immunoprecipitation (ChIP)‐qPCR assays using an antibody against TEAD1 demonstrated that TEAD1 was enriched in the ZDHHC15 promoter at the third binding site in HCC1954 cells (Figure 3F). In addition, TEAD1 was enriched in CDH4 promoter (Figure 3F), which was used as a positive control.^[^ ^54^ ^]^ Moreover, luciferase reporter assays revealed that TEAD1 significantly enhanced the luciferase activity of the ZDHHC15 promoter containing the third putative wild‐type, but not a mutated TEAD1‐binding site in both HCC1954 and TOV‐112D cells (Figure 3G,H). Furthermore, overexpression of YAP5SA significantly increased the luciferase activity of the ZDHHC15 promoter in HCC1954 and TOV‐112D cells (Figure S9D,E, Supporting Information), whereas YAP silencing by siRNA or overexpressing YAPS94A could not (Figure S9D,E, Supporting Information). Collectively, these findings indicate that YAP/TEAD transcription activity controls ZDHHC15 expression. Given that ZDHHC15 promotes YAP activity in cancer cells as revealed by our above findings, our data suggest that ZDHHC15 functions as a positive feedback mechanism to enhance YAP activity.

### 2.4 ZDHHC15 Promotes Cancer Metastasis and is Associated with Poor Prognosis In Patients with Breast and Ovarian Cancers

**Figure 4:**
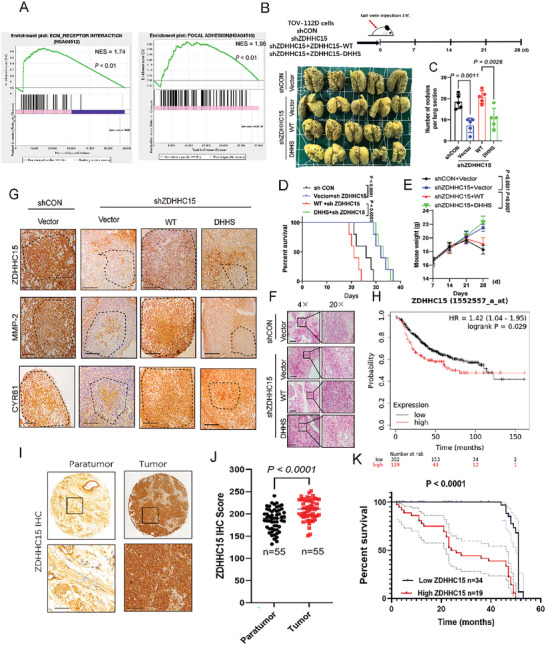
ZDHHC15 promotes metastasis in breast and ovarian cancers. A) RNA‐seq analysis of ZDHHC15‐depleted HCC1954 cells and control cells. Gene set enrichment analysis revealed enrichment in ECM receptor interaction and Focal adhesion. B) ZDHHC15‐depleted TOV‐112D cells were reconstituted with ZDHHC15‐WT or ZDHHC15‐DHHS constructs, then subjected to athymic nude mice xenograft through tail vein injection. After 5 weeks mice were euthanized, and the brightfield lung images of each group are shown (*n =* 5 mice in each group). C–E) The number of lung metastatic nodules (C), survival rate (D), and weight (E) were counted and statistically analyzed. (*n =* 5 mice in each group). F–G) Representative images of H&E staining (F) and immunohistochemical staining (G) of ZDHHC15, MMP‐2, and CYR61 performed on paraffin‐embedded xenograft tumor tissues. Scale bar: 100 µm. H) Kaplan–Meier survival analysis of grade III breast cancer samples from TCGA (*n =* 481, *P* = 0.029). I) Representative IHC staining of ZDHHC15 was performed on a cohort of breast cancer tissues. J) IHC score of ZDHHC15 for the panel. K) Kaplan–Meier analysis of the recurrence‐free survival of breast cancer patients in the cohort with high v.s. low ZDHHC15 expression. Data are presented as the mean ± SD. *P* values were determined by the two‐tailed Student's *t*‐test (J), the one‐way ANOVA, followed by Tukey's post hoc test (C), two‐way analysis of variance analysis (E), and Log‐rank Mantel–Cox test (D, K). Data are representative of three independent experiments. Scale bar: 100 µm. Data are representative of three independent experiments.

As we have shown that ZDHHC15 promotes YAP activity and forms a feedback loop with YAP in cancer cells, we next examined whether ZDHHC15 would influence tumor metastasis. GSEA analysis of RNA‐seq (GEO accession number: GSE235258) performed in HCC1954 cells with or without ZDHHC15 silencing indicated that ZDHHC15 could impact cancer cell migration/invasion (**Figure** **4**A). In line with the RNA‐seq data, knockdown of ZDHHC15 in HCC1954 or TOV‐112D cells resulted in a significant decrease in cell invasion potential (Figure S10A, Supporting Information) as well as MMP‐2/9 protein levels (Figure S10B, Supporting Information), which could be rescued by re‐expression of either ZDHHC15‐WT or YAP5SA, but not ZDHHC15‐DHHS mutant in these ZDHHC15‐knockdown cells (Figure S10A,B, Supporting Information). Notably, YAP knockdown‐induced downregulation of the cell invasive capacity in HCC1954 or TOV‐112D cells could not be rescued by re‐expression of ZDHHC15 in these cells (Figure S10C, Supporting Information), suggesting that ZDHHC15 promotes cancer cell invasion upstream of YAP. We next investigated the pro‐metastatic effects of ZDHHC15 in mouse models in which ZDHHC15‐depleted, ZDHHC15‐depleted TOV‐112D cells reintroduced with ZDHHC15 WT or its DHHS mutant, and control TOV‐112D cells were injected via tail veins (20 mice were randomly divided into 4 groups, *n =* 5 mice in each group). Brightfield lung imaging revealed that ZDHHC15 knockdown significantly reduced lung metastasis (Figure 4B), decreased the number of metastatic lung nodules (Figure 4C), and extended animal survival (Figure 4D) compared with the respective controls. We observed a significant increase in the body weight of mice in the ZDHHC15‐knockdown group (Figure 4E). The lung lesions in tumor sections were further confirmed by H&E staining (Figure 4F). Notably, these in vivo phenotypes by ZDHHC15 knockdown were greatly rescued by re‐expression of ZDHHC15‐WT but not ZDHHC15‐DHHS mutant (Figure 4B–F). Moreover, IHC analysis revealed reduced staining for ZDHHC15, MMP‐2, and CYR61in tumor sections from animals receiving ZDHHC15‐depleted tumor cells (Figure 4G). Notably, this staining pattern induced by ZDHHC15 depletion was markedly reversed by re‐expression of ZDHHC15‐WT but not ZDHHC15‐DHHS mutant (Figure 4G). In addition to the tail vein injection model, we orthotopically implanted control or ZDHHC15‐depleted MDA‐MB‐231 cell‐derived tumors in nude mice (Figure S11A, Supporting Information, 12 mice were randomly divided into 2 groups, *n =* 6 mice in each group). Brightfield lung imaging showed that ZDHHC15 knockdown significantly suppressed cell metastasis to lung as determined by the metastatic nodes in lungs (Figure S11B, Supporting Information), decreased the number of metastatic lung nodules (Figure S11C, Supporting Information), increased the body weight at 21–28 days after inoculation (Figure S11D, Supporting Information) compared with the respective controls. Additionally, a markedly decreased staining for ZDHHC15, CYR61, and MMP‐2 in the metastatic nodes were detected by H&E and IHC staining (Figure S11E, Supporting Information). We further explored the palmitoyltransferase activity of ZDHHC15 in cancer metastasis in mice injected with MDA‐MB‐231 cells overexpressing ZDHHC15 WT or ZDHHC15 DHHS mutant and control cells (Figure S11F, Supporting Information, 15 mice were randomly divided into 3 groups, *n =* 5 mice in each group). By performing brightfield lung imaging (Figure S11G, Supporting Information), H&E and IHC staining (Figure S11H,I, Supporting Information), determining body weight (Figure S11J, Supporting Information), counting the number of lung metastatic lesions (Figure S11K, Supporting Information), and calculating the survival rate (Figure S11L, Supporting Information), we showed that compared to ZDHHC15 WT, the DHHS mutant lost its ability to promote cancer metastasis. Together, our data indicate that ZDHHC15 expression and its palmitoyltransferase activity are critical for metastasis in breast and ovarian cancers. In addition, consistent with our in vitro YAP knockdown results, IHC staining of pulmonary micrometastases samples from mice bearing orthotopic xenografts derived from YAP‐depleted TOV‐112D cells revealed decreased staining for ZDHHC15, CYR61, and MMP‐2 (Figure S11M, Supporting Information). Collectively, these data suggest that the ZDHHC15‐YAP loop is functional in cancer metastasis.

To assess the clinical significance of ZDHHC15 in cancer development, we evaluated the overall survival of patients with breast or ovarian cancers in relation to ZDHHC15 expression in The Cancer Genome Atlas (TCGA) datasets. Kaplan‐Meier survival analyses showed that elevated ZDHHC15 mRNA levels significantly correlated with worse survival in patients with either breast cancer (Figure 4H, *n =* 481; grade III; *P* = 0.029) or ovarian cancer (Figure S12A, Supporting Information *n =* 614; *P* = 0.003), suggesting that ZDHHC15 is a significant prognostic factor in breast and ovarian cancers. In addition, by mining the TIMER (Tumor Immune Estimation Resource) database (http://timer.comp‐genomics.org/), we found that ZDHHC15 expression was positively correlated with YAP expression level in patients with breast cancer (Figure S12B, Supporting Information, *R* = 0.161, *p* < 0.05). To further corroborate the clinical relevance of ZDHHC15 expression in breast cancer, we probed ZDHHC15 protein expression in tumor samples from patients with breast cancer (*n =* 55). Clinicopathologic parameters of the patients are shown in Table S4 (Supporting Information). IHC analyses revealed a significantly increased staining for ZDHHC15 in tumor tissues relative to their paired non‐tumor tissues (Figure 4I,J). In addition, IHC staining showed that increased ZDHHC15 expression significantly correlated with both tumor grade (Figure S12C, lower panel shows the quantification result) and distal metastasis (Figure S12D,E, Supporting Information). Moreover, survival analysis showed that the patients with high ZDHHC15 expression (*n =* 34) showed poorer survival compared with those patients with low ZDHHC15 expression (*n =* 19) (Figure 4K). In addition, we analyzed ZDHHC15 expression in ovarian cancer of different grades in a cohort of 65 patients. The clinicopathologic parameters of the patients are shown in Table S5 (Supporting Information). IHC staining revealed that high expression of ZDHHC15 was associated with high‐grade ovarian cancer (Figure S12F,G, Supporting Information), and significantly correlated with distal metastasis (Figure S12H,I, Supporting Information). A significantly positive correlation between ZDHHC15 and YAP expression was noted in tissue samples from ovarian cancer patients (Figure S12J, Supporting Information).

To further investigate the clinical relevance of ZDHHC15 in cancer, we queried whole‐exome sequencing data of TCGA samples, which revealed several mutations in ZDHHC15, in individuals with either breast cancer or ovarian cancer (Figure S13A, Supporting Information). We found that both R240K and N260T mutations in ZDHHC15 led to a significant increase in TEAD luciferase activity as well as YAP target gene expression in HCC1954 cells (Figure S13B,C, Supporting Information), while the P306A mutant induced similar effects in TOV‐112D cells (Figure S13B,C, Supporting Information).

### 2.5 PA Hijacks the ZDHHC15‐YAP Loop Promoting Cancer Metastasis

**Figure 5:**
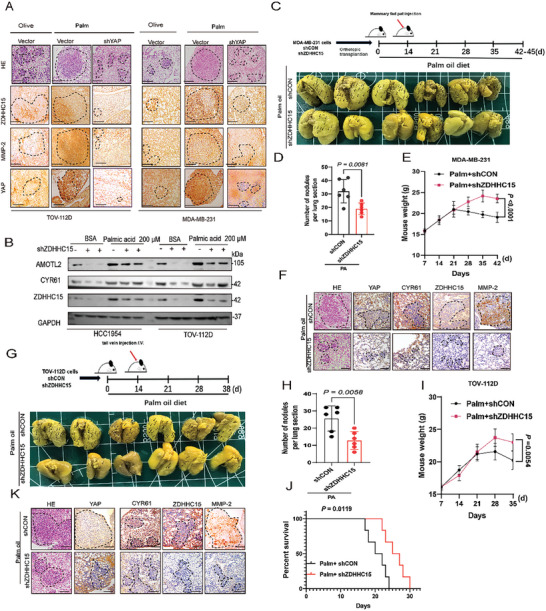
PA hijacks the ZDHHC15‐YAP loop promoting cancer metastasis. A) Immunohistochemical (IHC) staining of ZDHHC15, MMP‐2, and YAP in paraffin‐embedded xenograft tumor tissues from the indicated groups. Scale bar: 100 µm B) ZDHHC15‐depleted HCC1954 and TOV‐112D cells were stimulated with palmitic acid (200 µm) for 12 h. Immunoblotting (IB) analysis of the expression levels of AMOTL2, CYR61, and ZDHHC15. C) Treatment plan for ZDHHC15‐depleted MDA‐MB‐231 cells, mice were fed with a high‐fat diet plus Palm oil in an orthotopic transplantation model. After 6 weeks mice were euthanized, and the brightfield lung images of each group are shown (*n =* 6 mice in each group). D) The number of lung metastatic nodules and E) weight were counted and statistically analyzed. (*n =* 6 mice in each group). F) H&E and IHC staining of ZDHHC15, MMP‐2, CYR61, and YAP in paraffin‐embedded xenograft tumor tissues from the indicated groups (*n =* 6 mice in each group). Scale bar: 100 µm. G) Treatment plan for ZDHHC15‐depleted TOV‐112D cells, mice were fed with a high‐fat diet plus Palm oil in a tail vein injection model. The brightfield lung images of each group are shown (*n =* 6 mice in each group). (H‐J) The number of H) lung metastatic nodules, I) weight, and J) survival rate were counted and statistically analyzed. (*n =* 6 mice in each group). K) H&E and IHC staining of ZDHHC15, MMP‐2, CYR61, and YAP in paraffin‐embedded xenograft tumor tissues from the indicated groups (*n =* 6 mice in each group). Scale bar: 100 µm. *P* values were determined by the two‐tailed Student's *t*‐test (D, H), two‐way analysis of variance analysis (E, I), and Log‐rank Mantel–Cox test (J). Data are representative of three independent experiments.

We next sought to address whether the ZDHHC15‐YAP loop plays a role in PA‐promoted cancer metastasis. We have shown that PA treatment promoted cancer metastasis in both tail‐vein and orthotopic injection xenograft models (Figures S1C,I and S2B, Supporting Information). Furthermore, in each model, IHC staining of tumor sections from mice fed with a PA diet had increased staining for MMP‐2, YAP, and ZDHHC15 compared to mice fed with an Olive‐diet (**Figure** **5**A; Figure S14A, Supporting Information). We further performed IHC analysis of tumor sections of mice bearing YAP‐depleted cells or control cells upon PA treatment and found that YAP depletion led to a markedly decreased staining for ZDHHC15 (Figure 5A). We next examined the role of ZDHHC15 in PA‐mediated cancer metastasis. PA‐induced YAP target gene expression and cancer cell invasion were severely blunted in ZDHHC15‐depleted HCC1954 and TOV‐112D cells compared to their matched control cells (Figure 5B; Figure S14B, Supporting Information), and these effects upon ZDHHC15 depletion could be rescued by ZDHHC15‐WT but not the ZDHHC15‐DHHS mutant (Figure S14B, Supporting Information), indicating that ZDHHC15 abundance and its palmitoyltransferase activity are essential for PA‐mediated regulation of YAP activity in vitro. In addition, YAP5SA but not YAP S94A could largely rescue these phenotypes upon ZDHHC15 depletion in the presence of PA (Figure S14B, Supporting Information), suggesting that ZDHHC15 functions in PA‐promoted cancer cell metastasis via YAP. As we have shown that the FASN inhibitor TVB‐3166 decreased the invasion capacity of these cells (Figure S3B, Supporting Information), we next examined whether TVB‐3166 and C75^[^ ^69^ ^]^ would affect the ZDHHC15‐YAP signaling activated by PA. We found that TVB‐3166 markedly impaired PA‐induced increase in the protein levels of YAP and ZDHHC15 as well as YAP target genes AMOTL2 and CYR61 in both HCC1954 and TOV‐112D cells (Figure S14C, Supporting Information). To ascertain these in vitro findings, mice bearing orthotopic xenografts derived from ZDHHC15‐depleted or control MDA‐MB‐231 cells were fed with a Palm‐diet. (Figure 5C, 12 mice were randomly divided into 2 groups, *n =* 6 mice in each group) Brightfield lung imaging showed that compared to nontargeting shRNA controls, ZDHHC15 depletion led to less pulmonary micrometastases (Figure 5C), a reduction in number of metastatic lung lesions ((Figure 5D), and an increase in body weight at days 21–35 days after inoculation (Figure 5E). H&E and IHC staining revealed decreased staining for CYR61, MMP‐2, and YAP in tumor sections from mice bearing the ZDHHC15‐depleted cells (Figure 5F). Similar results were obtained in mice bearing tail‐vein‐injected xenografts derived from ZDHHC15‐depleted or control TOV‐112D cells when fed with a Palm diet (Figure 5G–J).

### 2.6 ZDHHC15 Complexes with KIBRA and Mediates its Palmitoylation

**Figure 6:**
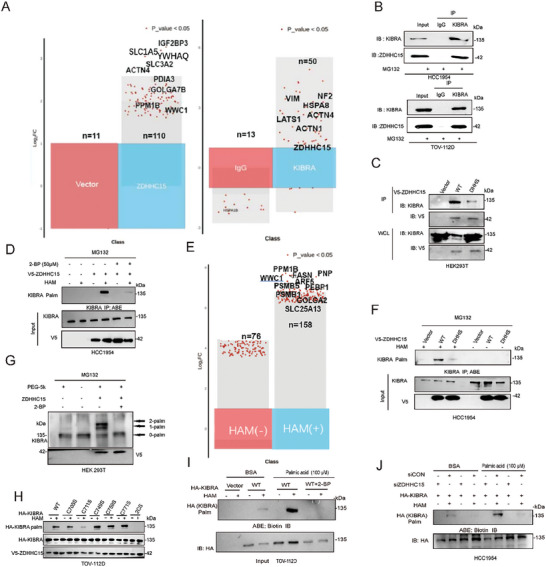
ZDHHC15 interacts with KIBRA and mediates its palmitoylation. A) Immunoprecipitation‐mass spectrometry (IP‐MS) was performed to identify ZDHHC15 or KIBRA binding partners in HCC1954 cells. The volcano plot of multiple group differences shows the same proteins in the BioGRID database that interact with ZDHHC15 (right) or KIBRA (left) as in this mass spectrometry result. B) HCC1954 and TOV‐112D cells were treated with MG132 (15 µm, 4 h) and IP was conducted using anti‐KIBRA or IgG antibodies, followed by immunoblotting (IB) with specific antibodies. C) IP for V5‐ZDHHC15 WT and DHHS mutant association with KIBRA in HEK‐293T cells. D) KIBRA palmitoylation levels were evaluated in HCC1954 cells transfected with V5‐ZDHHC15 using ABE assays, with or without 50 µm of 2‐BP for 24 h treatment in the presence or absence of HAM. E) HCC1954 cells transfected with V5‐ZDHHC15 were subjected to ABE analysis using V5‐tag antibodies in the presence or absence of HAM. The HAM condition served as a negative control. Streptavidin beads were utilized to enrich biotinylated proteins, which were subsequently identified through mass spectrometry (MS). Candidate proteins were considered when their abundance was at least two‐fold higher compared to the control in the HAM+ sample. The volcano plot with multiple group differences shows the same gene as the ZDHHC15 palmitoylation substrate in the SwissPalm database and Ocasio et al.^[^ ^104^ ^]^ as reported in this mass spectrometry result. F) ABE analysis was performed to assess KIBRA palmitoylation levels in HCC19431954 cells expressing ZDHHC15 WT and DHHS variant, with MG132 pretreatment. G) KIBRA palmitoylation were analyzed in HEK293T cells using APE assays upon ectopic expression of V5‐ZDHHC15 and/or 2‐BP (50 µm) treatment. H) ABE assay was conducted to analyze KIBRA palmitoylation in TOV‐112D cells ectopically overexpressing HA‐tagged‐KIBRA WT or mutant. I) TOV‐112D cells ectopically expressing HA‐KIBRA or vector control were treated with 100 µm palmitic acid or BSA, in the presence or absence of 2‐BP (50 µm) for 24 h prior to the ABE assay. J) ABE analysis of the palmitoylation of exogenous KIBRA in ZDHHC15‐depleted HCC1954 cells. The cells were transfected with HA‐KIBRA and treated with palmitic acid (PA, 100 µm) for 24 h.

We next sought to dissect the molecular mechanisms by which ZDHHC15 regulates YAP activity. To this end, we performed co‐immunoprecipitation (IP) experiments to identify possible ZDHHC15 interactors. V5‐tagged ZDHHC15 which was expressed in HCC1954 cells was immunoprecipitated and subjected to liquid chromatography‐tandem mass spectrometry (LC‐MS/MS) analysis. KIBRA, an upstream regulator of Hippo signaling, was among the candidates binding to ZDHHC15 (**Figure** **6**A, left panel; Table S6, Supporting Information). Notably, ZDHHC15 was identified as a binding partner of endogenous KIBRA in HCC1954 cells as revealed by IP‐MS analysis (Figure 6A, right panel; Table S7, Supporting Information), which also revealed some known KIBRA interactors such as NF2 and AMOT. We validated the interaction between KIBRA and ZDHHC15 at the endogenous level in HCC1954 and TOV‐112D cells (Figure 6B). In addition, we detected an interaction between V5‐tagged ZDHHC15 with HA‐tagged KIBRA, but not other key components of Hippo signaling, such as NF2/Merlin, MST1/2, LATS1/2, and YAP/TAZ which were tagged with Flag, HA, or Myc respectively in transfected HEK293T cells (Figure S15A, Supporting Information). Moreover, the GST‐pulldown assay suggested a direct interaction between ZDHHC15 and KIBRA (Figure S15B, Supporting Information). In addition, co‐IP experiments showed that the ZDHHC15‐DHHS mutant substantially lost the ability to complex with endogenous KIBRA (Figure 6C), indicating that the palmitoyltransferase activity is required for ZDHHC15 to form a complex with KIBRA. Furthermore, in co‐IP experiments, we determined that a region encompassing the DHHC motif (a.a. 137–199) in ZDHHC15, and the C2 domain (a.a. 658–785) in KIBRA are required for their interaction (Figure S15C,D, Supporting Information). GST pulldown results confirmed the C2 domain in KIBRA associated with ZDHHC15 (Figure S15E, Supporting Information).

As we have shown that the palmitoyltransferase activity is required for ZDHHC15 to associate with KIBRA and to regulate YAP activity, we speculated that ZDHHC15 could mediate KIBRA palmitoylation, thereby modulating KIBRA's function in the Hippo pathway. To detect KIBRA palmitoylation, we performed an in vitro acyl‐biotin exchange (ABE) assay on HCC1954 cells. The ABE approach removes palmitate from cysteine residues with hydroxylamine followed by substitution with biotin.^[^ ^70^ ^]^ In the presence of hydroxylamine, KIBRA was detected in the streptavidin fraction compared to the negative control without hydroxylamine, demonstrating the presence of palmitoylated KIBRA (Figure 6D). Treatment with 2‐BP, a general protein palmitoylation inhibitor, effectively diminished KIBRA palmitoylation, providing additional evidence for the specificity of the ABE assay (Figure 6D). Notably, in HEK293T cells without detectable ZDHHC15 expression,^[^ ^71^ ^]^ palmitoylation of endogenous KIBRA was not detected (Figure S16A, Supporting Information). However, ectopic expression of ZDHHC15‐WT in HEK293T cells markedly triggered the palmitoylation of endogenous KIBRA (Figure S16B, Supporting Information), which could be blunted by 2‐BP treatment.

In addition, we performed IP‐LC‐MS/MS analysis in ZDHHC15‐overexpressing HCC1954 cells to identify proteins that might be palmitoylated by ZDHHC15^[^ ^72^ ^]^ (Table S8, Supporting Information). KIBRA was identified as a palmitoylated candidate (Figure 6E), while palmitoylation of other key components in Hippo pathway such as NF2/Merlin, MST1/2, LATS1/2, and YAP/TAZ were not detected. Supporting the above findings, overexpression of ZDHHC15‐WT, but not ZDHHC15‐DHHS mutant, markedly enhanced endogenous KIBRA palmitoylation in HCC1954 cells (Figure 6F). In addition, the knockdown of ZDHHC15 dramatically reduced endogenous KIBRA palmitoylation in HCC1954 or TOV‐112D cells (Figure S16C, Supporting Information), suggesting that ZDHHC15 is required for KIBRA palmitoylation. Importantly, in line with the crucial role of C2 domain mediating KIBRA interaction with ZDHHC15, deletion of C2 domain, but not of other domains in KIBRA, largely abolished the palmitoylation of HA‐tagged KIBRA in transfected TOV‐112D cells (Figure S16D, Supporting Information), indicating that the C2 domain in KIBRA is essential for KIBRA palmitoylation by ZDHHC15.

We next sought to identify the potential palmitoylated site(s) in KIBRA in the presence of ZDHHC15. To this end, we performed an acyl‐PEGyl exchange gel shift (APEGS) assay that involves substituting acyl groups attached to Cys residues with a 5 kDa PEG mass tag, thus detecting one or more sites of S‐palmitoylation with a given substrate.^[^ ^73^, ^74^ ^]^ We found that ectopic expression of ZDHHC15 in HEK293T cells led to two up‐shifted bands representing mono‐ and di‐palmitoylated species of endogenous KIBRA in HEK293T cells (Figure 6G), indicating at least two sites of palmitoylation in KIBRA. Analysis of the palmitoylation of endogenous KIBRA in HCC1954 and TOV‐112D cells confirmed the two palmitoylated species of KIBRA upon ZDHHC15‐WT overexpression (Figure S16E, Supporting Information). Notably, 2‐BP treatment substantially abrogated the two palmitoylated species of endogenous KIBRA induced by ZDHHC15 in HCC1954 and TOV‐112D cells (Figure S16E, Supporting Information).

In addition, ectopic expression of several Golgi‐localized ZDHHCs including ZDHHC3, ZDHHC7, ZDHHC9, or ZDHHC2 which share high sequence identity with ZDHHC15, did not significantly induce the two palmitoylated species of KIBRA as ZDHHC15 did in HEK293T cells (Figure S16F, Supporting Information), supporting ZDHHC15 as a major palmitoyltransferase mediating the palmitoylation of KIBRA. In addition, similar to 2‐BP treatment, ectopic expression of the ZDHHC15‐DHHS mutant disrupted the two palmitoylated species of endogenous KIBRA induced by overexpression of ZDHHC15 in TOV‐112D cells (Figure S16G, Supporting Information). Given the critical role of C2 domain in KIBRA palmitoylation, we inferred that the palmitoylated cysteine residues are located within the C2 domain. We mutated all five cysteine residues C705, C711, C749, C759, and C771 on C2 domain to serine, and their palmitoylation levels were determined by using the ABE assay. Compared to KIBRA‐WT, a single mutation of either C705 or C711 (referred as C705S or C711S) yielded reduced palmitoylation signals while mutation of other 3 cysteine residues appeared no appreciable changes in TOV‐112D cells (Figure 6H). Consistently, simultaneous mutation of C705 and C711 (referred as KIBRA‐2CS) markedly abolished palmitoylation of KIBRA (Figure 6H). We further validated the absence of palmitoylation of the KIBRA‐2CS mutant in an APEGS assay. Compared to KIBRA‐WT (two up‐shifted forms), either the C705S or the C711S mutant displayed one up‐shift form while the KIBRA‐2CS mutant displayed no electrophoretic shifts in transfected HCC1954 cells (Figure S16H, Supporting Information), indicating that both C705 and C711 on C2 domain are apparently required for full palmitoylation of KIBRA. We noted that both C705 and C711 are highly conserved among different species (Figure S16I, Supporting Information). Taken together, these findings demonstrate that ZDHHC15 mediates KIBRA palmitoylation. It should be noteworthy that forced expression of ZDHHC15 did not induce appreciable palmitoylation of exogenous NF2/Merlin, MST1/2, SAV1, LATS1/2, MOB1, and YAP/TAZ in transfected TOV‐112D cells (Figure S17A–E, Supporting Information).

We next sought to address whether ZDHHC15‐mediated KIBRA palmitoylation is physiologically or pathological relevant. PA is the major lipid incorporated into S‐palmitoylated proteins,^[^ ^43^ ^]^ and promotes protein palmitoylation.^[^ ^64^, ^65^ ^]^ ABE assay detected enhanced palmitoylation of ectopically expressed KIBRA in response to PA, which was abrogated by 2‐BP (Figure 6I), whereas the palmitoylation of either the KIBRA‐2CS mutant or a C2 domain deletion construct of KIBRA was not affected by PA exposure (Figure S18A, Supporting Information). As FASN catalyzes the biosynthesis of palmitate.^[^ ^52^ ^]^ We also tested the effect of TVB‐3166, which targets FASN, on KIBRA palmitoylation. APGES approach confirmed that PA increased whereas TVB‐3166 decreased the palmitoylation of endogenous KIBRA (Figure S18B, Supporting Information). Remarkably, loss of palmitoylation of exogenous KIBRA upon ZDHHC15 knockdown could not be restored by PA treatment (Figure 6J), supporting further a crucial role of ZDHHC15 in mediating KIBRA palmitoylation. Of interest, palmitoylation of ZDHHC15 itself was enhanced by PA and decreased by TVB‐3166 respectively, as detected by the APGES approach (Figure S18C, Supporting Information).

### 2.7 KIBRA Palmitoylation Reduces its Protein Stability

**Figure 7:**
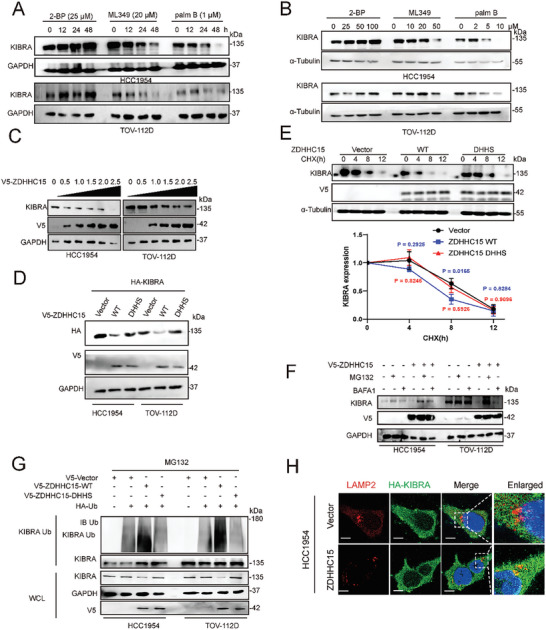
KIBRA palmitoylation decreases its protein stability. A,B) HCC1954 and TOV‐112D cells were treated with 2‐BP (25 µm), ML349 (20 µm), or palmB (1 µm) for the indicated times (A), or stimulated with indicated concentrations of 2‐BP, ML349, and palmB for 16 h B). The protein levels of KIBRA and GAPDH were analyzed by immunoblotting (IB). C) Ectopic expression of V5‐ZDHHC15 in HCC1954 and TOV‐112D cells. The protein levels of KIBRA were analyzed by IB. D) Ectopic expression of V5‐ZDHHC15 WT or DHHS mutant, along with HA‐KIBRA in HCC1954 and TOV‐112D cells. The protein level of HA‐KIBRA was analyzed by IB. E) Ectopic expression of V5‐ZDHHC15 was performed in HEK‐293T cells, followed by treatment with CHX (50 mg mL^−1^) for indicated time points. The endogenous KIBRA protein levels were analyzed by IB. *P* values were assessed by one‐way ANOVA followed by Tukey's multiple‐comparison test (*n =* 3). F) Ectopic expression of V5‐ZDHHC15 was performed in HCC1954 and TOV‐112D cells followed by treatment with bafilomycin A1 or MG132 for 4 h. The protein expression of KIBRA was analyzed by IB. G) HCC1954 and TOV‐112D cells were co‐transfected with V5‐ZDHHC15 WT or DHHS mutant, along with HA‐Ub constructs. After 48 h, cells were treated with MG132 (20 µm) for 4 h. IP was performed using anti‐KIBRA antibodies, followed by IB with the indicated antibodies. H) ZDHHC15‐overexpressed HCC1954 cells were stained with LAMP2 (red) and HA‐KIBRA (green). The colocalization of exogenous HA‐KIBRA and LAMP2 was examined using immunofluorescence microscopy. The cell nucleus (blue) was stained with DAPI. Scale bars: 25 µm. Data are representative of three independent experiments.

Palmitoylation of a variety of substrates has previously been shown to affect protein stability.^[^ ^43^, ^44^ ^]^ To determine whether KIBRA palmitoylation influences its protein stability, we treated HCC1954 and TOV‐112D cells with either the palmitoylation inhibitor 2‐BP or the depalmitoylation inhibitor ML349 or palm B. 2‐BP treatment increased, while exposure to ML349 or palm B decreased, KIBRA protein abundance, in both does‐ and time‐dependent manners (**Figure** **7**A,B), indicating palmitoylation modulates KIBRA protein accumulation. Of note, these compounds did not induce significant changes in mRNA levels of either KIBRA or ZDHHC15 (Figure S19A, Supporting Information). Given that ZDHHC15 mediates KIBRA palmitoylation, we suspected that ZDHHC15 could regulate KIBRA protein stability. Indeed, ectopically overexpression of ZDHHC15‐WT reduced the protein level of KIBRA in a dose‐dependent manner (Figure 7C), whereas knockdown of ZDHHC15 significantly increased KIBRA abundance (Figure 2F). Remarkably, overexpression of the ZDHHC15‐DHHS mutant failed to decrease exogenous KIBRA abundance as ZDHHC15‐WT did (Figure 7D). Notably, compared to KIBRA‐WT, the KIBRA‐2CS mutant appeared to be resistant to ZDHHC15‐mediated degradation (Figure S19B, Supporting Information), suggesting that the decrease in KIBRA protein abundance upon ZDHHC15 overexpression depends mainly on the palmitoylation of KIBRA itself. To determine whether the reduction in KIBRA protein levels by ZDHHC15 was the result of reduced stability or synthesis, cells were treated with cycloheximide (CHX) to block protein neo‐synthesis. Ectopically overexpression of ZDHHC15‐WT, but not the DHHS mutant, significantly increased the turnover rate of KIBRA in HEK293T cells (Figure 7E). The CHX‐chase assay also revealed that 2‐BP treatment stabilized KIBRA protein whereas Palm B substantially accelerated KIBRA degradation (Figure S19C, Supporting Information). Furthermore, ZDHHC15‐mediated degradation of KIBRA could be rescued partially by either the proteasomal inhibitor MG132 or the lysosomal inhibitor bafilomycin A1 (BAFA1) (Figure 7F) or 2‐BP (Figure S19D, Supporting Information), indicating that at least a fraction of palmitoylated KIBRA undergoes both proteasomal and lysosomal degradation. In support of this notion, both the endogenous KIBRA ubiquitination signal in HCC1954 and TOV‐112D cells (Figure 7G) or colocalization of ectopically expressed KIBRA with lysosome‐associated membrane protein 2 (LAMP2) in HCC1954 cells (Figure 7H) were markedly enhanced by ectopic expression of ZDHHC15‐WT, but not ZDHHC15‐DHHS mutant. Moreover, compared to KIBRA‐WT, KIBRA‐2CS mutant ubiquitination and its interaction with LAMP2 were markedly reduced even in the presence of ectopic ZDHHC15‐WT (Figure S19E,F, Supporting Information). In addition, a significantly reverse correlation between ZDHHC15 and KIBRA/WWC1 expression in ovarian cancer was revealed by using Gene Expression Profiling and Interactive Analysis (GEPIA, http://gepia.cancer‐pku.cn) (Figure S19G, Supporting Information, *R* = −0.32, *p* < 0.05). Furthermore, IHC analysis revealed a markedly increased staining for KIBRA in tumor sections from animals receiving breast and ovarian cancer cells with ZDHHC15 silencing (Figure S19H, Supporting Information). In addition, a significantly reverse correlation between ZDHHC15 and KIBRA expression was noted in tissue samples from breast cancer patients (Figure S19I, Supporting Information).

We next examined whether PA exhibits an effect on KIBRA protein abundance. Intriguingly, PA treatment induced a reduction in KIBRA abundance in both HCC1954 and TOV‐112D cells does‐ and time‐dependently (Figure S20A,B, Supporting Information). However, KIBRA‐2CS mutant appeared resistant to PA‐mediated degradation compared to KIBRA‐WT (Figure S20C, Supporting Information). In addition, treating the cells with MG132 rescued partially PA‐induced effects on endogenous KIBRA protein abundance in HCC1954 and TOV‐112D cells (Figure S20D, Supporting Information), while PA treatment substantially increased KIBRA ubiquitination in these cells (Figure S20E, Supporting Information). Together, these data indicate that PA promotes KIBRA degradation in the tested cancer cells. In line with the in vitro findings, IHC staining of pulmonary micrometastases in our tail vein injection xenograft model showed that tumor sections from mice fed with a Palm‐diet exhibited a decreased staining for KIBRA compared to the Olive‐diet group (Figure S20F, Supporting Information). However, when mice were fed with a Palm‐diet, tumor sections from mice bearing ZDHHC15‐depleted cells showed more staining for KIBRA compared to the matched control cells (Figure S20G, Supporting Information). These data suggest that PA treatment decreases KIBRA abundance in vivo through ZDHHC15.

### 2.8 Abrogation of KIBRA Palmitoylation Leads to KIBRA Nuclear Localization

**Figure 8:**
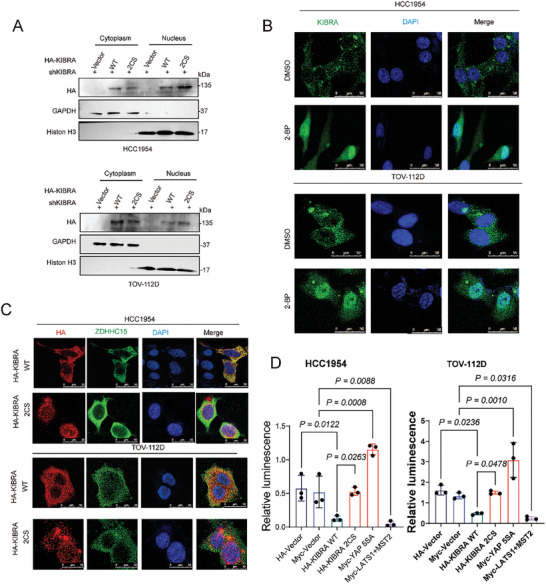
Abrogation of KIBRA palmitoylation leads to KIBRA nuclear localization. A) HCC1954 and TOV‐112D cells with stable knockdown of KIBRA were transfected with HA‐KIBRA WT and HA‐KIBRA 2CS constructs. The protein levels of exogenous KIBRA in the nucleus and cytoplasm were analyzed by IB. B) Immunofluorescence staining of KIBRA (green) on HCC1954 and TOV‐112D cells treated with 2‐BP (25 µm) for 24 h. Scale bars: 25 µm. C) Ectopic expression of HA‐KIBRA WT and HA‐KIBRA 2CS was performed in HCC1954 and TOV‐112D cells. Confocal microscopy was used to analyze the co‐localization of ZDHHC15 (green) and HA‐KIBRA (red). Scale bars: 25 µm. D) HCC1954 and TOV‐112D cells were transfected with the 8xGTIIC‐luciferase reporter along with KIBRA WT and its mutants. YAP 5SA and Myc‐tagged LATS1/MST2 were utilized as positive and negative controls, respectively. *P* values were assessed by one‐way ANOVA with Tukey's multiple comparisons test (*n =* 3). Data are representative of three independent experiments.

**Figure 9:**
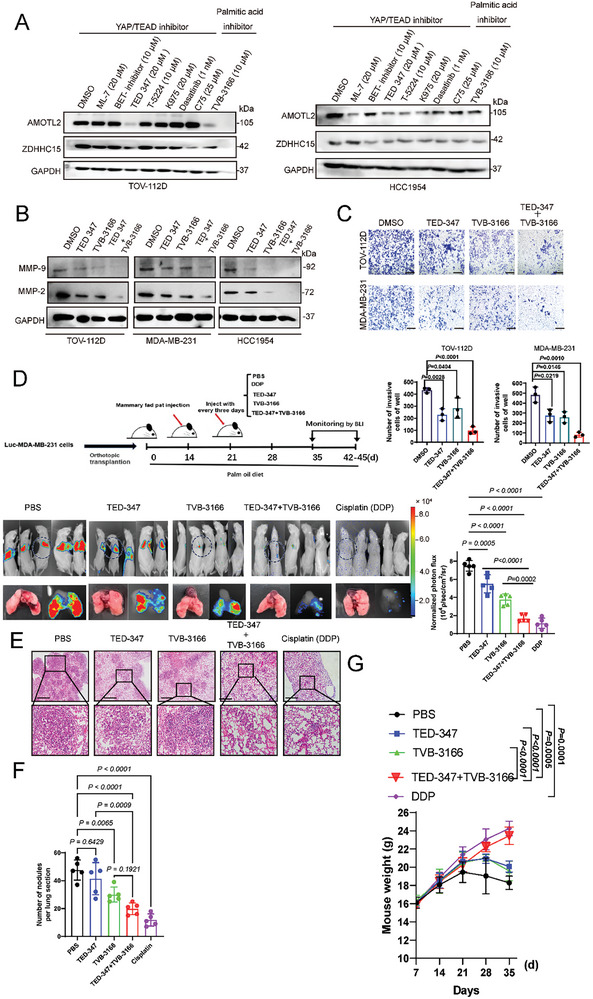
Combinatorial targeting of ZDHHC15‐YAP loop and fatty acid synthesis suppresses metastasis formation in mice fed with a Palm diet. A) YAP/TEAD inhibitors and fatty acid synthesis inhibitors induced AMOTL2 and ZDHHC15 protein expression in HCC1954 and TOV‐112D cells. These cells were treated with 20 µm ML‐7, 10 µm BET‐inhibitor, 20 µm TED‐347, 10 µm T‐5224, 20 µm K975, 1 nm Dasatinib, 25 µm C75 or 10 µm TVB‐3166 respectively for 24 h. B) HCC1954, TOV‐112D, and MDA‐MB‐231 cells were treated with DMSO, 20 µm TED‐347 or 10 µm TVB‐3166, or TED‐347 plus TVB‐3166 for 16 h. IB analysis of the expression levels of MMP‐9 and MMP‐2. C) The cell invasion capacity of TOV‐112D and MDA‐MB‐231 cells was evaluated using the trans‐well chamber after treatment with DMSO, 20 µm TED‐347 or 10 µm TVB‐3166, or TED‐347+TVB‐3166 for 16 h. *P* values were determined by the one‐way ANOVA, followed by Tukey's post hoc test (*n =* 3). D) Treatment plan for mice fed with a high‐fat diet plus palm oil in orthotopic transplantation models. The mice were treated with PBS, TED‐347 (20 mg kg^−1^) Cisplatin (10 mg kg^−1^) tail vein injection once every three days, and TVB‐3166 (60–80mg kg^−1^) with oral gavage once daily. Shown are images and BLI quantification of lung metastasis (*n =* 5 mice in each group). E) H&E staining in paraffin‐embedded xenograft tumor tissues from the indicated groups (*n =* 5 mice in each group). (F, G) The number of lung metastatic nodules (F), and weight (G) were counted and statistically analyzed. (*n =* 5 mice in each group). Scale bar: 100 µm. *P* values were determined by the one‐way ANOVA, followed by Tukey's post hoc test (D, F), and two‐way analysis of variance analysis (G). Data are representative of three independent experiments.

As protein palmitoylation plays a key regulatory role in protein targeting and trafficking,^[^ ^43^, ^44^ ^]^ we suspected that KIBRA palmitoylation might affect its subcellular localization. Under steady‐state conditions, ectopically expressed KIBTA‐WT was found predominantly in the cytoplasm (**Figure** **8**A). Remarkably, a great fraction of KIBRA‐2CS mutant was evident in the nuclear in transfected TOV‐112D cells (Figure 8A). In addition, 2‐BP treatment resulted in a fraction of endogenous KIBRA accumulated in the nuclear in HCC1954 and TOV‐112D cells (Figure 8B). Remarkably, endogenous ZDHHC15 co‐localized with KIBRA‐WT, but not KIBRA‐2CS mutant, in the cytoplasm in HCC1954 and TOV‐112D cells (Figure 8C). Together, our data suggest that KIBRA palmitoylation could limit its traffic to the nucleus. In addition, the palmitoylation‐deficient KIBRA‐2CS mutant failed to downregulate TEAD luciferase reporter activity as KIBRA‐WT did in both HCC1954 and TOV‐112D cells (**Figure** **9**D), indicating that the abrogation of palmitoylation of KIBRA impairs its effects on inhibiting YAP activity. We next examined whether abrogating the palmitoylation in KIBRA would affect its effect on cancer metastasis. In vitro experiments showed that the palmitoylation‐deficient KIBRA‐2CS mutant exhibited significantly increased invasion capacity in HCC1954 and TOV‐112D cells compared to KIBRA‐WT (Figure S21A, Supporting Information). Next, we injected luciferase‐expressing MDA‐MB‐231 cells with KIBRA‐WT or KIBRA‐2CS overexpression through the orthotopic transplantation of immunodeficient mice (Figure S21B, Supporting Information, 10 mice were randomly divided into 2 groups, *n =* 5 mice in each group,) Bioluminescence imaging showed that mice injected with KIBRA‐2CS overexpressing cells displayed a strong bioluminescent signal in lung compared to KIBRA‐WT overexpressing cells (Figure S21C, Supporting Information). We harvested the lung and assessed the number of metastasis lesions. The results showed that the injection of KIBRA‐2CS cells led to significantly more lung metastasis lesions compared to KIBRA‐WT cells (Figure S21D,E, Supporting Information). In addition, mice bearing the KIBRA‐WT overexpressing cells displayed a significant loss in body weight compared to KIBRA‐2CS mice (Figure S21F, Supporting Information).

### 2.9 Combinatorial Targeting of ZDHHC15‐YAP Loop And Fatty Acid Synthesis Displayed Augmented Benefit Against Metastasis Formation in Mice Fed with a Palm Diet

We next sought to pharmacologically target the ZDHHC15‐YAP loop to suppress PA‐promoted cancer metastasis. To this end, we selected a few commercially available inhibitors that directly or indirectly target either YAP signaling or fatty acid synthesis. While treatment with these compounds alone did not induce substantial cell death in HCC1954 and TOV‐112D cells, at least at the chosen concentrations and time frames, both TED‐347 and TVB‐3166 substantially suppressed the accumulation of AMOTL2 and ZDHHC15 proteins (Figure 9A). In addition, the combination of the two compounds exhibited a more pronounced effect on the abundance of MMP‐9 and MMP‐2 than either compound alone (Figure 9B). Consistently, in an invasion chamber assay, TED‐347 or TVB‐3166 treatment significantly decreased the invasion capacity of MDA‐MB‐231 and TOV‐112D cells compared to vehicle treatment (Figure 9C). We next performed in vivo experiments to ascertain the above in vitro findings. Mice‐bearing orthotopic xenografts initiated from MDA‐MB‐231 cells were supplemented with a Palm‐diet, and then used to evaluate the in vivo response to TED‐347 and TVB‐3166. Cisplatin, which has been recommended in combination with gemcitabine in the treatment of metastatic triple‐negative breast cancer,^[^ ^75^ ^]^ was cheered as a positive treatment control. Twenty‐five mice were randomly divided into five groups (BSA, Cisplatin, TED‐347, TVB‐3166, and TED‐347 plus TVB‐3166), 5 mice were used in each group. Bioluminescence imaging revealed that every single treatment significantly decreased the metastasis to the lung compared to the control treatment (Figure 9D,E). Of note, the combination of TED‐347 and TVB‐3166 showed a stronger effect on inhibiting metastasis to the lung compared with any single modality (Figure 9D–F). Additionally, we observed an increase in the body weight of mice in both the combination treatment group and the cisplatin group (Figure 9G). As expected, mice treated with cisplatin had less pulmonary micrometastases than vehicle control (Figure 9D–F).

## 3 Discussion

**Figure 10:**
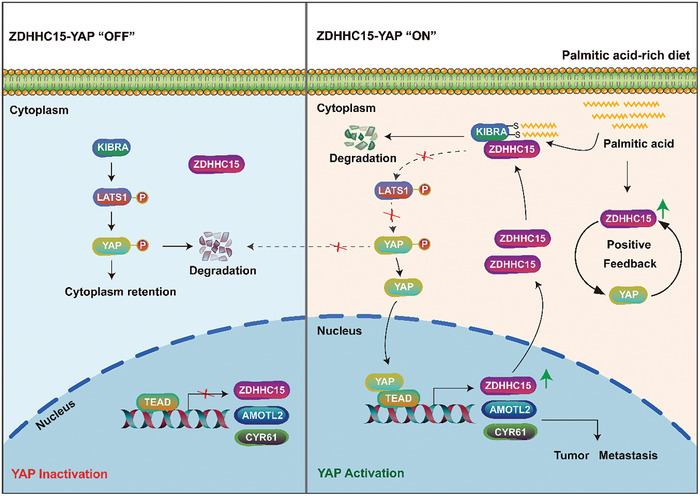
Schematic model of KIBRA palmitoylation and ZDHHC15‐YAP loop in cancer metastasis. Left: ZDHHC15‐YAP “OFF”, YAP is phosphorylated by LATS1 and is degraded in the cytoplasm, thereby TEAD transcription is shut down. Right: ZDHHC15‐YAP “ON” upon palmitic acid stimulation, ZDHHC15‐mediated KIBRA palmitoylation is enhanced, leading to KIBRA degradation, YAP activation, and TEAD transcription. Similar to other YAP target genes, ZDHHC15 is regulated by YAP/TEAD, and forms a feed‐forward loop in the Hippo pathway, thereby contributing to PA‐ and/or YAP‐triggered cancer metastasis.

The current study provides insights regarding the role and regulatory mechanism of Hippo/YAP signaling in PA‐promoted tumor metastasis. Our data indicate that a) YAP is critical for dietary PA‐enhanced metastasis; b) PA functions as an upstream signaling molecule to drive the ZDHHC15‐YAP loop, promoting metastasis in breast and ovarian cancers; c) PA promotes ZDHHC15‐mediated KIBRA palmitoylation, which affects KIBRA protein stability, subcellular localization, and activity toward YAP transcription; d) pharmacological inhibition of the ZDHHC15‐YAP loop and fatty acid synthesis suppresses PA‐promoted metastasis formation in mice. We thus suggest a model in which PA drives the ZDHHC15‐YAP feedback loop regulating the Hippo/YAP pathway and cancer metastasis (**Figure** **10**). To our knowledge, our report is the first study that explores the role and regulatory mechanism of YAP signaling as well as the therapeutic effect of inhibition of YAP and fatty acid synthesis in dietary PA‐promoted metastasis in breast and ovarian cancers.

Mounting evidence indicates that PA affects cancer metastasis through distinct mechanisms in different contexts.^[^ ^39^, ^76^ ^]^ Previous studies as well as our current in vivo investigation indicate that the Hippo/YAP pathway is implicated in cancer metastasis.^[^ ^39^, ^40^, ^77^ ^]^ A variety of studies documented that PA affected the Hippo/YAP signaling in diverse contexts, however, whether and how YAP plays a role in PA‐promoted metastasis remains unknown.

Here we present first evidence demonstrating that PA promotes metastasis in breast and ovarian cancers at least in part through YAP. Further more, dietary PA acts as a potent inducer of YAP signaling and activity in metastasis. Moreover, we screened and identified ZDHHC15 as a novel upstream inhibitor of the Hippo signaling pathway and presented striking evidence that ZDHHC15 functions in a feedback loop for the maintenance of YAP activity. Of important, ZDHHC15 expression is critical for dietary PA‐promoted metastasis in breast and ovarian cancer models. Therefore, PA drives a palmitoyltransferase ZDHHC15‐YAP feedback loop enforcing YAP activity in metastasis. Thus, our study reveals a previously unrecognized mechanism through which PA promotes metastasis in breast and ovarian cancers. In addition, we suggest PA as a previously unrecognized upstream stimulator of Hippo/YAP signaling in vivo. It should be noted that the Hippo‐YAP pathway can promote fatty acid oxidation, and may act as a sensor mechanism to catabolize the increase of fatty acids, thus contributing to PA‐affected cancer metastasis.

The palmitoyltransferase ZDHHC15 is associated with brain disorders as demonstrated in a variety of studies,^[^ ^78^, ^79^, ^80^ ^]^ and is implicated in glioma progression.^[^ ^72^, ^81^ ^]^ However, the function of ZDHHC15 beyond brain disorders and glioma remains poorly understood. In the current study, the clinical relevance of ZDHHC15 in breast and ovarian cancers is examined. We found that ZDHHC15 expression is increased in breast cancer patient samples, and high ZDHHC15 expression is linked to poor prognosis, suggesting that ZDHHC15 could serve as a prognostic indicator for breast cancer patients. In addition, elevated ZDHHC15 expression positively correlated with high‐grade ovarian cancer. Collectively, our study has established an oncogenic role of ZDHHC15 in breast and ovarian cancers. Importantly, as we observed that the ZDHHC15‐YAP feedback loop is functional in both breast and ovarian cancer cells, this strongly suggests that the loop might be conserved across multiple cancer cell types. Given the lack of traditionally druggable molecules in Hippo/YAP pathway, our data suggest an alternative way of targeting YAP, i.e., targeting ZDHHC15, at least in YAP‐ driven breast and ovarian cancers.

One of our important findings is that KIBRA, a critical upstream regulator of the Hippo/YAP pathway, is palmitoylated by ZDHHC15. Furthermore, KIBRA palmitoylation regulates its stability, localization, and activity toward Hippo/YAP pathway. Palmitoylation has been found to regulate the stability, trafficking, and functions of multiple cancer‐related proteins, such as Ras,^[^ ^82^ ^]^ epidermal growth factor receptor,^[^ ^83^ ^]^ and PD‐L1.^[^ ^84^ ^]^ Several studies suggest that palmitoylation plays a role in the regulation of Hippo/YAP pathway. In Drosophila, approximated (App) palmitoylated the intracellular domain of Fat, a transmembrane protein that is believed to function upstream in the Hippo pathway.^[^ ^85^ ^]^ In breast cancer cells, Scribble, a tumor suppressor involved in the regulation of the Hippo pathway,^[^ ^86^ ^]^ was found to be palmitoylated by ZDHHC7, and palmitoylation of Scribble is involved in the suppression of YAP activities.^[^ ^87^ ^]^ Intriguingly, recent studies found that TEAD transcription factors are autopalmitoylated, and autopalmitoylation of TEAD proteins regulates the transcriptional output of the Hippo pathway.^[^ ^47^, ^48^ ^]^ Furthermore, blocking TEAD auto‐palmitoylation by small molecule inhibitors provides a way to target the Hippo pathway and has displayed anticancer activities in preclinical investigations.^[^ ^49^, ^85^, ^88^, ^89^ ^]^ In the current study, we have uncovered palmitoylation as a previously unknown mechanism by which KIBRA regulates Hippo/YAP pathway. KIBRA has been recently gained great attention as it mediates a novel molecular mechanism underlying the upstream regulation of Hippo pathway, i.e., KIBRA forms biomolecular condensates to activate Hippo signaling.^[^ ^90^, ^91^ ^]^ Accumulating evidence suggests KIBRA functions as a tumor suppressor in the progression of various cancers,^[^ ^92^, ^93^, ^94^ ^]^ however, it remains poorly understood how KIBRA is regulated during cancer development. Given that the frequency of mutations for KIBRA is rare in most tumor types, the fine‐tuning of KIBRA expression and activity is required for its function in cancer progression. We found that PA treatment enhances KIBRA palmitoylation and degradation in a ZDHHC15‐dependent manner, consistent with previous studies showing that PA promotes protein palmitoylation.^[^ ^64^, ^65^ ^]^ Moreover, decreased KIBRA expression and concomitantly increased expression of YAP target genes were observed in tumor samples from mice supplemented with a Palm‐diet. Therefore, our findings shed novel insights into how KIBRA regulates the Hippo/YAP pathway, especially in a pathological context. Further studies will be geared at defining whether the palmitoylation of KIBRA plays a role in PA‐driven tumor metastasis.

Given our findings that the ZDHHC15‐YAP loop is hijacked by PA in tumor metastasis, it represents an important vulnerability that may offer a window of therapeutic efficacy. In addition, PA is the enzymatic product of FASN, the increased availability of de novo synthesized PA has been directly linked to the invasive advantage of prostate cancer cells.^[^ ^76^ ^]^ Therefore, blocking the activity of FASN in PA‐driven tumor metastasis might be an attractive therapeutic pathway. Our preclinical data support that combinatorial targeting of YAP and FASN displayed augmented benefit against PA‐promoted metastasis formation. Previous studies have suggested inhibition of fatty acid oxidation or YAP may merit exploration as a potential therapeutic strategy for mitigating PA‐ driven tumor metastasis.^[^ ^39^, ^95^ ^]^ In addition, targeting the FA receptor CD36 with CD36‐blocking antibodies therapeutically inhibited metastasis of OSCC tumors.^[^ ^50^ ^]^ Therefore, these studies and our current data advance the identification of potential targets for suppressing PA‐mediated metastasis.

Collectively, our study uncovers a ZDHHC15‐YAP feedback loop as a previously unrecognized mechanism underlying PA‐promoted tumor metastasis and underscores that PA serves as a signaling molecule driving the loop in tumor metastasis. In addition, we provide preclinical data to support targeting YAP and fatty acid synthesis in PA‐driven tumor metastasis.

## 4 Experimental Section

### Animal Studies

Four to five weeks‐old pathogen‐free female BALB/c and athymic nude mice were purchased from the Beijing Vital River Laboratory Animal Technology (Beijing, China) and maintained in a mouse‐specific pathogen‐free (SPF) facility for 1 week before injection to allow the mice to adjust to their new environment. MDA‐MB‐231(1.5 × 10^6^) or TOV‐112D cells (2 × 10^6^) or their derivatives were suspended in 100 µL of PBS and then injected into the tail vein of nude mice. The mice were monitored daily and were euthanized on days 25–28 after tail vein injection. Metastasis in the lungs was detected by bioluminescence imaging (BLI). All lobes of the lung were harvested and the number of macroscopic metastatic nodules on the surface of the lung was counted.

For metastasis formation, as previously described,^[^ ^56^, ^96^ ^]^ cells were harvested, washed twice in PBS, counted, and then re‐suspended in a 1:1 solution of PBS and Matrigel Matrix. Mice were anesthetized, a small incision was made to reveal the No.4 mammary fat pad, and luciferase‐labeled cells (5 × 10^6^ for MAD‐MB‐231 cells) were injected directly into the mammary fat pad. Tumor volume was assessed by caliper measurements using the following formula: π (width^2^ × length)/6 (mm^3^). The tumor volume of mice did not exceed 1500 mm^3^ permitted by the Institutional Animal Care and Use Committee. 7 days after primary tumors were removed, the mice were weighed every 7 d to detect their health status.

High‐fat diet experiments entailed feeding mice for 10 d with a 42% Kcal fat‐modified western diet supplemented with either 40% palm oil (P343608, Aladdin) or olive oil (O434216, Aladdin).^[^ ^8^, ^20^ ^]^ Animal survival was eventually analyzed. Besides, independent experimental groups used for corresponding survival analysis were performed under the same conditions. The mice were weighed every 7 d to detect their health status. All animal experiments were conducted strictly in compliance with animal protocols approved by the Institutional Animal Care and Use Committee of the Center for Animal Experiments of Dalian Medical University (Dalian, China).

### Bioluminescent Imaging

Bioluminescent imaging of mice was performed according to the published procedure with slight modifications.^[^ ^97^ ^]^ Briefly, d‐Luciferin potassium salt (450 mg kg^−1^ Beyotime, China) was subcutaneously injected into the abdominal cavity of mice. Images of the mice were acquired 10–20 min after d‐luciferin administration, and the peak photon flux within a region of interest was recorded and quantified using an IVIS Lumina System coupled with the Living Image data‐acquisition software program (Xenogen Corporation, Alameda, CA).

### Acyl‐PEG Exchange (APE) Assay

APE assay was performed according to the published procedure with slight modifications.^[^ ^98^, ^99^, ^100^ ^]^ In brief, trypsinized cells were washed three times with PBS and lysed in lysis buffer (5 mm triethanolamine, 150 mm NaCl, 4% SDS, 2 units benzenes, 5 mm PMSF, pH 7.4) with protease inhibitor cocktail. The cell lysates were treated with 10 mm of TCEP for 1 h at 55 °C and then incubated with 25 mm of NEM for 2 h at room temperature to reduce disulfide bonds and cap the free cysteine residues, respectively. The mixture was precipitated by the sequential addition of methanol, chloroform, and distilled water (4:3:2, relative to sample volume) into the 10‐mL Eppendorf tube. The protein pellets were washed twice with prechilled methanol, resuspended in buffer A (PBS, 4% SDS, 4 mm EDTA, pH 7.4, PMSF), and then incubated with 0.75 m of HAM (467804, sigma) for 1 h at room temperature to cleave palmitoylation thioester bonds. The impurity was removed by methanol chloroform‐water precipitation and the protein pellets were resuspended in buffer A for 2 h at room‐temperature. The samples were precipitated again by methanol‐chloroform‐water. Resuspend the pellet in 100 µL of buffer A containing 10 mm TCEP. Transfer to a 1.5 mL tube and add 20 mm mPEG‐5k 25 µL (63187, sigma) to the protein sample and mix by pipetting. Incubate for 1 h at 25 °C with end‐over‐end rotation. Used the above method to precipitate the protein for the last time and resuspended the dried pellet in 50 µL of buffer A then added an appropriate amount of 4× Laemmli sample buffer and, potentially heated the sample to 70–100 °C for 10 min. Immunoblotting analysis of PEGylated proteins, non‐PEG as negative control.

### Acyl‐biotin Exchange (ABE) Assay

To determine the level of protein palmitoylation, ABE assays were performed as previously described.^[^ ^72^, ^101^, ^102^, ^103^ ^]^ Cells were lysed in lysis buffer (50 mm Tris‐HCl, 150 mm NaCl, 1 mm MgCl_2_, 5 mm EDTA phosphatase inhibitor and protease inhibitor) for 1 h at 4 °C then centrifuged at 15 000r/4 °C for 15 min. The cell lysates were incubated with solubilization buffer (1% Triton X‐100, 5 mm EDTA, 50 mm Tris (pH 8.0), 4% SDS, and protease/phosphatase inhibitor cocktail) at 4 °C for 3 h, and endogenous KIBRA, or HA‐KIBRA was purified by specific antibodies and beads (shown as “input” in the indicated figure). Briefly, immunoprecipitated Protein A/G agarose was incubated with washing buffer supplemented with 50 mm N‐ethylmaleimide (50 mm Tris, pH 7.4, including 5 mm EDTA, 150 mm NaCl, and 1% Triton X‐100) at 4 °C for 1 h. Then, the beads were incubated with 1 m hydroxylamine (HAM, Sigma‐Aldrich), protease inhibitor, and phosphatase inhibitor containing lysis buffer with pH 7.2 at room temperature for 2 h. Each sample was divided into two parts, one omitting the HAM cleavage step (‐HAM) and one including the HAM step (+HAM). After being washed four times with lysis buffer and once with PBS then beads were treated with a thiol‐reactive biotin molecule, HPDP‐Biotin (Thermo Fisher Scientific), in PBS at 4 °C for 1 h. Gently washed all samples in PBS with PMSF three times. The immunoprecipitated samples were analyzed by immunoblot analysis using anti‐KIBRA antibody or anti‐HA antibody.

### LC‐MS/MS Analysis

Cell lysates obtained from HCC1954 cells were incubated with IgG or KIBRA (1:100) antibodies for 1 h, afterward incubated with protein G beads for 2 h at 4 °C.

TOV‐112D cells transfected with V5‐Vector or V5‐ZDHHC15 were lysed, and incubated with the V5‐tag antibody, and the immunoprecipitated protein samples were divided into two fractions. After the ABE reaction was completed, streptavidin beads were used to enrich the biotinylated proteins. Proteins enriched under HAM+ and HAM− conditions were identified using mass spectrometry. In the HAM+ sample, the palmitate residue was cleaved and exchanged with biotin. The HAM−condition served as a negative control. Proteins with at least two‐fold greater abundance in the HAM+ sample were considered to be candidate proteins. (#DIV/0 indicated that the number of HAM‐ group peptide segments was 0). All the protein sample preparation and data analysis for LC‐MS/MS were performed by Biotech Pack scientific (Beijing, China). Identified proteins that interacted with ZDHHC15 were listed in Table S6 (Supporting Information). Protein candidates that interacted with KIBRA were listed in Table S7 (Supporting Information). Protein candidates that were palmitoylated by ZDHHC15 were provided in Table S8 (Supporting Information).

### Score

PEAKS Peptide Score (−10lgP).  The PEAKS peptide score (−10lgP) was calculated for every peptide precursor spectrum match (PSM) reported by PEAKS DB, PEAKS PTM, and SPIDER. The score was derived from the *p*‐value that indicated the statistical significance of the peptide‐precursor spectrum match. When a peptide‐feature was associated with multiple spectra, the peptide score was the maximum among all PSM scores. For details of the scoring algorithm, please refer to the publication, “PEAKS DB: De Novo sequencing assisted database search for sensitive and accurate peptide identification” Mol Cell Proteomics. 2011 Dec 20. PEAKS Protein Score (−10lgP).  The PEAKS protein score (−10lgP) was calculated as the weighted sum of the −10lgP scores of the protein's supporting peptides. After removing any redundant peptides, the supporting peptides were sorted by −10lgP scores in descending order, and the k‐th ranked peptide contributed to the weighted sum with a weight of 1/k.

### Human Samples

Breast and ovarian cancer tissue microarray (TMA) were obtained from Shanghai Wellbio Biotechnology (Shanghai, China). ZDHHC15, YAP, and KIBRA expression were evaluated using the fully automated VIS DIA VisioMorph system (Visiopharm, Hoersholm, Denmark), using similar image processing principles as described previously. In brief, all TMA‐slides were scanned at 40× magnification using a Leica SCN400 slide scanner (Leica Biosystems, Wetzlar, Germany) and imported into the image analysis software program Visiopharm, and a digital image was recorded of each core. Histochemistry score, abbreviated as Histochemistry score, was a histological scoring method that processes immunohistochemistry, converting the number of positive cells and their staining intensity in each slice into corresponding numerical values, achieving the goal of semi‐quantitative staining of tissues. H‐Score (H‐SCORE = ∑ (*pi* × *i*) = (percentage of weak intensity × 1) + (percentage of rate intensity) × 2) + (percentage of strong intensity) × 3). In the formula, *pi* represents the proportion of positive signal pixel area per cell count. The H‐score was between 0 and 300, and the larger the data, the stronger the comprehensive positive intensity. Written informed consent was obtained from all patients, and data were analyzed anonymously. Pathological specimen information can be found in Tables S4 and S5 (Supporting Information).

### RNA Sequencing (RNA‐Seq)

RNA‐Seq was performed by the Novogene Corporation (Beijing, China). The sequencing libraries were constructed using NEBNext UltraTM RNA Library Prep Kit for Illumina (NEB, USA) according to the manufacturer's instructions. RNA‐seq data have been deposited at the NCBI Gene Expression Omnibus under the accession number GSE235254, GSE235258, and PRJNA986045.

### Study Approval

The Institutional Animal Care and Use Committee of the Dalian Medical University approved the use of animal models in this study (approval no. AEE22066).

Breast and ovarian cancer tissue microarray (TMA) were obtained from Shanghai Wellbio Biotechnology (Shanghai, China). The Shanghai Zhuoli Biotechnology Co., Ltd. Sample Library Ethics Committee approved the use the tumor specimens in this study (approval no. ZL2021‐11, ZL2015‐9).

### Statistics

All statistical analyses were performed using GraphPad Prism 9 (GraphPad Software). The data were presented as the mean ± SD. Analysis was performed using GraphPad Prism 9.0 software. Two‐tailed student's *t*‐test, one‐way ANOVA with Tukey's multiple comparison test, or one‐way ANOVA with Dunnett's multiple comparison test were used to evaluate the group difference. The survival rate of patients from the tissue array was assessed using the Kaplan–Meier method. In all analyses, All statistical tests were considered statistically significant when *p*‐values <0.05.

## Conflict of Interest

The authors declare no conflict of interest.

## Author Contributions

J.W., D.S., J.J., and L.H. contributed equally to this work. S.M., Y.W., and S.D. conceived and designed the study; J.W., D.S., J.J., L.H., K.F., N.S., and Y.Z. performed the experiments and analyzed the data; S.D. and S.M. wrote the paper with comments from all other authors.

## Supporting information



Supporting Information

Supporting Information

Supporting Information

Supporting Information

Supporting Information

Supporting Information

Supporting Information

Supporting Information

Supporting Information

Supporting Information

## Data Availability

The data that support the findings of this study are available from the corresponding author upon reasonable request.

## References

[1] Q. Zhou , L. Li , B. Zhao , K. L. Guan , Circ. Res. 2015, 116, 1431. 25858067 10.1161/CIRCRESAHA.116.303311 PMC4394208

[2] S. Y. Choi , H. Bae , S. H. Jeong , I. Park , H. Cho , S. P. Hong , D. H. Lee , C. K. Lee , J. S. Park , S. H. Suh , J. Choi , M. J. Yang , J. Y. Jang , L. Onder , J. H. Moon , H. S. Jeong , R. H. Adams , J. M. Kim , B. Ludewig , J. H. Song , D. S. Lim , G. Y. Koh , Nat. Commun. 2020, 11, 519. 31980640 10.1038/s41467-020-14293-1 PMC6981200

[3] V. A. Codelia , K. D. Irvine , Cell 2012, 150, 669. 22901800 10.1016/j.cell.2012.07.020

[4] F. X. Yu , B. Zhao , K. L. Guan , Cell 2015, 163, 811. 26544935 10.1016/j.cell.2015.10.044 PMC4638384

[5] S. Wu , J. Huang , J. Dong , D. Pan , Cell 2003, 114, 445. 12941273 10.1016/s0092-8674(03)00549-x

[6] B. Zhao , X. Ye , J. Yu , L. Li , W. Li , S. Li , J. Yu , J. D. Lin , C. Y. Wang , A. M. Chinnaiyan , Z. C. Lai , K. L. Guan , Genes Dev. 2008, 22, 1962. 18579750 10.1101/gad.1664408 PMC2492741

[7] F. Hamaratoglu , M. Willecke , M. Kango‐Singh , R. Nolo , E. Hyun , C. Tao , H. Jafar‐Nejad , G. Halder , Nat. Cell Biol. 2006, 8, 27. 16341207 10.1038/ncb1339

[8] G. Pascual , D. Domínguez , M. Elosúa‐Bayes , F. Beckedorff , C. Laudanna , C. Bigas , D. Douillet , C. Greco , A. Symeonidi , I. Hernández , S. R. Gil , N. Prats , C. Bescós , R. Shiekhattar , M. Amit , H. Heyn , A. Shilatifard , S. A. Benitah , Nature 2021, 599, 485. 34759321 10.1038/s41586-021-04075-0

[9] B. Zhao , Q. Y. Lei , K. L. Guan , Curr. Opin. Cell Biol. 2008, 20, 638. 18955139 10.1016/j.ceb.2008.10.001 PMC3296452

[10] X. Z. Liu , A. Rulina , M. H. Choi , L. Pedersen , J. Lepland , S. T. Takle , N. Madeleine , S. D. Peters , C. E. Wogsland , S. M. Grøndal , J. B. Lorens , H. Goodarzi , P. E. Lønning , S. Knappskog , A. Molven , N. Halberg , Nat. Commun. 2022, 13, 69. 35013251 10.1038/s41467-021-27734-2 PMC8748947

[11] P. Altea‐Manzano , G. Doglioni , Y. Liu , A. M. Cuadros , E. Nolan , J. Fernández‐García , Q. Wu , M. Planque , K. J. Laue , F. Cidre‐Aranaz , X. Z. Liu , O. Marin‐Bejar , J. Van Elsen , I. Vermeire , D. Broekaert , S. Demeyer , X. Spotbeen , J. Idkowiak , A. Montagne , M. Demicco , H. F. Alkan , N. Rabas , C. Riera‐Domingo , F. Richard , T. Geukens , M. De Schepper , S. Leduc , S. Hatse , Y. Lambrechts , E. J. Kay , et al., Nat Cancer 2023, 4, 344. 36732635 10.1038/s43018-023-00513-2 PMC7615234

[12] G. Zhao , Y. Tan , H. Cardenas , D. Vayngart , Y. Wang , H. Huang , R. Keathley , J. J. Wei , C. R. Ferreira , S. Orsulic , J. X. Cheng , D. Matei , Proc. Natl. Acad. Sci. U.S.A. 2022, 119, e2203480119. 36197994 10.1073/pnas.2203480119 PMC9564215

[13] J. Pan , Z. Fan , Z. Wang , Q. Dai , Z. Xiang , F. Yuan , M. Yan , Z. Zhu , B. Liu , C. Li , J. Experiment. Clin. Cancer Res. 2019, 38, 52. 10.1186/s13046-019-1049-7 PMC6360779 30717785

[14] X. Yu , W. Peng , Y. Wang , W. Xu , W. Chen , L. Huang , H. Xu , X. He , S. Wang , Q. Sun , W. Lu , Y. Xu , Cancers (Basel) 2023, 15, 388. 36672337 10.3390/cancers15020388 PMC9856364

[15] E. Giovannucci , E. B. Rimm , G. A. Colditz , M. J. Stampfer , A. Ascherio , C. G. Chute , W. C. Willett , J. Natl. Cancer Inst. 1993, 85, 1571. 8105097 10.1093/jnci/85.19.1571

[16] N. Kurahashi , M. Inoue , M. Iwasaki , S. Sasazuki , A. S. Tsugane , Cancer Epidemiol. Biomarkers Prev. 2008, 17, 930. 18708407 10.1158/1055-9965.EPI-08-0182

[17] S. Zhu , W. Jiao , Y. Xu , L. Hou , H. Li , J. Shao , X. Zhang , R. Wang , D. Kong , Life Sci. 2021, 286, 120046. 34653428 10.1016/j.lfs.2021.120046

[18] L. Lin , Y. Ding , Y. Wang , Z. Wang , X. Yin , G. Yan , L. Zhang , P. Yang , H. Shen , Hepatology 2017, 66, 432. 28073184 10.1002/hep.29033

[19] A. Nath , A. Oak , K. Y. Chen , I. Li , R. C. Splichal , J. Portis , S. Foster , S. P. Walton , C. Chan , Mol. Cancer Res. 2021, 19, 240. 33106375 10.1158/1541-7786.MCR-19-0480 PMC7864864

[20] D. W. Jeong , J. W. Park , K. S. Kim , J. Kim , J. Huh , J. Seo , Y. L. Kim , J. Y. Cho , K. W. Lee , J. Fukuda , Y. S. Chun , Nat. Commun. 2023, 14, 6370. 37828054 10.1038/s41467-023-42170-0 PMC10570296

[21] D. Pan , Genes Dev. 2007, 21, 886. 17437995 10.1101/gad.1536007

[22] K. F. Harvey , X. Zhang , D. M. Thomas , Nat. Rev. Cancer 2013, 13, 246. 23467301 10.1038/nrc3458

[23] J. Huang , S. Wu , J. Barrera , K. Matthews , D. Pan , Cell 2005, 122, 421. 16096061 10.1016/j.cell.2005.06.007

[24] B. Zhao , K. Tumaneng , K. L. Guan , Nat. Cell Biol. 2011, 13, 877. 21808241 10.1038/ncb2303 PMC3987945

[25] B. Zhao , L. Li , K. Tumaneng , C. Y. Wang , K. L. Guan , Genes Dev. 2010, 24, 72. 20048001 10.1101/gad.1843810 PMC2802193

[26] S. Wu , Y. Liu , Y. Zheng , J. Dong , D. Pan , Dev. Cell 2008, 14, 388. 18258486 10.1016/j.devcel.2008.01.007

[27] F. Yin , J. Yu , Y. Zheng , Q. Chen , N. Zhang , D. Pan , Cell 2013, 154, 1342. 24012335 10.1016/j.cell.2013.08.025 PMC3835333

[28] N. Zhang , H. Bai , K. K. David , J. Dong , Y. Zheng , J. Cai , M. Giovannini , P. Liu , R. A. Anders , D. Pan , Dev. Cell 2010, 19, 27. 20643348 10.1016/j.devcel.2010.06.015 PMC2925178

[29] R. Baumgartner , I. Poernbacher , N. Buser , E. Hafen , H. Stocker , Dev. Cell 2010, 18, 309. 20159600 10.1016/j.devcel.2009.12.013

[30] A. Genevet , M. C. Wehr , R. Brain , B. J. Thompson , N. Tapon , Dev. Cell 2010, 18, 300. 20159599 10.1016/j.devcel.2009.12.011 PMC2845807

[31] J. Yu , Y. Zheng , J. Dong , S. Klusza , W. M. Deng , D. Pan , Dev. Cell 2010, 18, 288. 20159598 10.1016/j.devcel.2009.12.012 PMC2858562

[32] M. Paramasivam , A. Sarkeshik , J. R. Yates 3rd , M. J. Fernandes , D. McCollum , Mol. Biol. Cell 2011, 22, 3725. 21832154 10.1091/mbc.E11-04-0300 PMC3183025

[33] B. Zhao , L. Li , Q. Lu , L. H. Wang , C. Y. Liu , Q. Lei , K. L. Guan , Genes Dev. 2011, 25, 51. 21205866 10.1101/gad.2000111 PMC3012936

[34] W. Wang , J. Huang , X. Wang , J. Yuan , X. Li , L. Feng , J. I. Park , J. Chen , Genes Dev. 2012, 26, 1959. 22948661 10.1101/gad.192955.112 PMC3435498

[35] Z. Meng , T. Moroishi , V. Mottier‐Pavie , S. W. Plouffe , C. G. Hansen , A. W. Hong , H. W. Park , J. S. Mo , W. Lu , S. Lu , F. Flores , F. X. Yu , G. Halder , K. L. Guan , Nat. Commun. 2015, 6, 8357. 26437443 10.1038/ncomms9357 PMC4600732

[36] M. Goudreault , L. M. D'Ambrosio , M. J. Kean , M. J. Mullin , B. G. Larsen , A. Sanchez , S. Chaudhry , G. I. Chen , F. Sicheri , A. I. Nesvizhskii , R. Aebersold , B. Raught , A. C. Gingras , Mol. Cell. Proteomics 2009, 8, 157. 18782753 10.1074/mcp.M800266-MCP200 PMC2621004

[37] F. X. Yu , B. Zhao , N. Panupinthu , J. L. Jewell , I. Lian , L. H. Wang , J. Zhao , H. Yuan , K. Tumaneng , H. Li , X. D. Fu , G. B. Mills , K. L. Guan , Cell 2012, 150, 780. 22863277 10.1016/j.cell.2012.06.037 PMC3433174

[38] S. Piccolo , T. Panciera , P. Contessotto , M. Cordenonsi , Nat Cancer 2023, 4, 9. 36564601 10.1038/s43018-022-00473-z PMC7614914

[39] C. K. Lee , S. H. Jeong , C. Jang , H. Bae , Y. H. Kim , I. Park , S. K. Kim , G. Y. Koh , Science 2019, 363, 644. 30733421 10.1126/science.aav0173

[40] S. Jagadeeshan , M. Prasad , M. Badarni , T. Ben‐Lulu , V. B. Liju , S. Mathukkada , C. Saunders , A. B. Shnerb , J. Zorea , K. M. Yegodayev , M. Wainer , L. Vtorov , I. Allon , O. Cohen , G. Gausdal , D. Friedmann‐Morvinski , S. C. Cheong , A. L. Ho , A. J. Rosenberg , L. Kessler , F. Burrows , D. Kong , J. R. Grandis , J. S. Gutkind , M. Elkabets , Cancer Res. 2023, 83, 1031. 36753744 10.1158/0008-5472.CAN-22-2586 PMC10073343

[41] M. C. Blair , M. D. Neinast , C. Jang , Q. Chu , J. W. Jung , J. Axsom , M. R. Bornstein , C. Thorsheim , K. Li , A. Hoshino , S. Yang , R. J. Roth Flach , B. B. Zhang , J. D. Rabinowitz , Z. Arany , Nat Metab 2023, 5, 589. 37100997 10.1038/s42255-023-00794-y PMC10278155

[42] N. Jaafar , A. Moleirinho , E. Kerkeni , K. Monastiri , H. Seboui , A. Amorim , M. J. Prata , S. Quental , Gene 2013, 517, 116. 23313820 10.1016/j.gene.2012.12.097

[43] P. J. Ko , S. J. Dixon , EMBO Rep. 2018, 19, e46666. 30232163 10.15252/embr.201846666 PMC6172454

[44] B. Zhou , Q. Hao , Y. Liang , E. Kong , Mol. Oncol. 2023, 17, 3. 36018061 10.1002/1878-0261.13308 PMC9812842

[45] M. D. Resh , Open Biol 2021, 11, 200414. 33653085 10.1098/rsob.200414 PMC8061759

[46] P. Chan , X. Han , B. Zheng , M. DeRan , J. Yu , G. K. Jarugumilli , H. Deng , D. Pan , X. Luo , X. Wu , Nat. Chem. Biol. 2016, 12, 282. 26900866 10.1038/nchembio.2036 PMC4798901

[47] C. L. Noland , S. Gierke , P. D. Schnier , J. Murray , W. N. Sandoval , M. Sagolla , A. Dey , R. N. Hannoush , W. J. Fairbrother , C. N. Cunningham , Structure 2016, 24, 179. 26724994 10.1016/j.str.2015.11.005

[48] N. G. Kim , B. M. Gumbiner , Proc. Natl. Acad. Sci. U.S.A 2019, 116, 9877. 31043565 10.1073/pnas.1819400116 PMC6525549

[49] Q. Li , Y. Sun , G. K. Jarugumilli , S. Liu , K. Dang , J. L. Cotton , J. Xiol , P. Y. Chan , M. DeRan , L. Ma , R. Li , L. J. Zhu , J. H. Li , A. B. Leiter , Y. T. Ip , F. D. Camargo , X. Luo , R. L. Johnson , X. Wu , J. Mao , Cell Stem Cell 2020, 26, 675. 32259481 10.1016/j.stem.2020.03.002 PMC7310193

[50] G. Pascual , A. Avgustinova , S. Mejetta , M. Martín , A. Castellanos , C. S. Attolini , A. Berenguer , N. Prats , A. Toll , J. A. Hueto , C. Bescós , L. Di Croce , S. A. Benitah , Nature 2017, 541, 41. 27974793 10.1038/nature20791

[51] J. Pan , Z. Fan , Z. Wang , Q. Dai , Z. Xiang , F. Yuan , M. Yan , Z. Zhu , B. Liu , C. Li , J. Experiment. Clin. Canc. Res. 2019, 38, 52. 10.1186/s13046-019-1049-7 PMC6360779 30717785

[52] T. Maier , S. Jenni , N. Ban , Science 2006, 311, 1258. 16513975 10.1126/science.1123248

[53] R. Ventura , K. Mordec , J. Waszczuk , Z. Wang , J. Lai , M. Fridlib , D. Buckley , G. Kemble , T. S. Heuer , EBioMed. 2015, 2, 808. 10.1016/j.ebiom.2015.06.020 PMC4563160 26425687

[54] J. Tome‐Garcia , P. Erfani , G. Nudelman , A. M. Tsankov , I. Katsyv , R. Tejero , B. Zhang , M. Walsh , R. H. Friedel , E. Zaslavsky , N. M. Tsankova , Nat. Commun. 2018, 9, 4020. 30275445 10.1038/s41467-018-06258-2 PMC6167382

[55] Y. Deng , Y. Matsui , W. Pan , Q. Li , Z. C. Lai , Protein Cell 2016, 7, 362. 27000077 10.1007/s13238-016-0258-5 PMC4853318

[56] B. Yu , J. Su , Q. Shi , Q. Liu , J. Ma , G. Ru , L. Zhang , J. Zhang , X. Hu , J. Tang , Nat. Commun. 2022, 13, 2192. 35449131 10.1038/s41467-022-29899-w PMC9023492

[57] Z. Zhang , J. Du , S. Wang , L. Shao , K. Jin , F. Li , B. Wei , W. Ding , P. Fu , H. van Dam , A. Wang , J. Jin , C. Ding , B. Yang , M. Zheng , X. H. Feng , K. L. Guan , L. Zhang , Mol. Cell 2019, 73, 7. 30472188 10.1016/j.molcel.2018.10.030

[58] Y. L. Hsu , J. Y. Hung , S. H. Chou , M. S. Huang , M. J. Tsai , Y. S. Lin , S. Y. Chiang , Y. W. Ho , C. Y. Wu , P. L. Kuo , Oncogene 2015, 34, 4056. 25381822 10.1038/onc.2014.333

[59] S. H. Malgundkar , I. Burney , M. Al Moundhri , M. Al Kalbani , R. Lakhtakia , A. Okamoto , Y. Tamimi , BMC Cancer 2020, 20, 374. 32366234 10.1186/s12885-020-06900-7 PMC7197128

[60] S. Weiler , T. Lutz , M. Bissinger , C. Sticht , M. Knaub , N. Gretz , P. Schirmacher , K. Breuhahn , Cancer Lett. 2020, 473, 164. 31904487 10.1016/j.canlet.2019.12.044

[61] J. Zhou , L. Zhang , W. Zhou , Y. Chen , Y. Cheng , J. Dong , FEBS J. 2019, 286, 963. 30600590 10.1111/febs.14743

[62] T. Matsumura , T. Fujimoto , A. Futakuchi , Y. Takihara , F. Watanabe‐Kitamura , E. Takahashi , M. Inoue‐Mochita , H. Tanihara , T. Inoue , PLoS One 2020, 15, e0242626. 33206726 10.1371/journal.pone.0242626 PMC7673499

[63] W. Su , S. Zhu , K. Chen , H. Yang , M. Tian , Q. Fu , G. Shi , S. Feng , D. Ren , X. Jin , C. Yang , J. Experiment. Clin. Canc. Res. 2021, 40, 88. 10.1186/s13046-021-01879-w PMC7923337 33648545

[64] L. H. Chamberlain , M. J. Shipston , Physiol. Rev. 2015, 95, 341. 25834228 10.1152/physrev.00032.2014 PMC4551212

[65] S. Chen , B. Zhu , C. Yin , W. Liu , C. Han , B. Chen , T. Liu , X. Li , X. Chen , C. Li , L. Hu , J. Zhou , Z. X. Xu , X. Gao , X. Wu , C. R. Goding , R. Cui , Nature 2017, 549, 399. 28869973 10.1038/nature23887 PMC5902815

[66] J. M. Draper , C. D. Smith , Mol. Membr. Biol. 2009, 26, 5. 19152182 10.1080/09687680802683839 PMC2635919

[67] S. Dupont , L. Morsut , M. Aragona , E. Enzo , S. Giulitti , M. Cordenonsi , F. Zanconato , J. Le Digabel , M. Forcato , S. Bicciato , N. Elvassore , S. Piccolo , Nature 2011, 474, 179. 21654799 10.1038/nature10137

[68] K. Bum‐Erdene , D. Zhou , G. Gonzalez‐Gutierrez , M. K. Ghozayel , Y. Si , D. Xu , H. E. Shannon , B. J. Bailey , T. W. Corson , K. E. Pollok , C. D. Wells , S. O. Meroueh , Cell Chem. Biol. 2019, 26, 378. 30581134 10.1016/j.chembiol.2018.11.010

[69] Q. Du , P. Liu , C. Zhang , T. Liu , W. Wang , C. Shang , J. Wu , Y. Liao , Y. Chen , J. Huang , H. Tan , Y. Zhao , M. Xia , J. Liu , S. Yao , Cell Death Dis. 2022, 13, 488. 35597782 10.1038/s41419-022-04926-2 PMC9124199

[70] J. Wan , A. F. Roth , A. O. Bailey , N. G. Davis , Nat. Protoc. 2007, 2, 1573. 17585299 10.1038/nprot.2007.225

[71] N. Adachi , D. T. Hess , P. McLaughlin , J. S. Stamler , J. Biol. Chem. 2016, 291, 20232. 27481942 10.1074/jbc.M116.725762 PMC5025705

[72] X. Fan , H. Yang , C. Zhao , L. Hu , D. Wang , R. Wang , Z. Fang , X. Chen , Stem Cell Res Ther 2021, 12, 107. 33541421 10.1186/s13287-021-02175-2 PMC7863430

[73] A. Percher , S. Ramakrishnan , E. Thinon , X. Yuan , J. S. Yount , H. C. Hang , Proc. Natl. Acad. Sci. U.S.A. 2016, 113, 4302. 27044110 10.1073/pnas.1602244113 PMC4843475

[74] N. Yokoi , Y. Fukata , A. Sekiya , T. Murakami , K. Kobayashi , M. Fukata , J. Neurosci. 2016, 36, 6431. 27307232 10.1523/JNEUROSCI.0419-16.2016 PMC5015780

[75] C. Liedtke , M. Thill , Breast Care (Basel, Switzerland) 2016, 11, 204. 27493622 10.1159/000446941 PMC4960356

[76] M. De Piano , V. Manuelli , G. Zadra , J. Otte , P. D. Edqvist , F. Pontén , S. Nowinski , A. Niaouris , A. Grigoriadis , M. Loda , M. Van Hemelrijck , C. M. Wells , Oncogene 2020, 39, 3666. 32139877 10.1038/s41388-020-1243-2 PMC7190568

[77] C. Li , S. Wang , Z. Xing , A. Lin , K. Liang , J. Song , Q. Hu , J. Yao , Z. Chen , P. K. Park , D. H. Hawke , J. Zhou , Y. Zhou , S. Zhang , H. Liang , M. C. Hung , G. E. Gallick , L. Han , C. Lin , L. Yang , Nat. Cell Biol. 2017, 19, 106. 28114269 10.1038/ncb3464 PMC5336186

[78] J. J. Shimell , B. S. Shah , S. M. Cain , S. Thouta , N. Kuhlmann , I. Tatarnikov , D. B. Jovellar , G. S. Brigidi , J. Kass , A. J. Milnerwood , T. P. Snutch , S. X. Bamji , Cell Rep. 2019, 29, 2422. 31747610 10.1016/j.celrep.2019.10.065

[79] R. Mejias , J. J. Rodriguez‐Gotor , M. Niwa , I. N. Krasnova , A. Adamczyk , M. Han , G. M. Thomas , Z. X. Xi , R. L. Huganir , M. V. Pletnikov , A. Sawa , J. L. Cadet , T. Wang , Transl Psychiatry 2021, 11, 65. 33462194 10.1038/s41398-020-01194-6 PMC7813841

[80] D. Casellas‐Vidal , I. Mademont‐Soler , J. Sánchez , A. Plaja , N. Castells , M. Camós , J. Nieto‐Moragas , M. Del Mar García , C. Rodriguez‐Solera , H. Rivera , J. Brunet , S. Álvarez , J. Perapoch , X. Queralt , M. Obón , Am. J. Med. Genet. A 2023, 191, 941. 36565021 10.1002/ajmg.a.63099

[81] Z. Y. Liu , T. Lan , F. Tang , Y. Z. He , J. S. Liu , J. Z. Yang , X. Chen , Z. F. Wang , Z. Q. Li , BMC Cancer 2023, 23, 420. 37161425 10.1186/s12885-023-10883-6 PMC10169355

[82] C. Busquets‐Hernández , G. Triola , Front. Mol. Biosci. 2021, 8, 659861. 33816563 10.3389/fmolb.2021.659861 PMC8010249

[83] K. B. Runkle , A. Kharbanda , E. Stypulkowski , X. J. Cao , W. Wang , B. A. Garcia , E. S. Witze , Mol. Cell 2016, 62, 385. 27153536 10.1016/j.molcel.2016.04.003 PMC4860254

[84] H. Yao , J. Lan , C. Li , H. Shi , J. P. Brosseau , H. Wang , H. Lu , C. Fang , Y. Zhang , L. Liang , X. Zhou , C. Wang , Y. Xue , Y. Cui , J. Xu , Nat. Biomed. Eng. 2019, 3, 306. 30952982 10.1038/s41551-019-0375-6

[85] H. Matakatsu , S. S. Blair , R. G. Fehon , J. Cell Biol. 2017, 216, 265. 28031421 10.1083/jcb.201609094 PMC5223609

[86] M. Cordenonsi , F. Zanconato , L. Azzolin , M. Forcato , A. Rosato , C. Frasson , M. Inui , M. Montagner , A. R. Parenti , A. Poletti , M. G. Daidone , S. Dupont , G. Basso , S. Bicciato , S. Piccolo , Cell 2011, 147, 759. 22078877 10.1016/j.cell.2011.09.048

[87] B. Chen , B. Zheng , M. DeRan , G. K. Jarugumilli , J. Fu , Y. S. Brooks , X. Wu , Nat. Chem. Biol. 2016, 12, 686. 27380321 10.1038/nchembio.2119 PMC4990496

[88] Y. Sun , L. Hu , Z. Tao , G. K. Jarugumilli , H. Erb , A. Singh , Q. Li , J. L. Cotton , P. Greninger , R. K. Egan , Y. Tony Ip , C. H. Benes , J. Che , J. Mao , X. Wu , Nat. Commun. 2022, 13, 6744. 36347861 10.1038/s41467-022-34559-0 PMC9643419

[89] T. T. Tang , A. W. Konradi , Y. Feng , X. Peng , M. Ma , J. Li , F. X. Yu , K. L. Guan , L. Post , Mol. Cancer Ther. 2021, 20, 986. 33850002 10.1158/1535-7163.MCT-20-0717

[90] L. Wang , K. Choi , T. Su , B. Li , X. Wu , R. Zhang , J. H. Driskill , H. Li , H. Lei , P. Guo , E. H. Chen , Y. Zheng , D. Pan , Cell 2022, 185, 4376. 36318920 10.1016/j.cell.2022.09.036 PMC9669202

[91] T. T. Bonello , D. Cai , G. C. Fletcher , K. Wiengartner , V. Pengilly , K. S. Lange , Z. Liu , J. Lippincott‐Schwartz , J. M. Kavran , B. J. Thompson , EMBO J. 2023, 42, 112863. 10.15252/embj.2022112863 PMC10015380 36807601

[92] S. Moleirinho , N. Chang , A. H. Sims , A. M. Tilston‐Lünel , L. Angus , A. Steele , V. Boswell , S. C. Barnett , C. Ormandy , D. Faratian , F. J. Gunn‐Moore , P. A. Reynolds , Oncogene 2013, 32, 1821. 22614006 10.1038/onc.2012.196

[93] J. F. Knight , V. Sung , E. Kuzmin , A. L. Couzens , D. A. de Verteuil , C. Ratcliffe , P. P. Coelho , R. M. Johnson , P. Samavarchi‐Tehrani , T. Gruosso , H. W. Smith , W. Lee , S. M. Saleh , D. Zuo , H. Zhao , M. C. Guiot , R. R. Davis , J. P. Gregg , C. Moraes , A. C. Gingras , M. Park , Cell Rep. 2018, 22, 3191. 29562176 10.1016/j.celrep.2018.02.095 PMC5873529

[94] J. Park , J. S. Kim , J. H. Nahm , S. K. Kim , D. H. Lee , D. S. Lim , Mol. Cells 2020, 43, 491. 32451369 10.14348/molcells.2020.0093 PMC7264477

[95] P. Altea‐Manzano , G. Doglioni , Y. Liu , A. M. Cuadros , E. Nolan , J. Fernández‐García , Q. Wu , M. Planque , K. J. Laue , F. Cidre‐Aranaz , X. Z. Liu , O. Marin‐Bejar , J. Van Elsen , I. Vermeire , D. Broekaert , S. Demeyer , X. Spotbeen , J. Idkowiak , A. Montagne , M. Demicco , H. F. Alkan , N. Rabas , C. Riera‐Domingo , F. Richard , T. Geukens , M. De Schepper , S. Leduc , S. Hatse , Y. Lambrechts , E. J. Kay , et al., Nat Cancer 2023, 4, 344. 36732635 10.1038/s43018-023-00513-2 PMC7615234

[96] Y. Li , X. He , X. Lu , Z. Gong , Q. Li , L. Zhang , R. Yang , C. Wu , J. Huang , J. Ding , Y. He , W. Liu , C. Chen , B. Cao , D. Zhou , Y. Shi , J. Chen , C. Wang , S. Zhang , J. Zhang , J. Ye , H. You , Nat. Commun. 2022, 13, 6350. 36289222 10.1038/s41467-022-34209-5 PMC9605963

[97] X. Li , X. Qian , B. Wang , Y. Xia , Y. Zheng , L. Du , D. Xu , D. Xing , R. A. DePinho , Z. Lu , Nat. Cell Biol. 2020, 22, 282. 32066906 10.1038/s41556-020-0471-6

[98] Z. Zhang , X. Li , F. Yang , C. Chen , P. Liu , Y. Ren , P. Sun , Z. Wang , Y. You , Y. X. Zeng , X. Li , Nat. Commun. 2021, 12, 5872. 34620861 10.1038/s41467-021-26180-4 PMC8497546

[99] D. J. Speca , E. Diaz , J. Visual. Exp.: JoVE 2020, 10.3791/61018 PMC7220753 32281973

[100] M. T. Forrester , D. T. Hess , J. W. Thompson , R. Hultman , M. A. Moseley , J. S. Stamler , P. J. Casey , J. Lipid Res. 2011, 52, 393. 21044946 10.1194/jlr.D011106 PMC3023561

[101] L. Wang , J. Cai , X. Zhao , L. Ma , P. Zeng , L. Zhou , Y. Liu , S. Yang , Z. Cai , S. Zhang , L. Zhou , J. Yang , T. Liu , S. Jin , J. Cui , Mol. Cell 2023, 83, 281. 36586411 10.1016/j.molcel.2022.12.002

[102] C. H. Hurst , D. Turnbull , F. Plain , W. Fuller , P. A. Hemsley , BioTechniques 2017, 62, 69. 28193150 10.2144/000114516 PMC5400063

[103] M. Zhang , L. Zhou , Y. Xu , M. Yang , Y. Xu , G. P. Komaniecki , T. Kosciuk , X. Chen , X. Lu , X. Zou , M. E. Linder , H. Lin , Nature 2020, 586, 434. 33029007 10.1038/s41586-020-2799-2 PMC7874492

[104] C. A. Ocasio , M. P. Baggelaar , J. Sipthorp , A. Losada de la Lastra , M. Tavares , J. Volarić , C. Soudy , E. M. Storck , J. W. Houghton , S. A. Palma‐Duran , J. I. MacRae , G. Tomić , L. Carr , J. Downward , U. S. Eggert , E. W. Tate , Nat. Biotechnol. 2024, 42, 1548. 38191663 10.1038/s41587-023-02030-0 PMC11471619

