# Supplementary material for: Dietary Palmitic Acid Drives a Palmitoyltransferase ZDHHC15‐YAP Feedback Loop Promoting Tumor Metastasis
Source: Adv Sci (Weinh). 2024 Dec 16;12(6):2409883. doi: 10.1002/advs.202409883 (PMC11809420; doi:10.1002/advs.202409883)
Supplement: Supplementary file 1 — Supporting Information [file ADVS-12-2409883-s006.pdf]

ADVANCED SCIENCE
Open Access

Supporting Information

for *Adv. Sci.*, DOI 10.1002/advs.202409883

Dietary Palmitic Acid Drives a Palmitoyltransferase ZDHHC15-YAP Feedback Loop Promoting Tumor Metastasis

Jianxin Wang, Dachuan Shen, Jian Jiang, Lulu Hu, Kun Fang, Chunrui Xie, Ning Shen, Yuzhao Zhou, Yifei Wang, Sha Du* and Songshu Meng**

1. Experimental Section

Cell lines and reagents

Human breast cancer cell lines HCC1954, HCC1806, MDA-MB-231, and T47D human mammary epithelial cell line MCF-10A and human embryonic kidney (293T) and human ovarian cancer cell lines TOV-112D, OVCAR4 and CAOV3 were purchased from the American Type Culture Collection. Cells were cultured at 37°C in 5% CO2 with DMEM or RPMI-1640 (Gibco) medium supplemented with 10% fetal bovine serum, 100 unit/mL penicillin and 100 mg/mL streptomycin. The compounds were used as below: TED-347 (HY-125269, MCE), ML-7 hydrochloride (HY-15417, MCE), K975 (HY-138565, MCE), T-5224 (HY-12270, MCE), BET- bromodomain inhibitor 1 (HY-131061, MCE), XMU-MP-1 (S8334, Selleck), TVB-3166 (HY-120394, MCE), VT107 (HY-134957, MCE), C75 (HY-12364, MCE), Dasatinib (SML2589, Sigma), MG132 (S2619, Selleck) and Bafilomycin A1(S1413, Selleck), Fatty-acid-free bovine serum albumin (FFBSA, A7030, Sigma) and palmitic acid (P9767, Sigma-Aldrich). Palmitic acid was complexed with fatty-acid-free BSA at a 5:1 molar ratio. Palmitic acid was dissolved and saponified in 0.1 M NaOH at 70°C. Fatty-acid-free BSA was reconstituted in 150 mM NaCl to obtain a 2 mM BSA solution and kept warm at 37 °C. Palmitic acid was then added to obtain a 10 mM fatty acid, 2 mM BSA stock solution. The solution was incubated for 2 hours at 37°C with vigorous shaking to encourage coupling of the fatty acid to BSA. The stock solution was kept at−20°C. The control BSA solution was prepared identically except that palmitic acid was absent from the preparation.

Plasmids

The expression plasmids of Flag tagged-ZDHHC2, 3 ,7, 9, 11, 12, 13, 15, 20, 21, 22 and 24 were purchased from WZ Biosciences (China) and Flag or V5 tagged-ZDHHC1, 4, 5, 6, 8, 14, 16, 17, 18, 19, 23, HA tagged-KIBRA, pGEX-6P-1 and PGL3-basic-ZDHHC15-prompter were purchased from Miaoling Biology (China). Mutations in ZDHHC15 or KIBRA were introduced by PCR-based site-directed mutagenesis. The plasmids of GFP-MST1, Myc-LATS1, Myc-LATS2, Myc-YAP, Flag-AMOT, Myc-YAP-5SA and Myc-YAP-S94A were kindly provided by Prof. Bin Zhao (Zhejiang University, China).

V5 tagged ZDHHC15 WT (wild type) or DHHS mutation and HA- KIBRA WT or 2CS mutation were subcloned into the pCDH-puro lentiviral vector by standard molecular cloning procedures. Stable cell lines knocking down or overexpressing ZDHHC15 were established. The primers used for construction of these plasmids were listed in Table S9

shRNA-knockdown, siRNA and transfection assays.

The lentiviral vectors encoding short hairpin RNAs (GIPZ shRNAs) targeting ZDHHC15, KIBRA, and YAP, respectively, and scrambled shRNA were purchased from Dharmacon (America). V5-tagged ZDHHC15 was subcloned into the PCDH-puro lentiviral vector by standard molecular cloning procedures. The plasmids were transiently transfected into cells with Lipofectamine 3000 (Invitrogen, CA) according to the manufacturer’s protocol. Cells were harvested and analyzed 36 hours after transfection. Multiple monoclonal cultures were screened for shRNAs by western

blotting analysis. YAP-depleted HCC1954 cells were established in our lab[1,2]. ZDHHCs siRNA sequence and PCR primer reference[3,4] siRNA oligonucleotides were purchased from Ribobio (Guangzhou, China) and sequences are as follows:

siYAP-1: GACAUCUUCUGGUCAGAGA

siYAP-2: GAGATGGAATGAACATAGA

siTEAD1-1GGCAUGCCAACCAUUCUUA

siTEAD1-2CACCUACCAGAGAAAUAUA

siTEAD2-1GCCAGAUGCAGUUGAUUCU

siTEAD2-2CGAAGGAAAUCAAGGGAAA

siTEAD3-1CAGCCACAUACAGGUUCUA

siTEAD3-2GUAUUUAUGAGUUUCAAAUGA

siTEAD4-1GGAACAAACUGUGCCUGAA

siTEAD4-2GGACACUACUCUUACCGCA

Nontargeting control UUCUCCGAACGUGUCACGU

ZDHHC15 siRNA (sc-91146) were purchased from Santa Cruz.

Antibodies

The following antibodies were purchased from Cell Signaling Technology (America): LATS1 (3477S), P-LATS1 (9157S), P-YAP127 (4911S), V5-tag (13202S), Myc-tag (2278S), KIBRA (8774), MST1 (3682S), MST2 (3952S), pMST (3681S), Merlin (12888), SAV1 (13301S), FRMD6 and MOB1 (3863). Anti-V5 (P/N46-0705) was purchased from Invitrogen Thermo Fisher Scientific (America). YAP1 (NB110-58358) was purchased from NOVUS (America). ZDHHC15(21627-1-AP), MMP-2 (10373-2-

AP), GAPDH (10494-1-AP), AXL (13196-1-AP) and Flag-tag (20543-1-AP) were obtained from Proteintech (America). HA-tag (H6908) antibody was purchased from Sigma (America). Antibodies against ZDHHC15 (ab121203), LAMP2 (25631) and MMP-9(38898) were purchased from Abcam, Antibodies against CYR61(A1111), AMOTL2(A2348), ITGAV (A2091), LIMD1 (A17585), ODC1 (A3898) and Histon H3 (A16723) were purchased from Abclonal (China).

Luciferase reporter assay

For Luciferase assay, 0.1μg YAP/TAZ responsive Luciferase gene (8xGTIIC Luciferase reporter gene) or empty vector control (pGL3), 0.1 μg specific particle and 0.02 μg pRL TK-Renilla (Promega) were used to transfect 60% fusion cells in a 24 well plate for 36 hours according to the manufacturer's scheme with lipo3000 transfection reagents (Thermo Fisher). For ZDHHC15 promoter activity detection, pGL3.0 basic with the wild-type ZDHHC15 promoter and the promoter with mutated TEAD1 binding sites were co-transfected with or without TEAD1. A pRL TK- Renilla luciferase control reporter vector (Promega) was utilized as a negative control. A dual-luciferase assay was performed 36-48 h after co-transfection using the Enspire2300 (Perkin Elmer) following the manufacturer’s recommendation.

Immunoblotting and immunoprecipitation

Cells were lysed in RIPA lysis buffer (10mM Tris-HCl, pH 8.0, 140mM NaCl, 1mM EDTA, 1% Triton X-100, 0.1% sodium deoxycholate, 0.1% SDS, pH 7.4) supplemented with cocktail of protease and phosphatase inhibitors. To extract cytoplasmic and nuclear proteins, cells were processed with the NEPER Nuclear and

Cytoplasmic Extraction Kit (ThermoFisher, 78833). Protein samples were separated by SDS-PAGE and then transferred to nitrocellulose membrane. The membrane was incubated with primary antibody against each target protein at 4°C overnight. Afterward, the membrane was incubated with secondary antibody at room temperature for 1 hour. Detection was performed using SuperSignalTM Substrates (Thermo Fisher, 34577 or 34095).

For immunoprecipitation, cells grown in 100-mm dish were lysed in 1 ml lysis buffer and cleared by centrifuging at 15000 r (10 min, 4°C). A 50 μL aliquot of the lysate was taken for IB assay to examine protein expression and the remaining lysate was added 1 μg appropriate antibody at 4°C for 1h then add 30 μL protein G agarose beads and incubated at 4 °C for 2 hours before washed 5 times with Wash buffer (10 mM Tris-Hcl, pH 7.4, including 5 mM EDTA, 150 mM NaCl, and 1% Triton X-100). The beads were then boiled in 20 μL 2×SDS loading buffer for 5 min to collect the samples for SDS-PAGE.

GST-pull down

Refer to previous experimental methods in our laboratory[5]. E. coli BL21 were transfected with pGEX-6P-1 bacterial expression plasmids encoding GTS-ZDHHC15 or GST-KIBRA (wild type or section mutation). Recombinant protein expression was induced by 1 mM IPTG for 16 hours at 25°C. When the medium absorbance value of OD600 was 0.6, GST-tagged proteins were purified by using a GST-tagged protein purification kit (Cat# P2262, Beyotime) with manufacturer's instructions. Briefly, bacteria were collected by centrifugation (4000 x g, 4°C, 20 min) and lysed with ice

cold lysis buffer (Cat# P2262-2, Beyotime) with ultrasonic fragmentation (procedure: 200 W, 20 seconds, and 10 seconds for interval, 6 cycles). Supernatants were collected by centrifugation (10000 x g, 4°C, 20 min), and then were loaded onto BeyoGold™ GST-tag resin columns, eluted with glutathione containing (10 mM) elution buffer to obtain the purified GST tagged proteins. For GST pull down, GST fusion proteins were mixed with cell lysates. The binding reaction was mixed at 4 °C for 2 hours. The beads were washed 5 times with NETN buffer (50 mM Tris-HCl pH 8.0,100 mM NaCl, 0.1% TritonX-100 1 mM EDTA, PMSF). The bound proteins were eluted and visualized by Immunoblotting.

Chromatin immunoprecipitation (ChIP) assay

HCC1954 or TOV-112D cells transfected with the ZDHHC15 overexpressing plasmid were cross-linked with 1% formaldehyde for 5 min at 37°C and were stopped by 0.125M glycine for 10 min at room temperature. The cells were washed twice with cold PBS containing PMSF and harvested in SDS Lysis buffer from a ChIP Assay Kit (P2078; Beyotime Biotechnology; Shanghai, China). Then, the ultrasonic fragmentation was used to sonicate the sample (260 W, 25 times, 30 s/60 s off) at 4°C. Protein A+G Agarose were used to preclear the whole cell lysate for 30 min at 4°C. After the 10 % input sample was extracted, the sample were divided equally and incubated with anti-V5 tag or control Vector antibody overnight at 4°C. Thereafter, the beads were washed sequentially with Low-Salt Immune Complex Wash Buffer, High-Salt Immune Complex Wash Buffer, LiCl Immune Complex Wash Buffer and TE Buffer (twice) for 5 min at 4°C rotation. DNA-protein complexes were eluted with elution buffer (1%

SDS and 0.1 M NaHCO3) and de-crosslinked by adding 0.2 M NaCl and heating for 4 hours at 65°C. Then, the proteins were digested with proteinase K for 1 hour at 45°C, and the DNA segments were purified by a DNA Purification Kit (D0033; Beyotime Biotechnology; Shanghai, China) and used for qPCR reaction. The primers for ChIP-PCR are listed Table S9.

Quantitative real-time PCR.

Total RNAs were extracted from cells using a SteadyPure Quick RNA Extraction kit (AG21023, Accurate Biotechnology). Complementary DNAs were synthesized by reverse transcription using Transcript reverse transcriptase (TransSgen). cDNAs were then used for quantitative real-time PCR with gene-specific primers and SYBR Green Pro Taq HS qPCR mix (AG11701, Accurate, Biology) using the Mx3000P, Real-Time PCR System (Agilent). The relative abundance of messenger RNAs was calculated by normalizing to GAPDH mRNA. The Primer sequence as shown in Table S9.

Transwell invasion assay

Invasion experiments were carried out with the polycarbonate membrane, which was pre-coated with 40 μL Matrigel. The cells resuspended in 200 μL serum-free media were seeded into the upper chamber after pre-treatment with mitomycin C, and a total of 800 μL of complete medium was added into the lower chamber. After incubation, the cells were fixed and stained with 0.1% crystal violet, and then the cells were

.

Immunofluorescence

Cells were seeded on coverslips and treated as indicated. Cells were washed once with PBS and then fixed using 4% paraformaldehyde for 20 min at room temperature. Fixed cells were washed twice with PBS before permeabilization with 0.5% Triton X-100 in PBS (PBST) for 10 min at 4°C. All subsequent wash steps were with PBST. Following permeabilization, cells were washed twice, and then incubated with the appropriate primary antibody for 1 hour at 37°C. Following primary incubation, cells were washed three times, then incubated with the appropriate secondary antibody for 1 hour at 37°C. Following secondary incubation, coverslips were washed four times and then mounted on glass slides using mounting media containing DAPI (Vectashield). Cells were imaged using a Leica TCS SP5II microscope and quantified using image J (NIH).

Immunohistochemistry

Paraffin-embedded tissue sections were dewaxed using a decreasing xylene/alcohol series. Briefly, the processed sections were blocked with 3% BSA and incubated with antibodies as below: ZDHHC15 (Sigma, 1:50), KIBRA (1:100), Ki67 (1:100), AMOTL2 (1:100), CYR61 (1:100), and YAP (1:100). The DAB Detection Kit (SAP-9100, ZSGB-Bio, Beijing) was used to develop the staining signals according to the protocols provided for the streptavidin-peroxidase system (Sangon Biotech, China). Hematoxylin was used for counterstaining.

Bioinformatics analysis

To comprehensively explore the expression pattern and prognostic implications of ZDHHC15 in breast and ovarian cancers, TCGA-BRCA, TCGA-OV RNA-seq and clinical data were downloaded from UCSC XENA (https://xenabrowser.net/datapages/)

and Gene Expression Omnibus (GEO). Curated progression-free survival data were obtained from an integrated TCGA Pan-Cancer Clinical Data Resource (TCGA-CDR). TCGA-BRCA were classified into ER+, HER2+, LumA, LumB and TNBC according to ER, PR and HER2 status assessed by Immunohistochemistry (IHC). LumA and LumB were defined as ER/PR+Her2- luminal A-like and ER/PR+ Luminal B-like by combining IHC-based and PAM50-based intrinsic subtype. Only primary tumor samples were retained for further analysis. Survival analysis was conducted via the 'survminer' package. Breast or ovarian cancer patients were categorized into High and Low ZDHHC15 expression group using the median expression as cutoff points and survival curves were based on Kaplan-Meier estimates. The association between ZDHHC15 and YAP or KIBRA in breast and ovarian cancer were downloaded from the TIMER2.0 database (http://timer.cistrome.org/) [6] in the "Gene" function of the "Correlation" section. We visualized the statistical Spearman correlations between ZDHHC15 and YAP or KIBRA mRNA expression in breast and ovarian cancers.

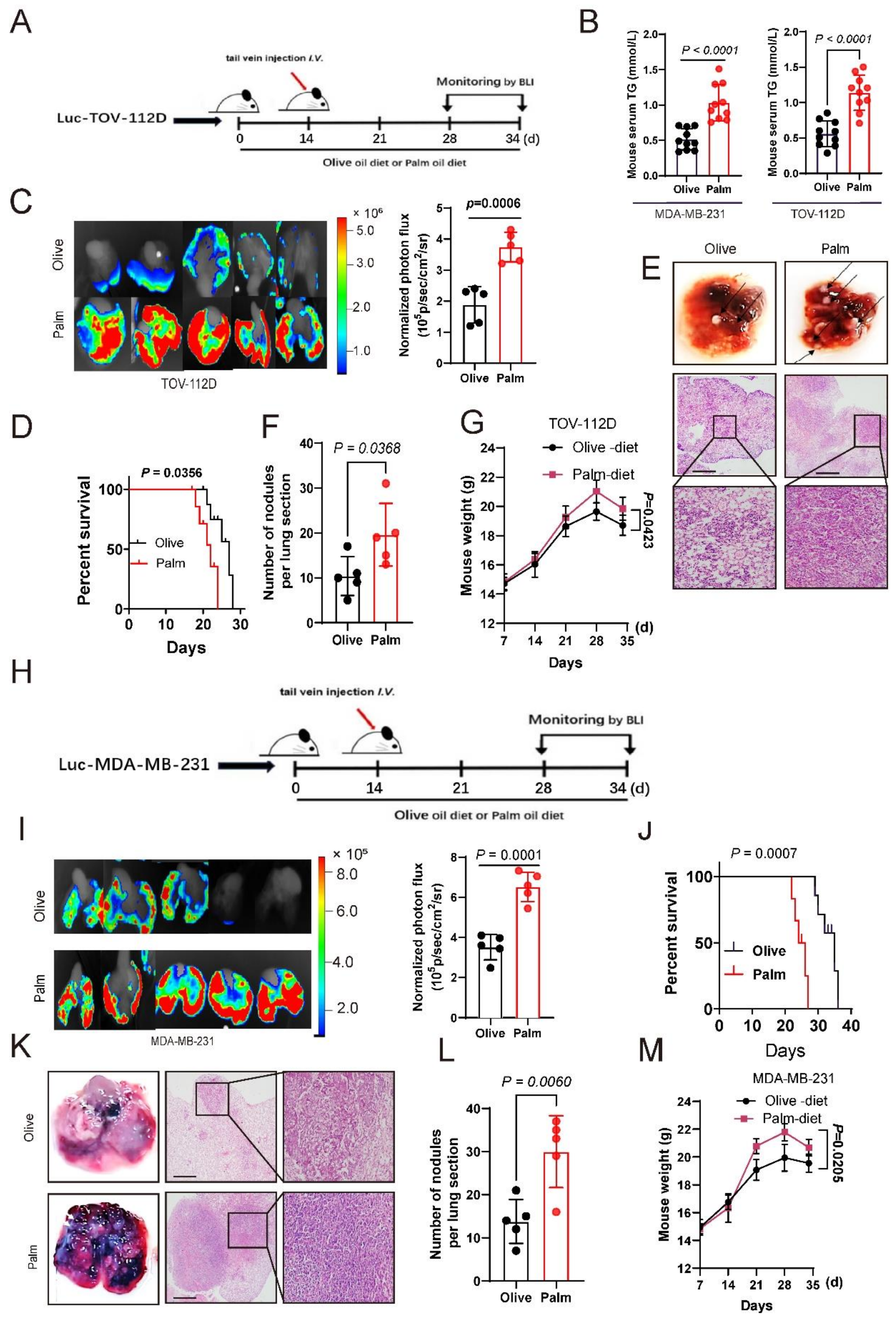

Figure S1 (Related to Figure1). Dietary palmitic acid promotes metastasis in breast and ovarian cancers. **A**) Treatment plan for a high-fat diet supplemented with either palm oil (PA) or olive oil (OA) in a tail vein injection model. **B)** Triglyceride levels in

mouse serum detected using ELISA, *P* values were determined by the two-tailed Student's t test (n=10). **C)** Bioluminescent images (BLI)，quantification of lung metastasis tumours generated from mice injected with luc-TOV-112D cells via tail vein on the 14th day and observed an BLI of mice at 14-21 days after injection (n=5 mice in each group). **D)** The survival rate was counted and statistically analyzed. (n=5 mice in each group) **E)** Representative images of H&E staining (n=5 mice in each group). **(F, G)** The number of lung metastatic nodules **(F)** and weight **(G)** was counted and statistically analyzed. (n=5 mice in each group). **H)** Treatment plan for a high-fat diet supplemented with either palm oil (PA) or olive oil (OA) in a tail vein injection model. (n=5 mice in each group). **I)** Bioluminescent images (BLI)，quantification of lung metastasis tumours generated from mice injected with luc-MDA-MB-231 cells via tail vein on the 14th day and observed an BLI of mice at 14-21 days after injection (n=5 mice in each group). **J)** The survival rate was counted and statistically analyzed. (n=5 mice in each group) **K)** Representative images of H&E staining (n=5 mice in each group). **(L, M)** The number of lung metastatic nodules **(L)**, and weight **(M)** change was counted and statistically analyzed. (n=5 mice in each group). *P* values were determined by the unpaired Student's t test (B, C, F, I, L), two-way analysis of variance analysis (G, M), Log-rank Mantel-Cox test (D, J).

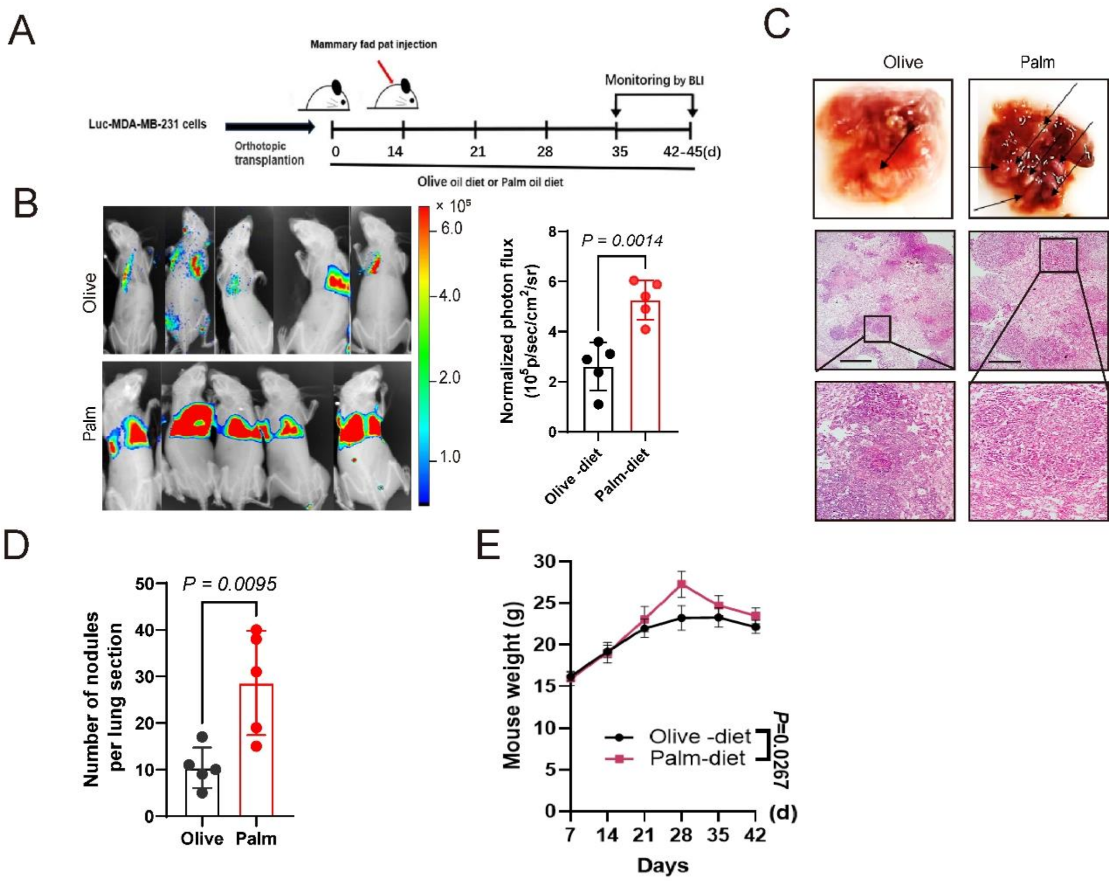

Figure S2 (Related to Figure1). Dietary palmitic acid promotes metastasis in breast cancers orthotopic transplantation model. **A)** Treatment plan for a high-fat diet supplemented with either palm oil (PA) or olive oil (OA) in an orthotopic transplantation model. **B)** Bioluminescent images (BLI), quantification of lung metastasis tumours generated from mice injected with luc-MDA-MB-231cells via orthotopic transplantation on the 14th day (n=5 mice in each group). **C)** Representative images of H&E staining (n=5 mice in each group). **(D, E)** The number of lung metastatic nodules **(D)** and weight **(E)** was counted and statistically analyzed. (n=5 mice in each group). *P* values were determined by the unpaired Student's t test (B, D), two-way analysis of variance analysis (E).

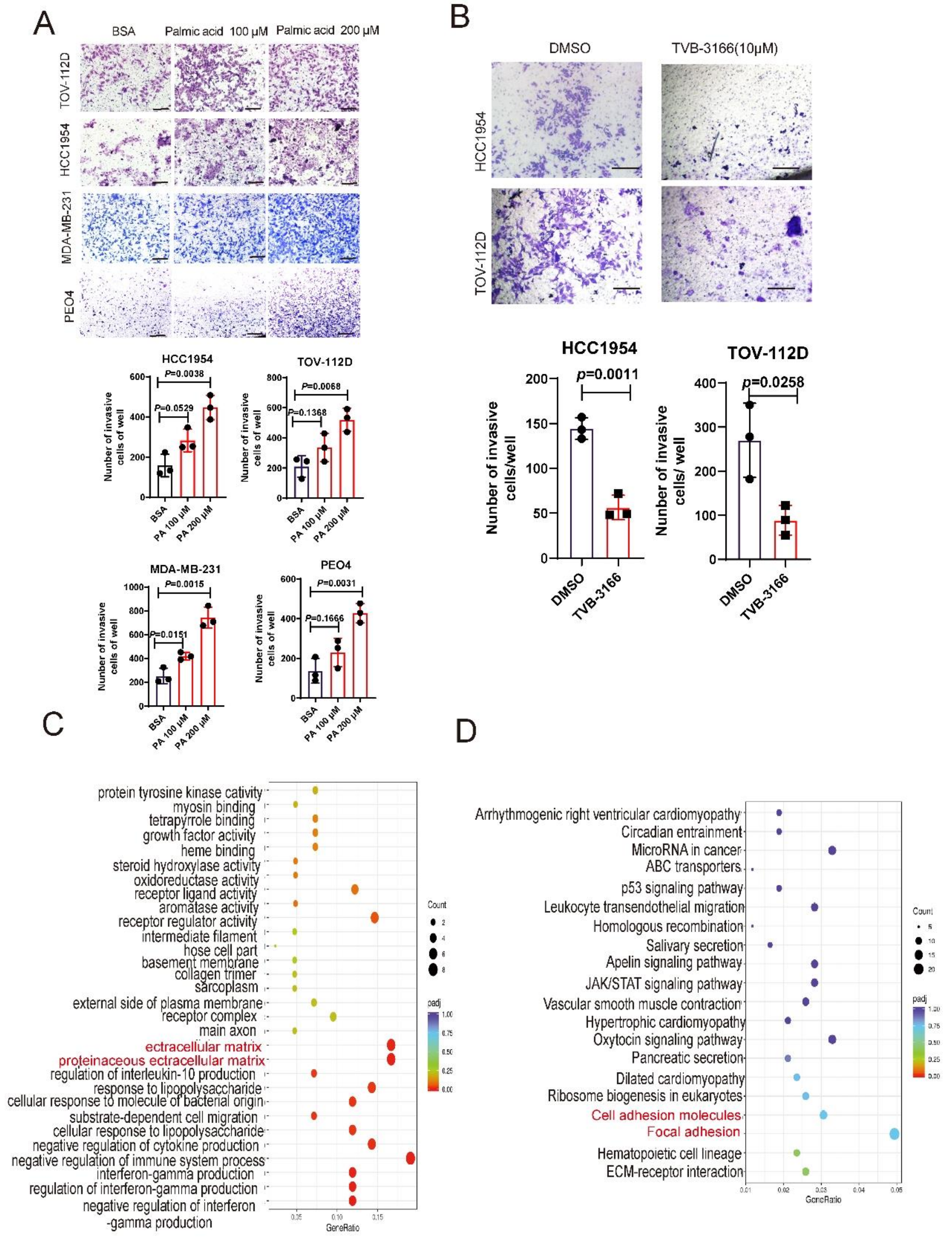

Figure S3 (Related to Figure1). Palmitic acid treatment enhancing ovarian cancer invasion and metastasis. A) The invasive capacity of TOV-112D, HCC1954, MDA-MB-231 and PEO4 cells was assessed using the trans-well chamber after treatment with vehicle (BSA) or palmitic acid (100 or 200 μM) for 16 h. *P* values were determined by

one-way ANOVA with Tukey's multiple comparison test (n=3). **B)** The cell invasion ability of TOV-112D and HCC1954 cells was evaluated using the trans-well chamber after treatment with vehicle (DMSO) or TVB-3166 (10 μM) for 24 h. *P* values were determined by the two-tailed Student's t test (n=3). Scale bar: 100 μm.

C, D) GO and KEGG analysis conducted on differentially expressed mRNA in TOV-112D cells treated with palmitic acid (100 μM, 24 h) compared to BSA treatment control.

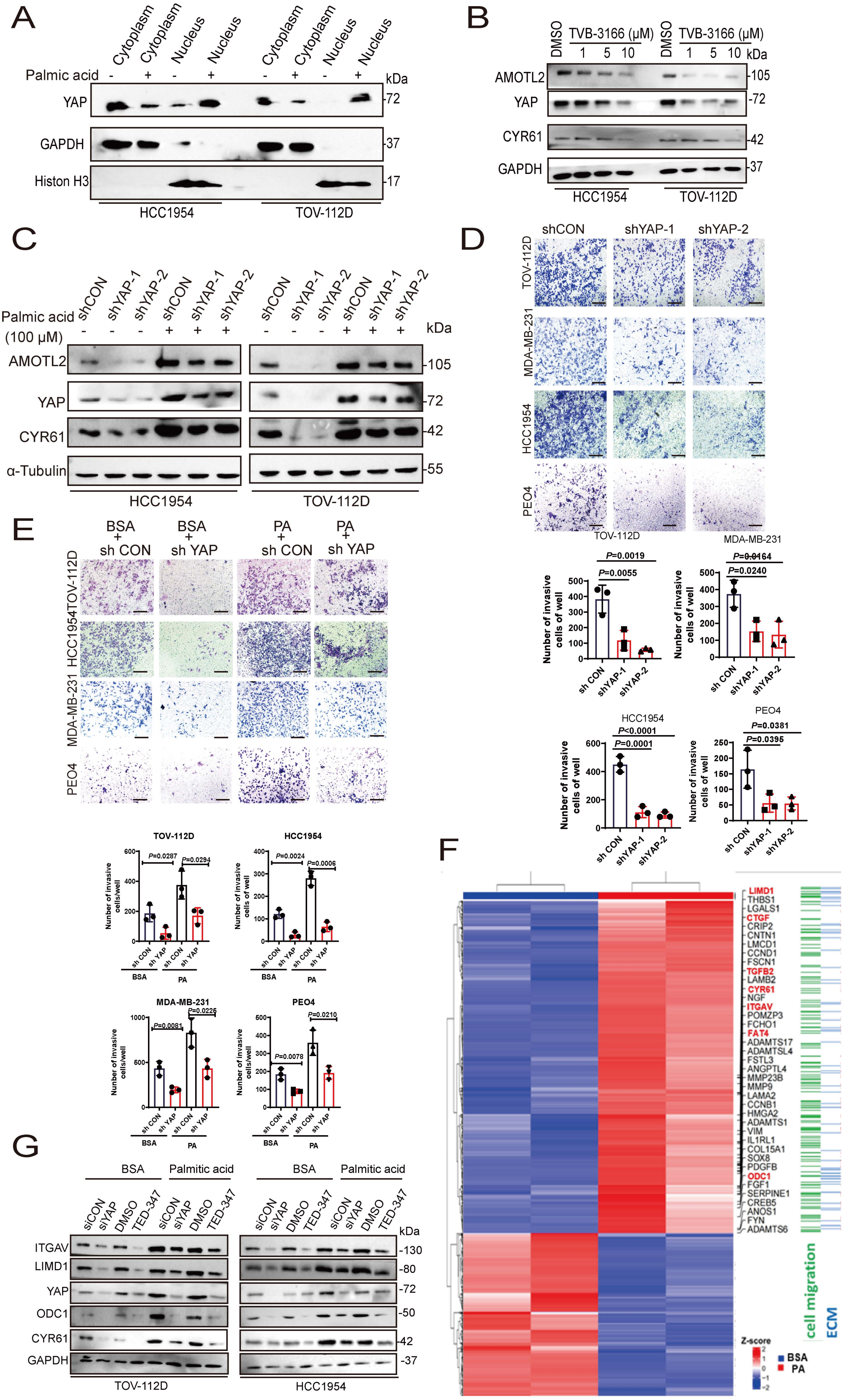
A
B
C
D
E
F
G
Palmic acid
YAP
GAPDH
Histon H3
HCC1954
TOV-112D
AMOTL2
CYR61
TVB-3166 (μM)
DMSO
shCON
shYAP-1
shYAP-2
α-Tubulin
MDA-MB-231
PEO4
BSA
PA
sh CON
sh YAP
ITGAV
LIMD1
ODC1
siCON
siYAP
TED-347
cell migration
ECM
Z-score

Figure S4 (Related to Figure 1). Palmitic acid promotes cancer metastasis in part through YAP signaling. A) HCC1954 and TOV-112D cells were treated with palmitic acid and subjected to immunoblotting (IB) analysis to determine the levels of YAP protein in the nucleus and cytoplasm. The experiment was conducted in triplicate. **B)** IB was performed to analyse the expression levels of AMOTL2, CYR61 and YAP with GAPDH as a loading control, in HCC1954 and TOV-112D cells stimulated with different concentrations of TVB-3166 (1, 5, and 10 μM) for 24 h. **C)** YAP-depleted HCC1954 and TOV-112D cells were stimulated with palmitic acid at various concentrations for 24 h. IB analysis was performed to assess the expression levels of AMOTL2, CYR61, and YAP. **D)** The invasive capacity of YAP-depleted TOV-112D, HCC1954, MDA-MB-231 and PEO4 cells. *P* values were determined by one-way ANOVA with Tukey's multiple comparison test (n=3). *P* values were determined by one-way ANOVA with Tukey's multiple comparison test (n=3). **E)** The invasion capability of TOV-112D, HCC1954, MDA-MB-231 and PEO4 cells with stable knockdown of YAP was evaluated using the trans-well chamber after treatment with vehicle (BSA) or palmitic acid (200 μM) for 16 h. *P* values were determined by one-way ANOVA with Tukey's multiple comparison test (n=3). Scale bar: 100 μm. Data are representative of three independent experiments. **F)** The heatmap shows that upon treatment with palmitic acid of TOV-112D cells, genes related to ECM, cell migration, and Hippo/YAP pathway were enriched compared to the control BSA treatment. **G)** HCC1954 and TOV-112D cells were treated with TEAD inhibitor TED-347 10 μM for 24 h, or transfected with siRNAs targeting YAP for 72h. Cells were then treated with

100 μM BSA or Palmitic acid for 24 h and subjected to IB analysis to evaluate the protein levels of ITGAV, LIMD1, ODC1, CYR61, YAP. GAPDH was used a loading control. Representative of two independent results was shown.

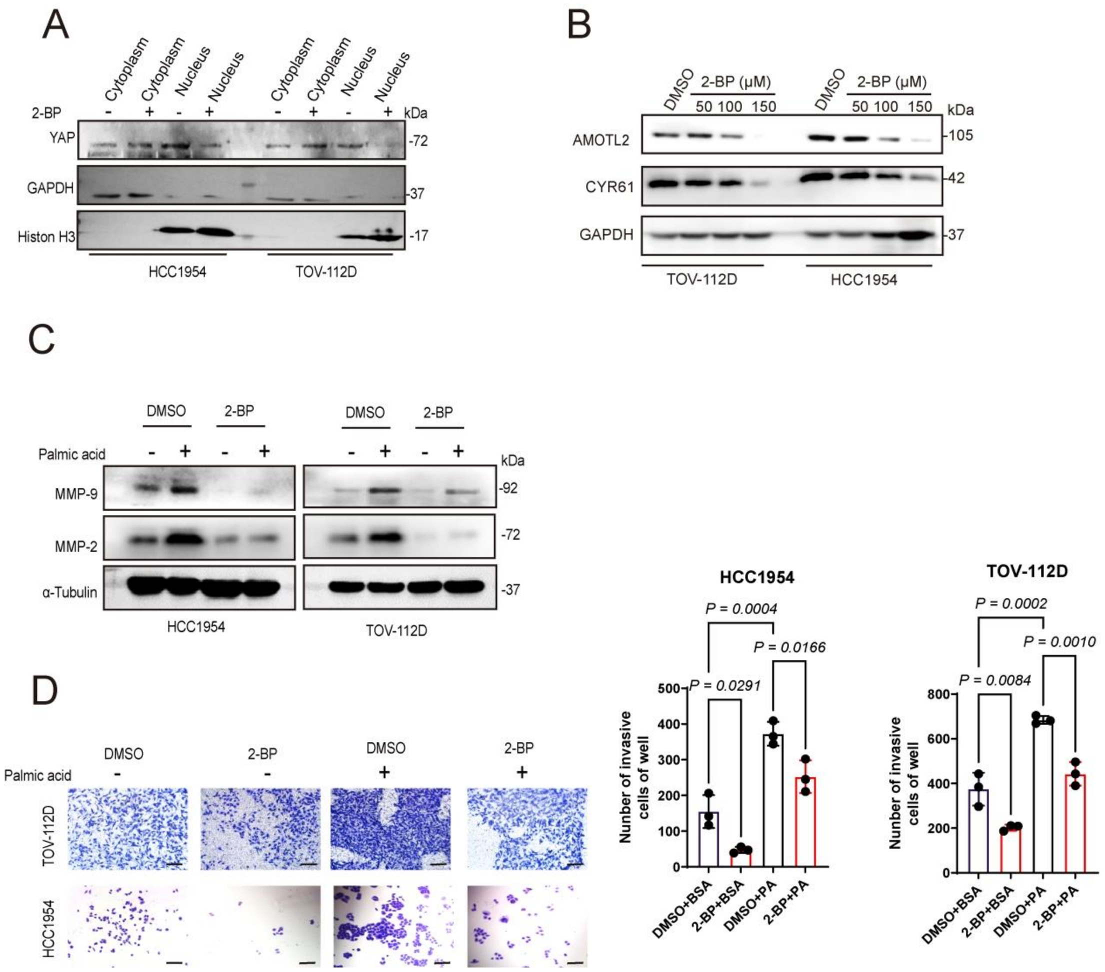

Figure S5 (Related to Figure2). ZDHHC15 acts as a positive regulator of YAP activity. A) HCC1954 and TOV-112D cells were treated with 2-BP (100 μM) for 24h and subjected to immunoblotting (IB) analysis to determine the levels of YAP protein in the nucleus and cytoplasm. The experiment was conducted in triplicate. **B)** IB was performed to analyse the expression levels of AMOTL2, CYR61 and YAP with GAPDH as a loading control, in HCC1954 and TOV-112D cells stimulated with different concentrations of 2-BP (50, 100, and 150 μM) for 24 h. **C)** HCC1954 and

TOV-112D cells were stimulated with palmitic acid 100 μM for 24 h and treated by DMSO or 2-BP 50 μM for 16 h. IB analysis was performed to assess the expression levels of MMP-2 and MMP-9. **D)** The invasive capacity of TOV-112D and HCC195 cells were stimulated with palmitic acid 100 μ M for 24 h and treated by DMSO or 2-BP 50 μM for 16 h. *P* values were determined by one-way ANOVA with Tukey's multiple comparison test (n=3). Scale bar: 100 μm. GAPDH was used a loading control. Data are representative of three independent experiments.

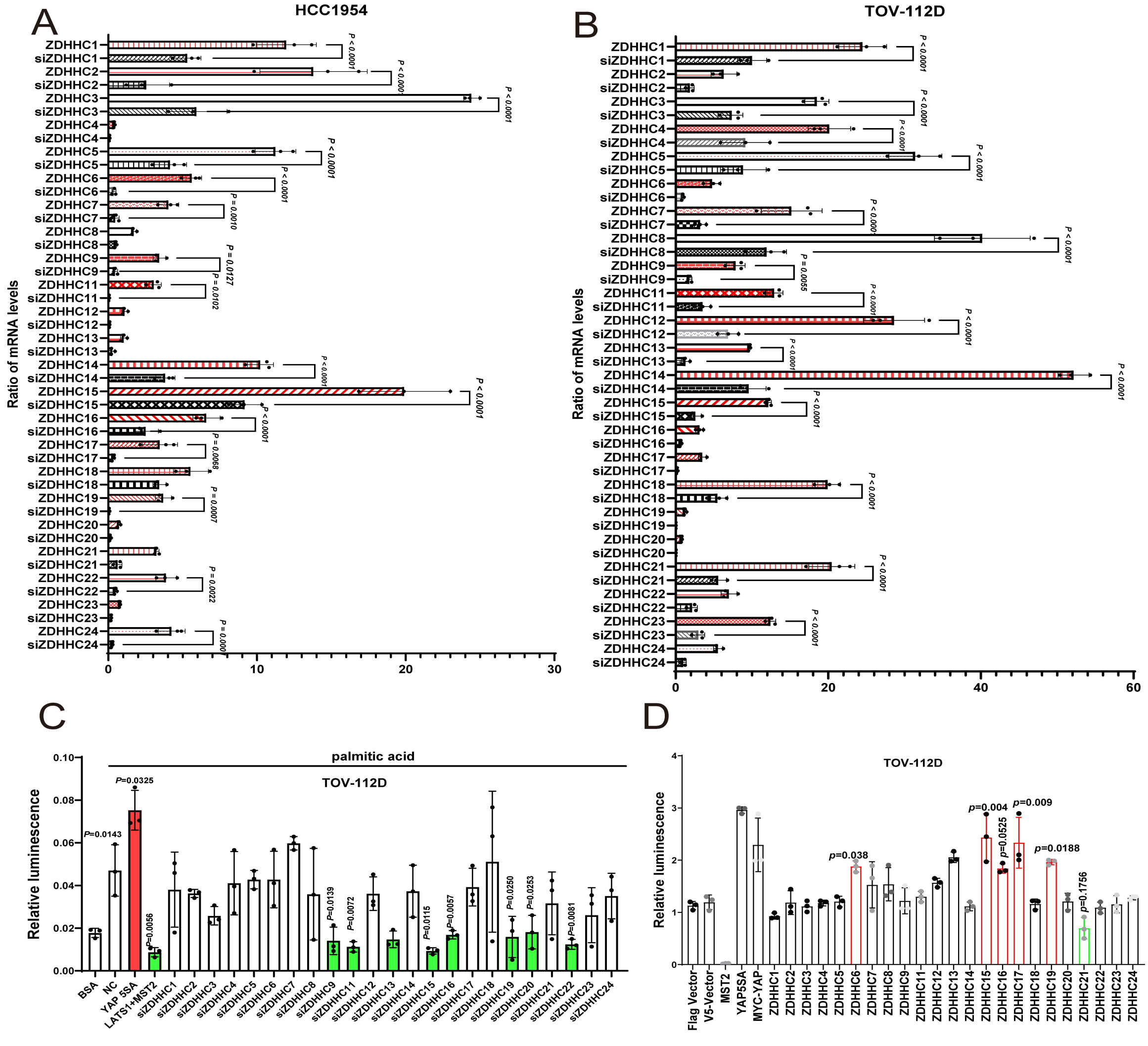

Figure S6 (Related to Figure 2). (A, B) RT-qPCR detection of siRNA knockdown efficiency. *P* values were determined by the two-tailed Student's t test (n=3). **C)**

TEAD luciferase reporter activity was assessed in TOV-112D cells following transfection with siRNAs targeting ZDHHC1-24 respectively or nontargeting control (NC), or MST2/LATS1 or vector control, along with the 8xGTIIC-luciferase reporter and pRL-TK Renilla luciferase, with Renilla luciferase serving as an internal control. Cells were then treated with palmitic acid (PA, 100 μM) for 36 h. *P* values were determined by one-way ANOVA with Tukey's multiple comparison test (n=3). **D)** TOV-112D cells following transfection with V5 or Flag–tagged-ZDHHC1-24 expression constructs, or YAP5SA or MST2/LATS1 or vector control, along with the 8xGTIIC-luciferase reporter and pRL-TK Renilla luciferase. *P* values were determined by one-way ANOVA with Tukey's multiple comparison test (n=3).

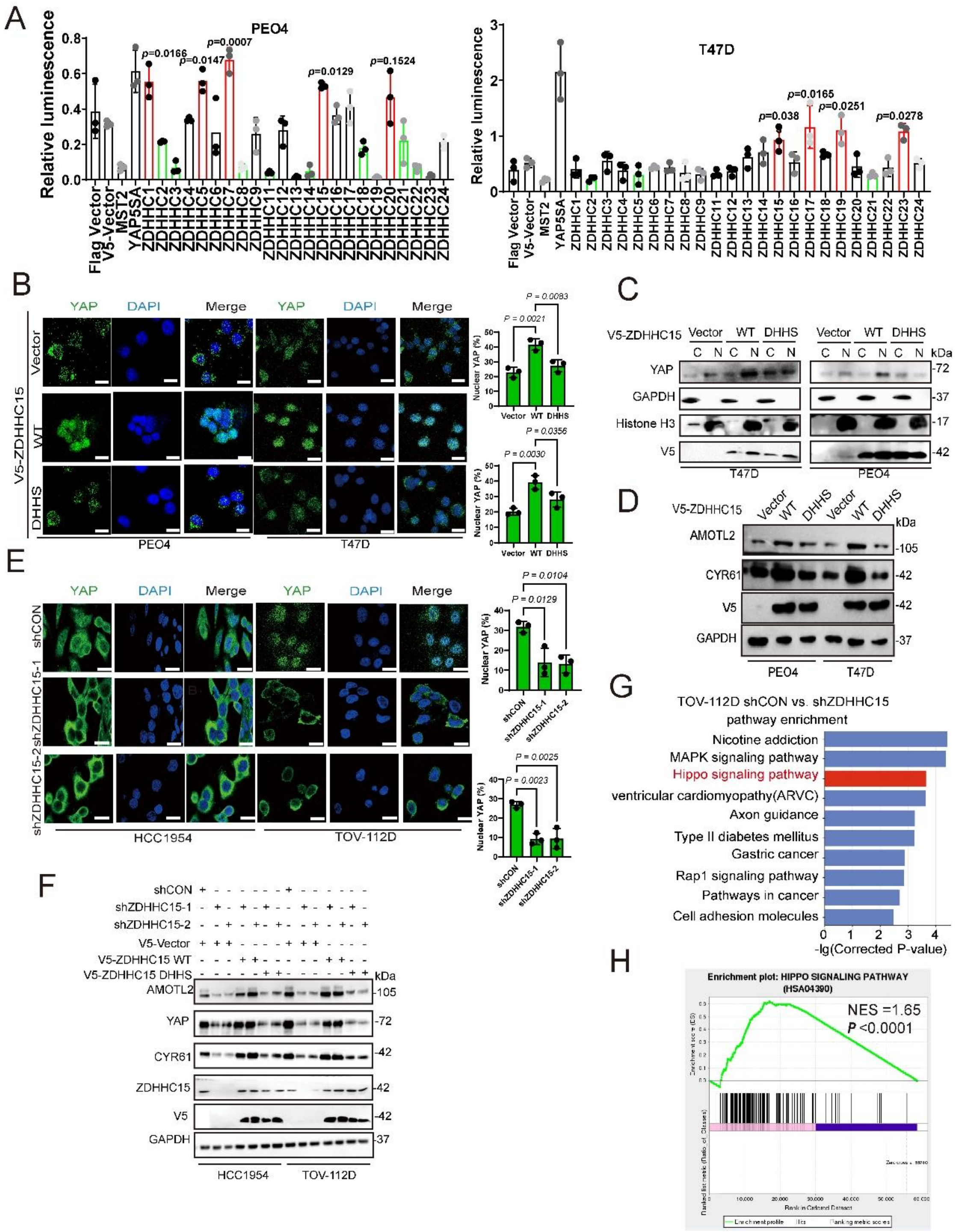

Figure S7 (Related to Figure 2). ZDHHC15 regulates YAP transcriptional activity.

A) TEAD luciferase reporter activity was assessed in T47D, and PEO4 cells following transfection with the V5 or Flag–tagged-ZDHHC1-24 expression constructs, or YAP5SA or MST2/LATS1 or vector control, along with the 8xGTIIC-luciferase reporter and pRL-TK Renilla luciferase, with Renilla luciferase serving as an internal

control. Data were normalized to the vector control. *P* values were determined by one-way ANOVA with Tukey's multiple comparison test (n=3). **B)** Immunofluorescence staining of YAP (green) and DAPI (blue). was performed on T47D and PEO4 cells transfected with V5-ZDHHC15 WT and its DHHS variant. *P* values were determined by one-way ANOVA with Tukey's multiple comparison test (n=3). Scale bars: 25 μm. **C)** IB analysis of YAP protein levels in the nucleus and cytoplasm of T47D and PEO4 cells transfected with V5-ZDHHC15 WT and DHHS mutant constructs. **D)** IB analysis was conducted to evaluate the levels of AMOTL2 and CYR61 in T47D and PEO4 cells following transfection with V5-ZDHHC15 WT and DHHS variant. **E)** Confocal analysis was employed to assess YAP nuclear localization in ZDHHC15-depleted HCC1954 and TOV-112D cells. *P* values were determined by one-way ANOVA with Tukey's multiple comparison test (n=3). Scale bar: 25 μm. **F)** HCC1954 and TOV-112D cells in which ZDHHC15 was stably depleted, were infected with V5-ZDHHC15 WT or DHHS variant. IB analysis was performed to evaluate YAP level and target gene expression. **G)** KEGG pathway analysis of differential expressed mRNA transcripts in ZDHHC15-depleted TOV-112D cells and their respective control cells. **H)** Gene set enrichment analysis revealed enrichment in Hippo pathway.

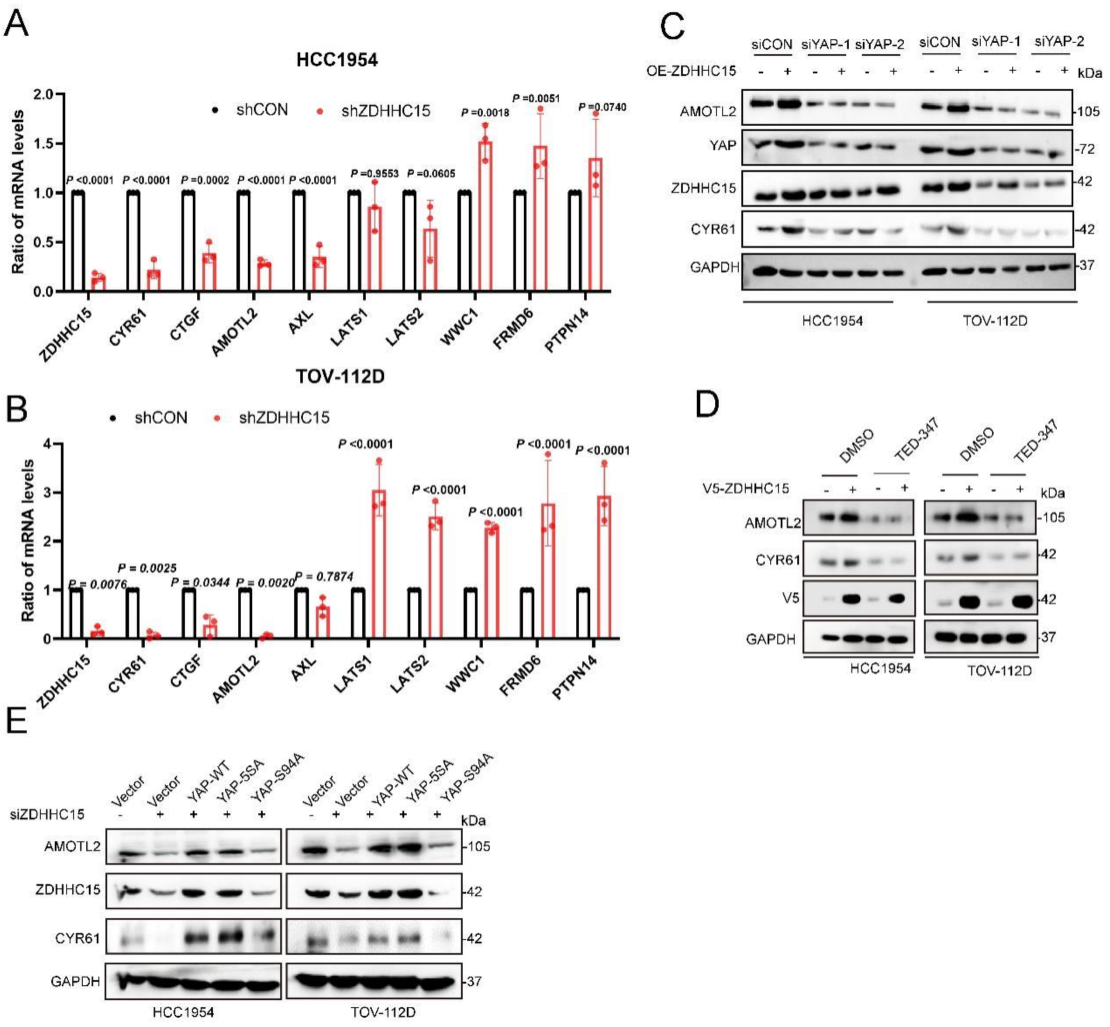

Figure S8 (Related to Figure 2). ZDHHC15 modulates YAP activity. A, B) RT-qPCR analysis of the mRNA levels of Hippo/YAP pathway genes in ZDHHC15-depleted HCC1954 cells (A) or TOV-112D cells (B). Data were normalized to GAPDH. *P* values were assessed by one-way ANOVA followed by Tukey's multiple-comparison test (n=3). **C)** Immunoblotting (IB) analysis showing the decrease in YAP target gene expression levels in ZDHHC15-overexpressed-HCC1954 and TOV-112D cells transfected with non-target siRNA control or siRNAs targeting human YAP. **D)** IB analysis of AMOTL2 and CYR61 levels in ZDHHC15-overexpressed HCC1954 and TOV-112D cells treated with TED-347 10 μM for 24 h. **E)** IB analysis of AMOTL2 and CYR61 levels in ZDHHC15-depleted HCC1954 and TOV-112D cells

transfected with the vector control or plasmids encoding YAP-WT, YAP-5SA, and YAP-S94A respectively. GAPDH was used a loading control. Data are representative of three independent experiments.

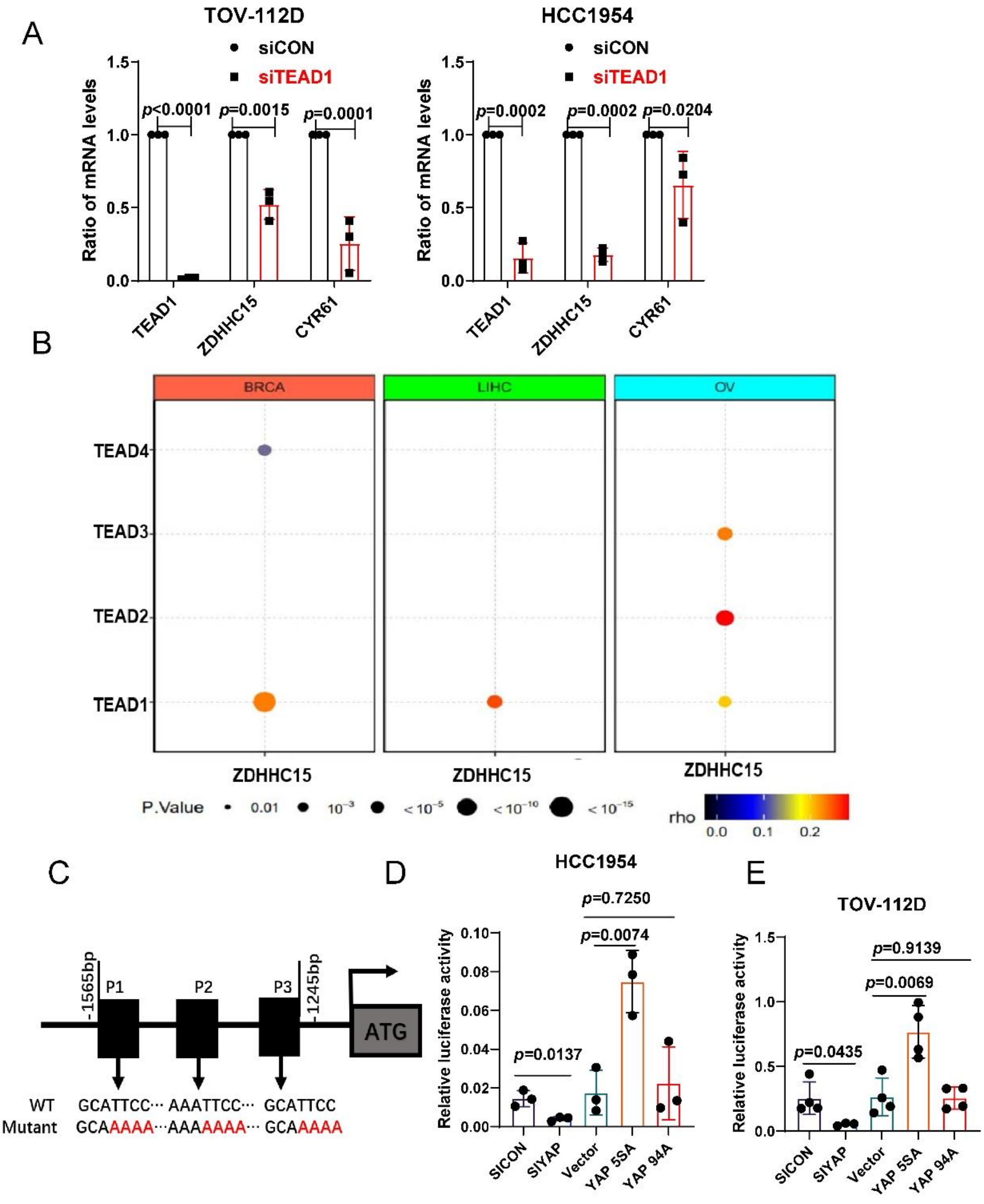

Figure S9 (Related to Figure 3). YAP/TEAD1 controls transcription expression of ZDHHC15. A) RT-qPCR was performed to detect the levels of TEAD1, ZDHHC15, and CYR61 mRNA in HCC1954 and TOV-112D cells transfected with non-target siRNA or siRNA targeting TEAD1 for 48 h. The experiment was performed independently three times, and the results were normalized to GAPDH, with control set as 1. *P* values were determined by unpaired Student's t test (n=3). **B)** Query of the TCGA database revealed a positive correlation between the expression of TEAD1 and ZDHHC15 in breast cancer (BRCA), liver cancer (LIHC), and ovarian cancer (OV) tissues. **C)** A sequence diagram illustrating the binding site sequence mutant between TEAD1 and the ZDHHC15 promoter. **D, E)** Luciferase reporter assays were performed using the ZDHHC15 promoter in HCC1954 **(D)** and TOV-112D **(E)** cells transfected with the vector control or plasmids encoding YAP-WT, YAP-5SA, and YAP-S94A respectively. *P* values were assessed by one-way ANOVA followed by Tukey's multiple-comparison test (n=3)

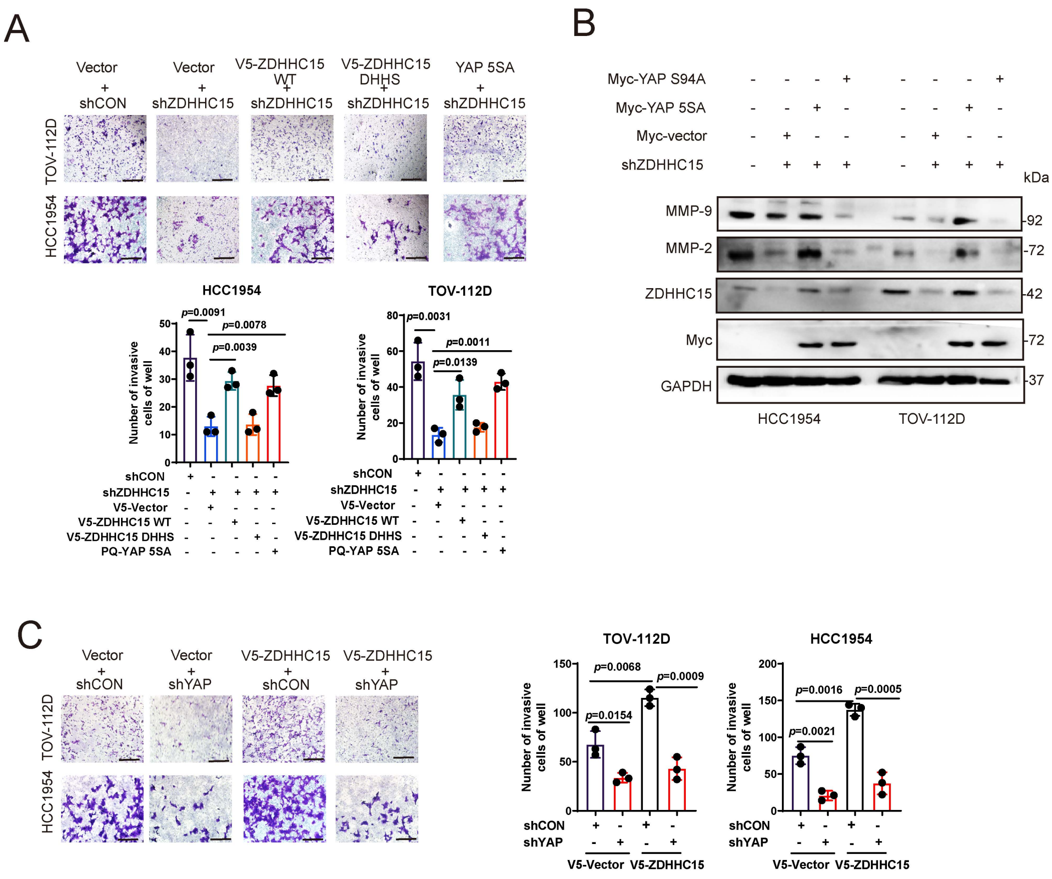

Figure S10 (Related to Figure 4). ZDHHC15 promotes tumor metastasis. A)

TOV-112D and HCC1954 cells with stable knockdown of ZDHHC15 were transfected with V5-ZDHHC15 WT, DHHS, or PQ-YAP 5SA or vector control. The cell invasion ability was evaluated using the trans-well chamber. *P* values were assessed by one-way ANOVA followed by Tukey's multiple-comparison test (n=3). Scale bar: 100 μm. **B)** HCC1954 and TOV-112D cells with stable knockdown of ZDHHC15 were transfected with the vector control or plasmids encoding Myc-tagged YAP 5SA and Myc-tagged YAP S94A. The expression levels of MMP-2, MMP-9, and ZDHHC15 were measured using immunoblotting (IB). GAPDH was used a loading control. Data are representative of three independent experiments. **C)** TOV-112D and HCC1954 cells with stable knockdown of YAP were transfected with V5-ZDHHC15. The cell invasion ability was evaluated using the transwell chamber. *P* values were assessed by one-way ANOVA followed by Tukey's multiple-comparison test (n=3). Scale bar: 100 μm.

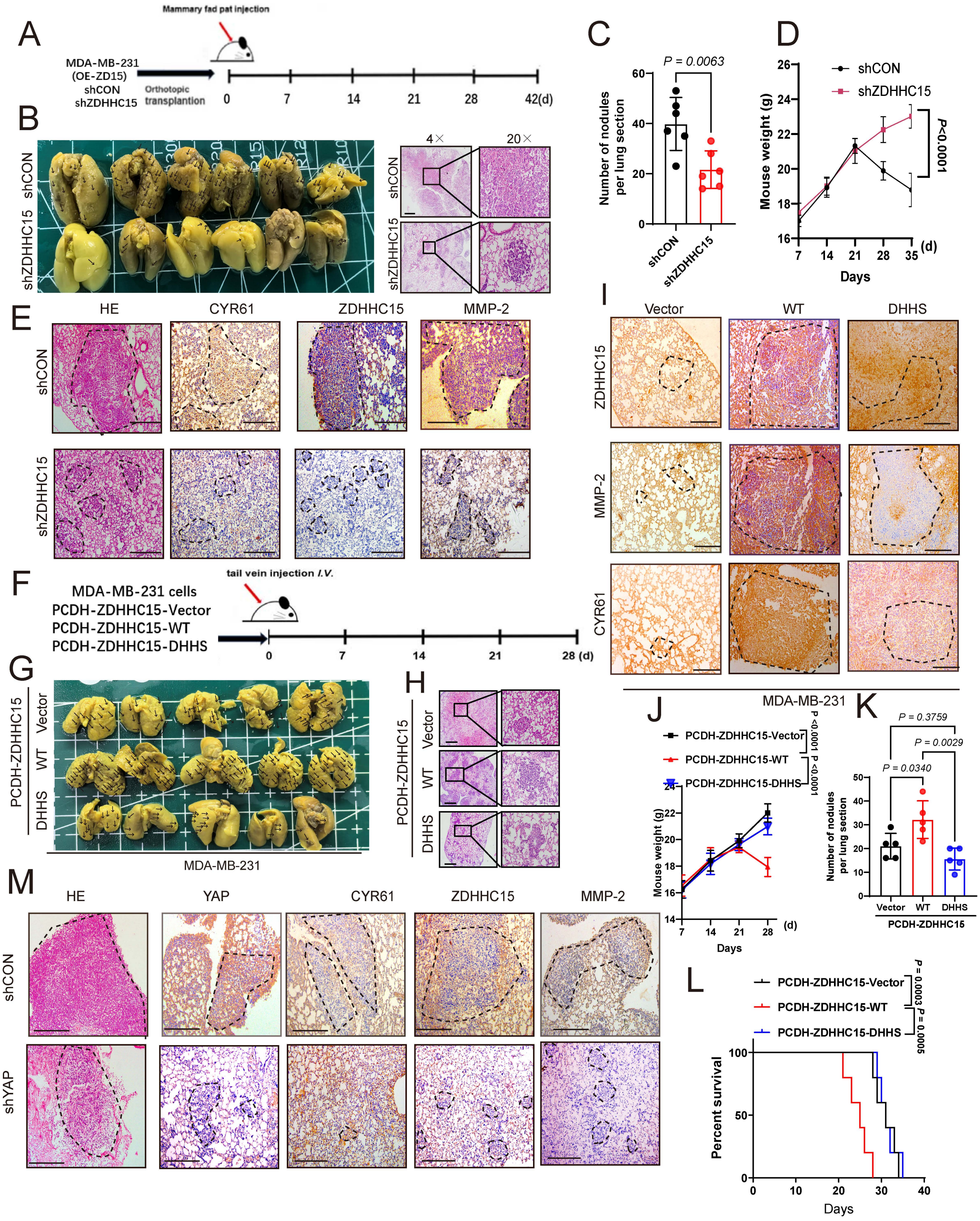

Figure S11 (Related to Figure 4). ZDHHC15 promotes metastasis in breast cancer. **A)** Experimental protocol for ZDHHC15- depleted MDA-MB-231 cells to promote tumor metastasis in an orthotopic transplantation model. (n=6 mice in each group). **B)** ZDHHC15-depleted MDA-MB-231(OE-ZDHHC15) cells subjected to athymic nude mice xenograft in mice No. 4 nipple fat pad below. After 6 weeks mice were euthanized,

and the brightfield lung images of each group are shown (n=6 mice in each group). **(C, D)** The number of lung metastatic nodules **(C)** and weigh **(D)** was counted and statistically analyzed. (n=6 mice in each group). **(E)** Representative images of H&E staining and immunohistochemical staining of ZDHHC15, MMP-2 and CYR61 performed on paraffin-embedded xenograft tumor tissues. Scale bar: 100 μm. **(F, G)** MDA-MB-231cells were overexpressed with Vector, ZDHHC15-WT or ZDHHC15-DHHS constructs, then subjected to athymic nude mice xenograft through tail vein injection. After 5 weeks mice were euthanized, and the brightfield lung images and representative images of H&E staining of each group are shown (n=5 mice in each group). **(J-L)** The weight **(J)**, the number of lung metastatic nodules **(K)** and survival rate **(L)** was counted and statistically analyzed. (n=5 mice in each group) **(I)** Representative images of H&E staining and immunohistochemical staining of ZDHHC15, MMP-2 and CYR61 performed on paraffin-embedded xenograft tumor tissues. Scale bar: 100 μm. **M)** Representative immunohistochemical staining (TOV-112D group) of ZDHHC15, MMP-2, CYR61, and YAP performed on paraffin-embedded xenograft tumor tissues. Scale bar: 100 μm. *P* values were determined by the one-way ANOVA with Tukey's multiple comparison test (C, K), two-way analysis of variance analysis (D, J), Log-rank Mantel-Cox test (L).

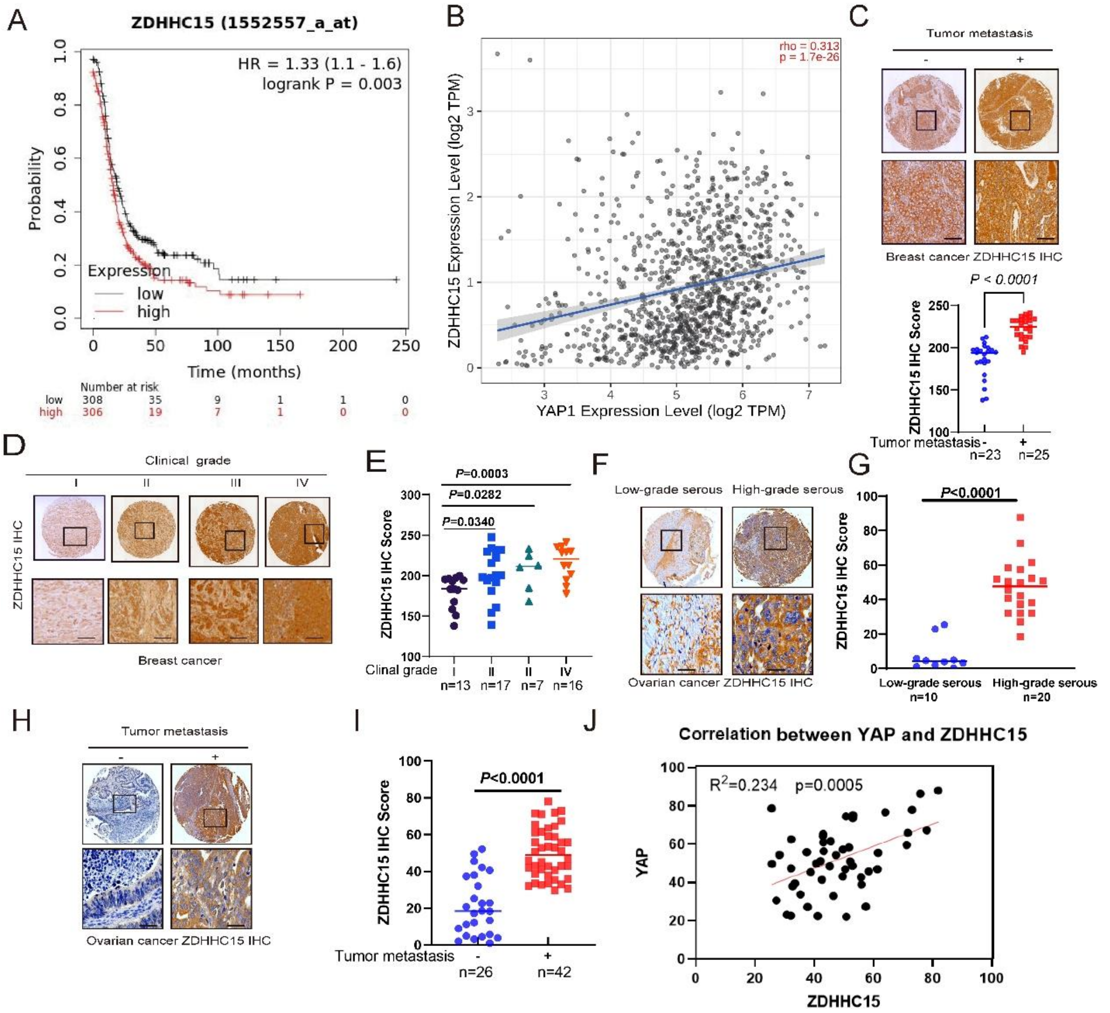

Figure S12 (Related to Figure 4). ZDHHC15 is a prognostic factor in breast and ovarian cancers. A) Kaplan–Meier survival analysis of ovarian cancer samples from TCGA (n=614, *P*=0.003). **B)** The correlation between ZDHHC15 and YAP1 expression in breast cancer (TIMER 2.0, *P* <0.05). **C)** IHC staining of ZDHHC15 was performed on breast tumor samples with distinct metastatic abilities. IHC staining of ZDHHC15 (upper) and the corresponding IHC scores were analysed in breast cancer samples with varying metastatic abilities (lower). **(D, E)** IHC staining of ZDHHC15 **(D)** and the corresponding IHC scores **(E)** were analyzed in different clinical stages of breast tumors samples. *P* values were assessed by one-way ANOVA followed by Tukey's multiple-comparison test. Scale bars: 100 μm. **(F, G)** IHC staining of ZDHHC15 **(F)** and the corresponding IHC scores **(G)** were analysed in different clinical stages of ovarian cancer samples. *P* values were determined by unpaired Student's t test. Scale

bars: 100 µm. **(H, I)** IHC staining of ZDHHC15 **(H)** and the corresponding IHC scores **(I)** were analysed in ovarian cancer samples with varying metastatic abilities. *P* values were determined by unpaired Student's t test. Scale bars: 100 µm. **(J)** The correlation between ZDHHC15 and YAP expression in ovarian cancer tissues. *P* values were the two tailed Pearson correlation.

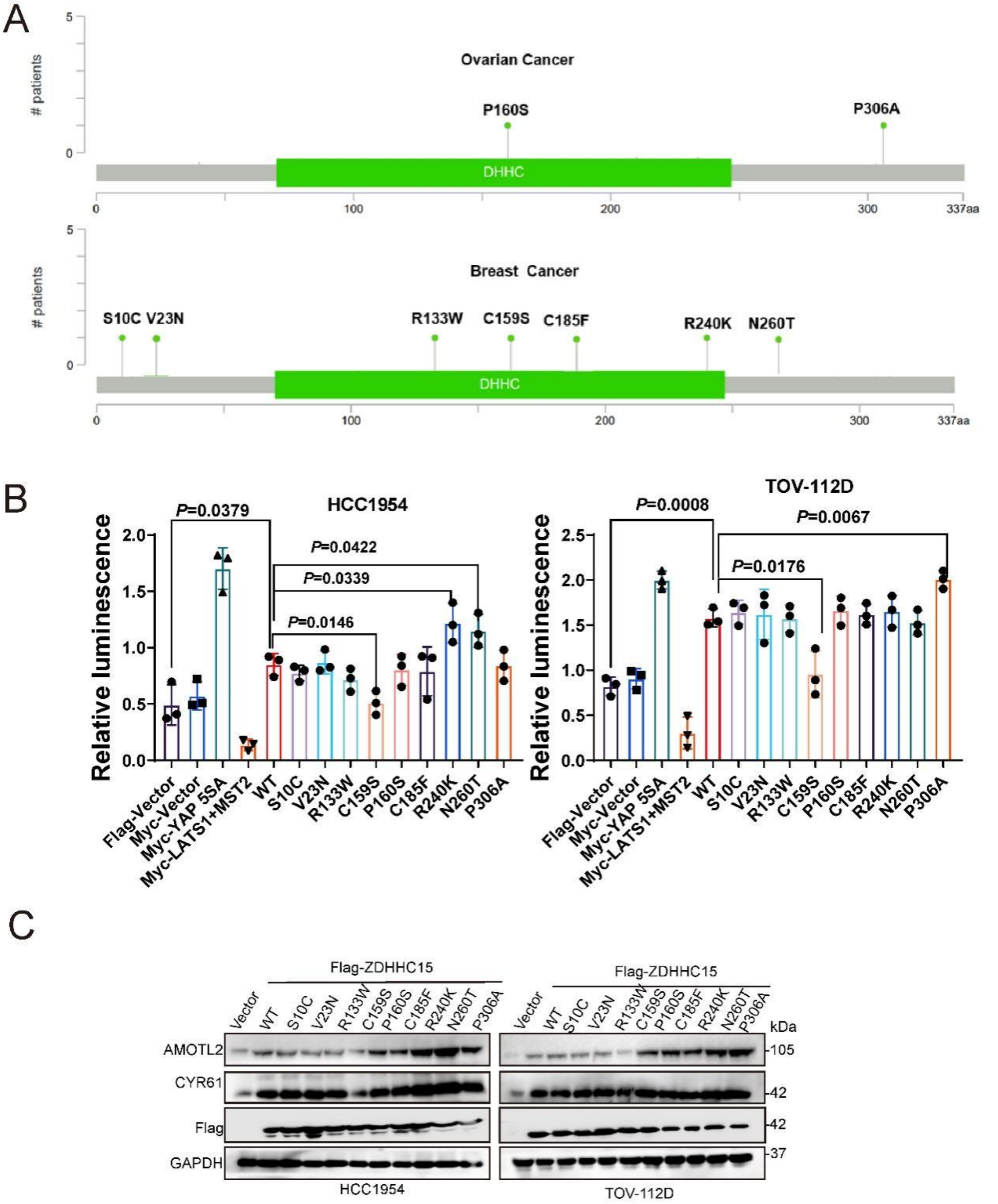

Figure S13 (Related to Figure 4). The effect of ZDHHC15 mutations on YAP activity. A) Schematic diagram of ZDHHC15 mutations in breast or ovarian cancer. **B)** HCC1954 and TOV-112D cells were transfected with the 8xGTIIC-luciferase reporter

along with Flag-ZDHHC15 WT and indicated mutants. YAP 5SA and Myc-tagged LATS1/MST2 were utilized as positive and negative controls, respectively. *P* values were assessed by one-way ANOVA followed by Tukey's multiple-comparison test (n=3). **C)** IB analysis was conducted to evaluate the levels of AMOTL2 and CYR61 in HCC1954 and TOV-112D cells following transfection with Flag-ZDHHC15 WT and mutants. GAPDH as a loading control Data are representative of three independent experiments.

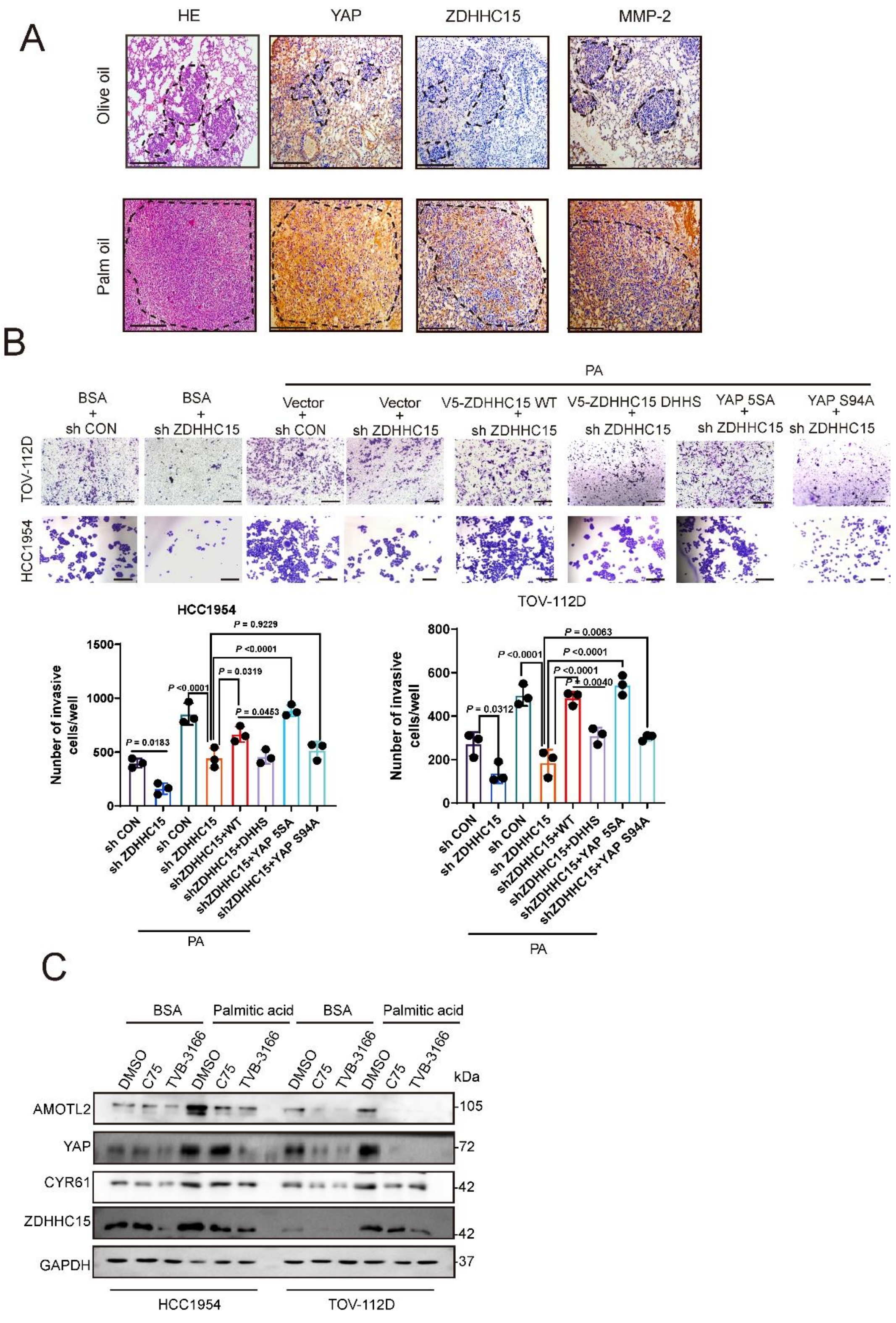

Figure S14 (Related to Figure 5). ZDHHC15-YAP loop in tumor metastasis. A) Immunohistochemical (IHC) staining of ZDHHC15, MMP-2 and YAP in paraffin-embedded xenograft tumor tissues (TOV-112D cells) from the indicated groups. **B)**

TOV-112D and HCC1954 cells with stable knockdown of ZDHHC15 were transfected with V5-ZDHHC15 WT, DHHS, Myc-YAP 5SA or Myc-YAP S94A after treatment with vehicle (BSA) or palmitic acid (200 μM) for 16 h. The cell invasion ability was evaluated using the trans-well chamber assay. Data are presented as the mean ± SD. *P* values were assessed by one-way ANOVA followed by Tukey's multiple-comparison test (n=3). Scale bars: 100 μm. **(C)** HCC1954 and TOV-112D cells were treated with FASN inhibitor C75 25 μM or TVB-3166 10 μM for 24 h. Then treated with 100 μM BSA or Palmitic acid for 24 h and subjected to IB analysis to evaluate the protein levels of AMOTL2, CYR61, YAP and ZDHHC15. GAPDH as a loading control. Data are representative of three independent experiments.

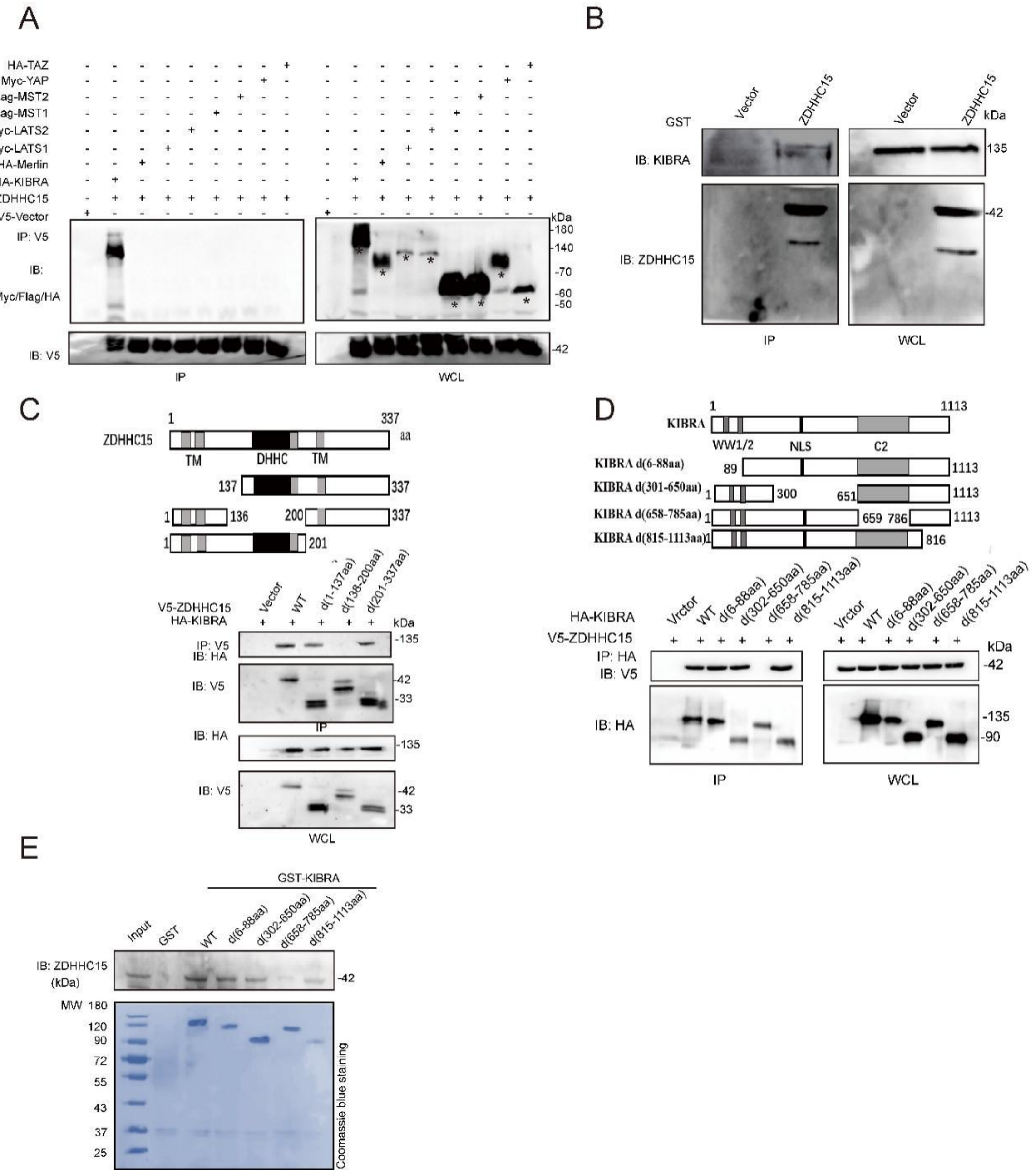

Figure S15 (Related to Figure 6) ZDHHC15 Interacts with KIBRA. A) HEK293T cells were transfected with V5-tagged ZDHHC15 or vector control, along with various constructs expressing the components of Hippo pathway respectively. Cell lysates were subjected to immunoprecipitation (IP) using anti-V5 antibodies. Immunoblotting (IB) was performed using antibodies against V5, Myc, HA, and Flag, respectively. **B)** GST pulldown assay determined the interaction between reconstituted GST fusion ZDHHC15 and KIBRA. **(C, D)** IP analysis of the domains mediating the interaction between ZDHHC15 and KIBRA. HEK293T cells were transfected various ZDHHC15 deletion mutants **(C)** or KIBRA deletion mutants **(D)**. **E)** GST fusion KIBRA and its deletion mutants were reconstituted and directly interacted with ZDHHC15 in a GST

pulldown assay. Data are representative of three independent experiments.

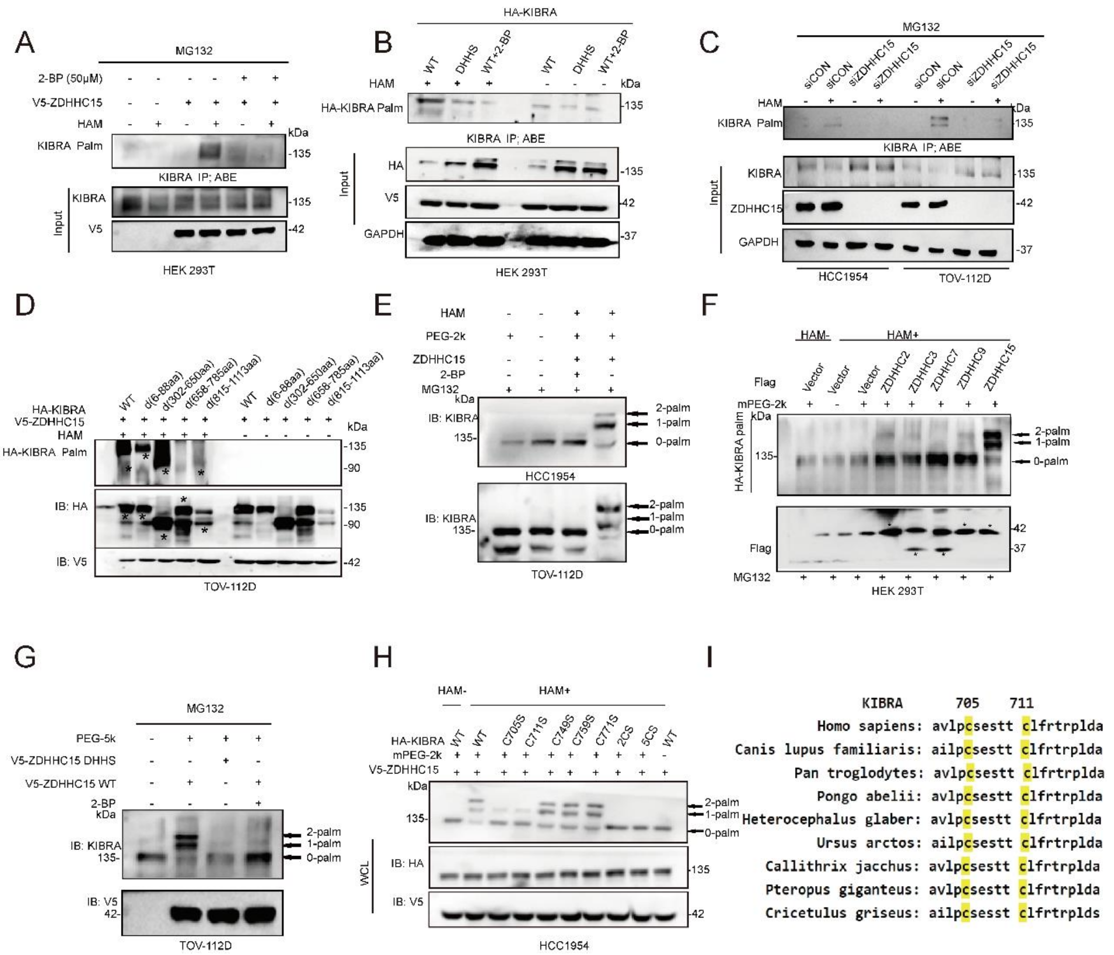

Figure S16 (related to Figure 6) ZDHHC15 controls KIBRA palmitoylation. A) ABE assay was performed to analyze KIBRA palmitoylation in HEK293T cells with ectopic expression of V5-ZDHHC15 and treatment with 2-BP at 50 μM for 24h. **B)** ABE assay was conducted to analyze exogenous KIBRA palmitoylation in HEK-293T cells with ectopic expression of HA-KIBRA, V5-ZDHHC15 WT, and DHHS mutant and treatment with 50 μM of 2-BP in the absence or presence of HAM. **C)** ABE assay was performed to analyze KIBRA palmitoylation in HCC1954 and TOV-112D cells in which ZDHHC15 was knocked down by siRNA. **D)** ABE analysis was conducted to evaluate exogenous KIBRA palmitoylation in TOV-112D cells expressing V5-ZDHHC15, HA-KIBRA, and its deletion mutants, with MG132 pretreatment. **E)** APE assays were utilized to analyze endogenous KIBRA palmitoylation in HCC1954 and TOV-112D cells upon ectopic expression of V5-ZDHHC15, and treatment with 50 μM of 2-BP in the absence or presence of HAM. The top two bands indicate palmitoylated KIBRA. **F)** APE assay was performed to assess KIBRA palmitoylation in HEK293T

cells with ectopic expression of Flag-ZDHHC2, Flag-ZDHHC3 Flag-ZDHHC7, Flag-ZDHHC9 and Flag-ZDHHC15 respectively, along with HA-KIBRA. MG132 pretreatment was applied for 4 h. The top two bands indicate palmitoylated KIBRA. **G)** APE assay was performed to analyze KIBRA palmitoylation in TOV-112D cells with ectopic expression of V5-ZDHHC15 WT or DHHS mutant. TOV-112D cells were pretreated with MG132 for 4 h and treated with 50 μM of 2-BP for 24 h. The top two bands indicate palmitoylated KIBRA. **H)** APE assay was performed to analyze KIBRA palmitoylation in HCC1954 cells with ectopic expression of HA-KIBRA WT, C705S, C711S, C759S, C759S, C771S, 2CS and 5CS respectively. The top two bands indicate the palmitoylated KIBRA. **I)** Evolutionarily conserved cysteine sites C705 and C711 of KIBRA are observed across several mammalian species. Data are representative of three independent experiments.

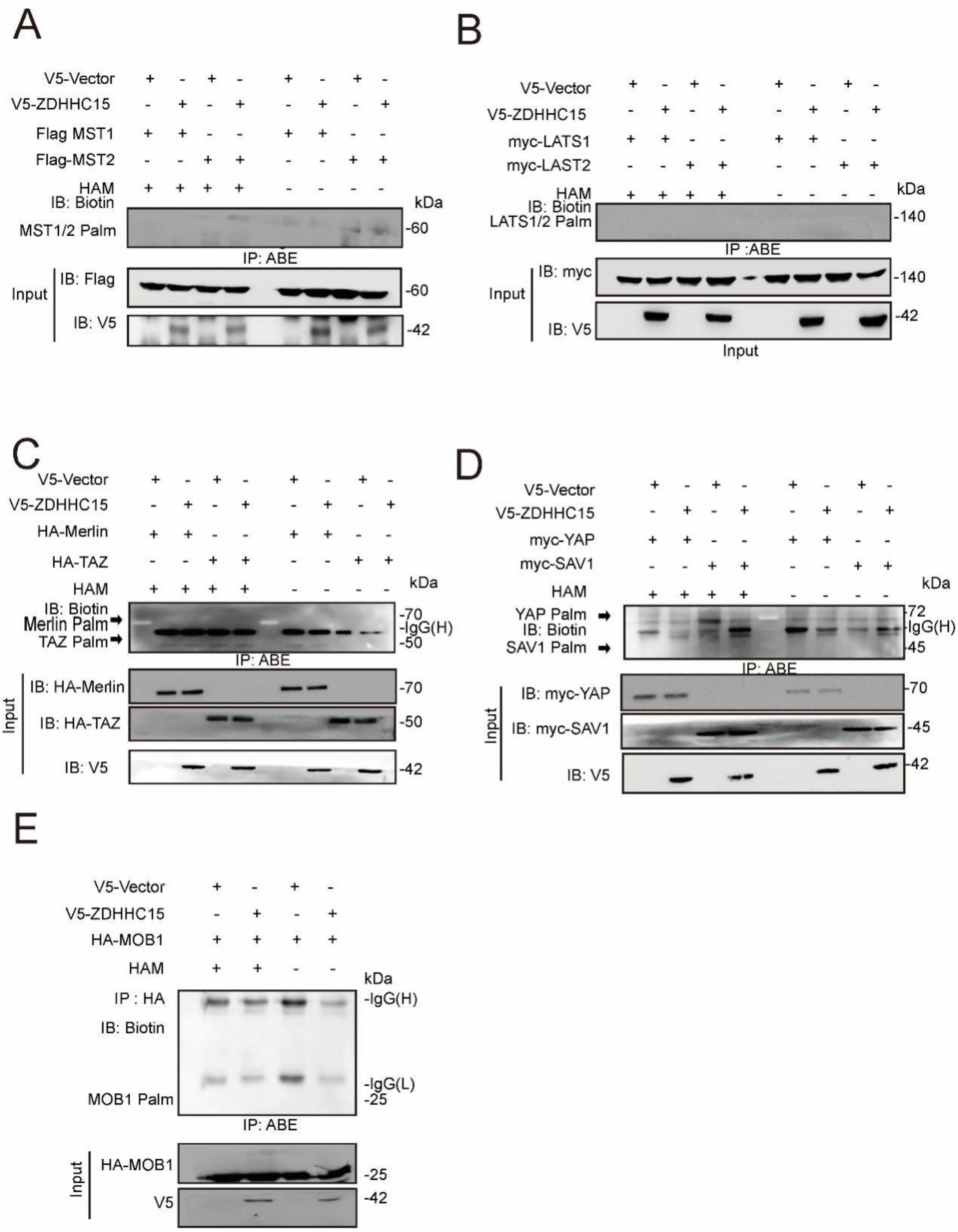

Figure S17 (Related to Figure. 6). ZDHHC15 cannot mediate the palmitoylation of core components of the Hippo pathway in addition to KIBRA. (A-E) ABE assay was performed to analyze the palmitoylation of exogenous MST1/2 **(A)**, LATS1/2 **(B)**,

Merlin/TAZ **(C)**, YAP/SAV1 **(D)**, and MOB1 **(E)** in TOV-112D cells with ectopic expression of V5-ZDHHC15 and the plasmids encoding several key components of Hippo/YAP pathway in the absence or presence of HAM. Data are representative of three independent experiments.

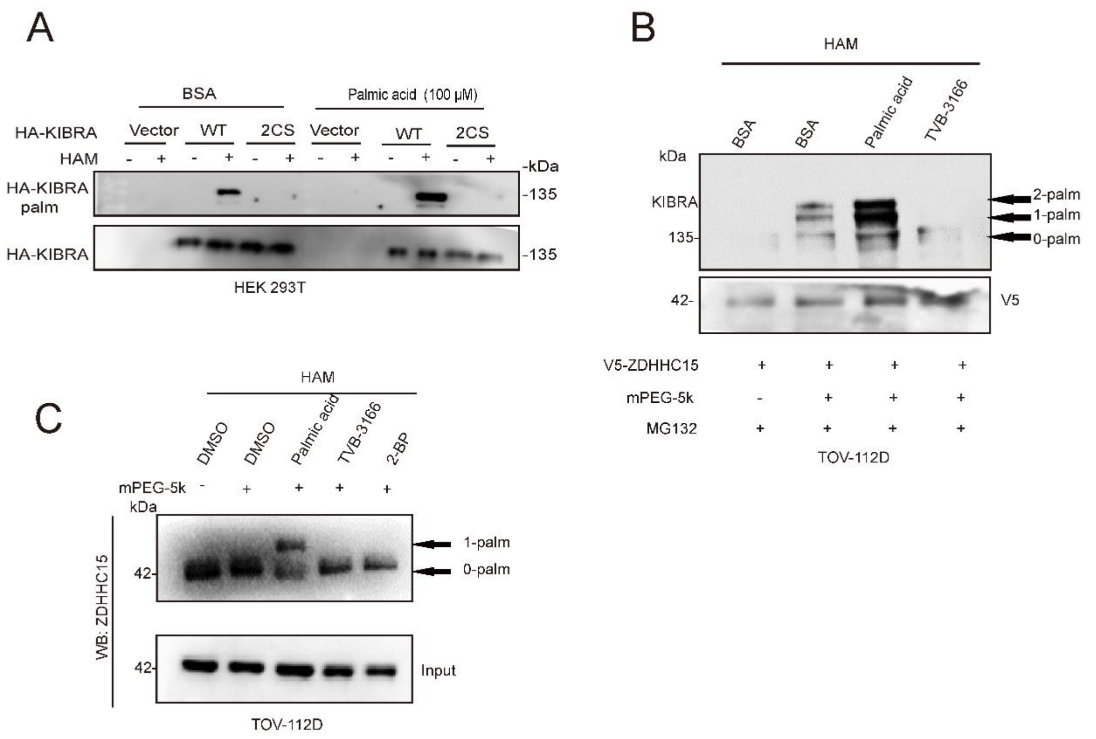

Figure S18 (related to Figure 6). Palmitic acid enhances KIBRA palmitoylation. A) ABE assay was conducted to analyze the palmitoylation of exogenous KIBRA in HEK293T cells expressing HA-KIBRA WT and HA-KIBRA 2CS respectively. Cells were treated with 100 μM palmitic acid for 24 h in the absence or presence of hydroxylamine (HAM). The experiments were independently repeated three times. **B)** APE assay was performed to analyse the palmitoylation of endogenous KIBRA in TOV-112D cells overexpressing V5-ZDHHC15 constructs. Cells were pretreated with MG132 for 4 h and subsequently treated with 100 μM palmitic acid or 20 μM TVB-3166 for 24 h. The top two bands Represent palmitoylated KIBRA, and lack of mPEG

was used as a negative control. **C)** APE assay was performed to analyse the palmitoylation of endogenous ZDHHC15 in TOV-112D cells. Cells were treated with palmitic acid (PA, 100 µM), or 20 µM TVB-3166 or 50 µM 2-BP for 24 h in the absence or presence of hydroxylamine (HAM). The top band represents palmitoylated ZDHHC15, and lack of mPEG was used as a negative control. Data are representative of three independent experiments.

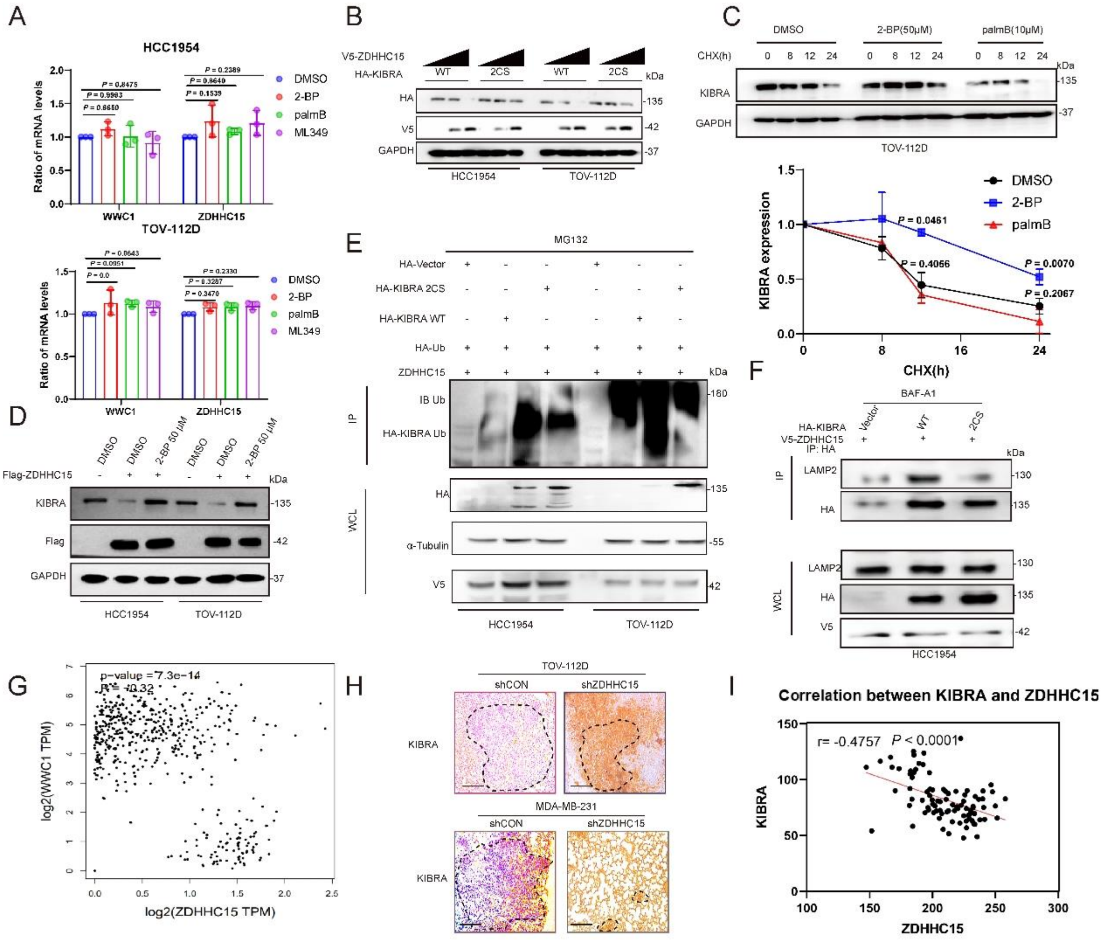

Figure S19. (Related to Figure 7) ZDHHC15 decreases the protein stability of KIBRA. A) RT-qPCR was performed to assess the expression levels of WWC1 and ZDHHC15 in HCC1954 and TOV-112D cells treated with 2-BP (50 µM), ML349 (20 µM), and palmB (1 µM) for 16 h. *P* values were assessed by one-way ANOVA followed by Tukey's multiple-comparison test (n=3). **B)** HCC1954 and TOV-112D cells were

transfected with varying amounts (0, 1, 2 μg) of V5-ZDHHC15 along with HA-tagged-KIBRA-WT or 2CS constructs. The protein levels of HA-KIBRA were analyzed by immunoblotting (IB). **C)** TOV-112D cells were treated with 2-BP (50 μM) and palmB (10 μM) for 8 h in the presence of cycloheximide (CHX). IB were conducted to analyze the expression levels of KIBRA protein at different time points. *P* values were assessed by one-way ANOVA followed by Tukey's multiple-comparison test (n=3). **D)** HCC1954 and TOV-112D cells were transfected with V5-ZDHHC15 and treated with 2-BP (25 μM) and 2-BP (50 μM). IB were performed to assess the protein expression levels of KIBRA. **E)** HCC1954 and TOV-112D cells were co-transfected with HA-KIBRA WT or 2CS and HA-Ub. The cell lysates were immunoprecipitated using an anti-HA antibody, followed by IB with an anti-Ub antibody. **F)** HCC1954 cells were transfected with V5-ZDHHC15, HA-KIBRA-WT or 2CS and then exposed to BAF-A1 10 nM for 4 h. Whole cell lysates (WCL) were subjected to IP with an anti-HA antibody followed by IB analysis for LAPM2, HA, and V5, respectively. **G)** The correlation between ZDHHC15 and WWC1 expression in ovarian cancer (GEPIA 2.0, R=-0.32, $P<0.05$). **H)** Immunohistochemical staining of KIBRA performed on paraffin-embedded xenograft tumor tissues. Scale bar: 100 μm. **I)** The correlation between ZDHHC15 and KIBRA expression in breast cancer tissues was determined using the two-sided Spearman test. GAPDH as a loading control. Data are representative of three independent experiments.

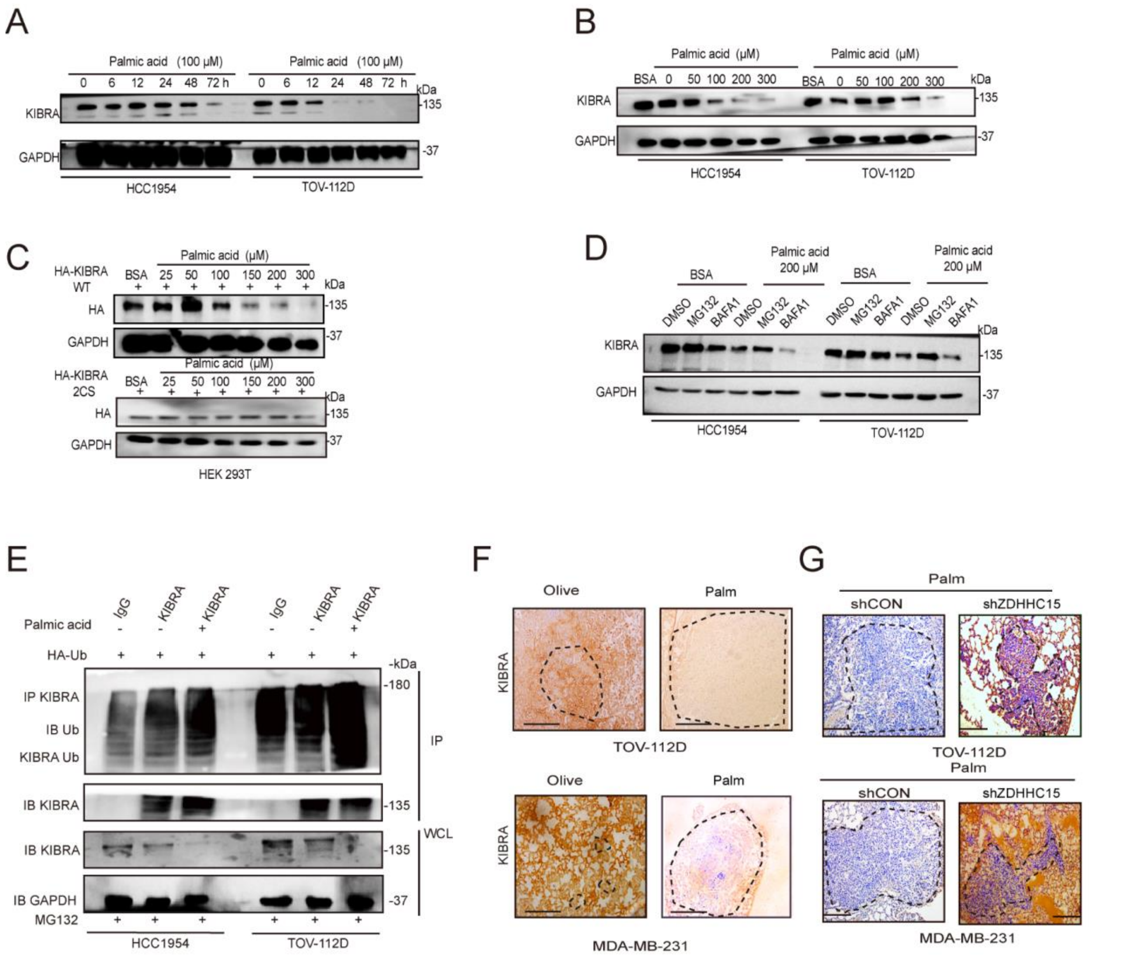

Figure S20. (Related to Figure 7) PA decreases the protein stability of KIBRA. (A-B) HCC1954 and TOV-112D cells were treated with different durations **(A)** and various concentrations **(B)** of palmitic acid. Immunoblotting (IB) analysis was performed to examine the expression levels of KIBRA. Bovine serum albumin (BSA) was used as a negative control. **C)** HCC1954 cells were transfected with HA-KIBRA WT or HA-KIBRA 2CS constructs, followed by treatment with 100 μM palmitic acid for 24 h. The protein expression of exogenous KIBRA was analyzed by IB. **D)** HCC1954 and TOV-112D cells were pretreated with bafilomycin A1 (BafA1) or MG132 for 4 h, followed by stimulation with or without 200 μM palmitic acid for 12 h. IB was performed to analyse the protein levels of KIBRA. **E)** HCC1954 and TOV-112D cells were treated

with 100 μM palmitic acid for 48 h. Cell lysates were immunoprecipitated using an anti-KIBRA antibody, followed by IB with an anti-Ubiquitin antibody. **F)** Immunohistochemical (IHC) staining of KIBRA performed on paraffin-embedded pulmonary transplantation tumor tissues. Scale bar: 100 μm. **G)** IHC staining of KIBRA in paraffin-embedded pulmonary transplantation tumor tissues from the indicated groups. Scale bar: 100 μm. Data are representative of three independent experiments.

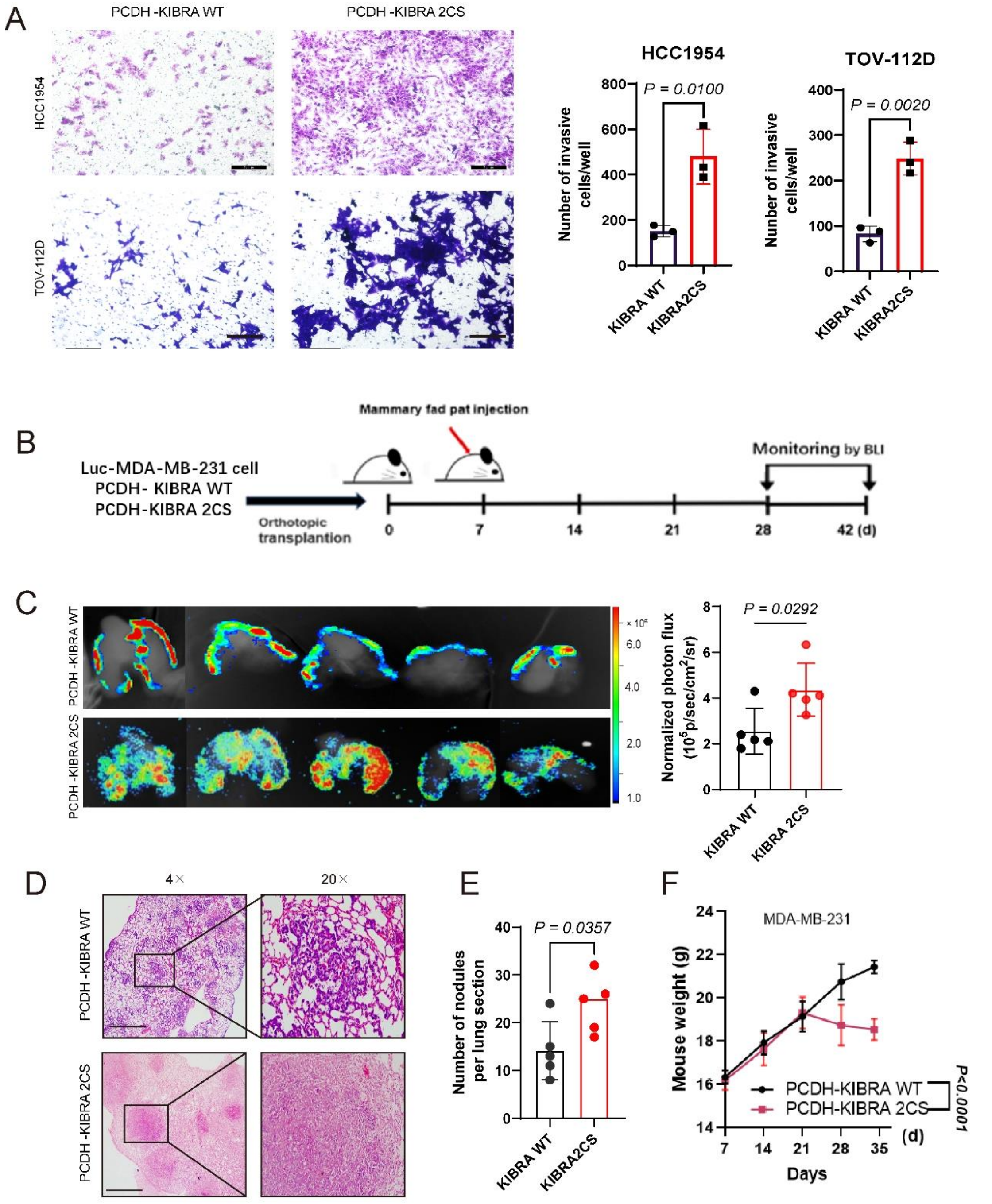

Figure S21. (Related to Figure 8) KIBRA 2CS mutant loses the ability to inhibit tumor metastasis. A) The invasion capability of TOV-112D and HCC1954cells with stable overexpress of KIBRA WT or 2CS was evaluated using the trans-well chamber, *P* values were determined by unpaired Student's t test (n=3). Scale bar: 100 µm. **(B, C).** Bioluminescent images (BLI)，quantification of lung metastasis tumours generated

from mice injected with MDA-MB-231 cells overexpressing KIBRA WT or 2CS in orthotopic transplantation models. (10 mice were randomly divided into two groups, n=5 mice in each group). **D)** Representative images of H&E staining (n=5 mice in each group). **(E, F)** The number of lung metastatic nodules **(E)**, and weight change **(F)** was counted and statistically analyzed, (n=5 mice in each group). *P* values were determined by the unpaired Student's t test for (A, C, E), two-way analysis of variance analysis for F.

.References

[1] K. Fang, S. Du, D. Shen, Z. Xiong, K. Jiang, D. Liang, J. Wang, H. Xu, L. Hu, X. Zhai, Y. Jiang, Z. Xia, C. Xie, D. Jin, W. Cheng, S. Meng, Y. Wang, *iScience* **2022**, *25*, 104618.

[2] K. Jiang, P. Liu, H. Xu, D. Liang, K. Fang, S. Du, W. Cheng, L. Ye, T. Liu, X. Zhang, P. Gong, S. Shao, Y. Wang, S. Meng, *Oncogene* **2020**, *39*, 5015.

[3] A. K. Lakkaraju, L. Abrami, T. Lemmin, S. Blaskovic, B. Kunz, A. Kihara, M. Dal Peraro, F. G. van der Goot, *EMBO J.* **2012**, *31*, 1823.

[4] X. Shao, A. Xu, W. Du, T. Xu, Y. Huang, Z. Xia, W. Wang, M. Cai, X. Zhang, J. Zhang, J. Cao, X. Xu, B. Yang, Q. He, M. Ying, *Blood* **2023**, *142*, 365.

[5] L. Hu, D. Shen, D. Liang, J. Shi, C. Song, K. Jiang, M. Ren, S. Du, W. Cheng, J. Ma, S. Li, X. Bi, M. P. Barr, Z. Fang, Q. Xu, W. Li, H. Piao, S. Meng, *Cancer Lett.* **2020**, *493*, 156.

[6] T. Li, J. Fan, B. Wang, N. Traugh, Q. Chen, J. S. Liu, B. Li, X. S. Liu, *Cancer Res.* **2017**, *77*, e108.
